# How an essential Zn2Cys6 transcription factor PoxCxrA regulates cellulase gene expression in ascomycete fungi?

**DOI:** 10.1186/s13068-019-1444-5

**Published:** 2019-05-03

**Authors:** Lu-Sheng Liao, Cheng-Xi Li, Feng-Fei Zhang, Yu-Si Yan, Xue-Mei Luo, Shuai Zhao, Jia-Xun Feng

**Affiliations:** State Key Laboratory for Conservation and Utilization of Subtropical Agro-bioresources, Guangxi Research Center for Microbial and Enzyme Engineering Technology, College of Life Science and Technology, Guangxi University, 100 Daxue Road, Nanning, 530004 Guangxi People’s Republic of China

**Keywords:** Zn2Cys6 transcription factor, PoxCxrA, DNA binding, Cellulase gene expression, *Penicillium oxalicum*

## Abstract

**Background:**

Soil ascomycete fungi produce plant-biomass-degrading enzymes to facilitate nutrient and energy uptake in response to exogenous stress. This is controlled by a complex signal network, but the regulatory mechanisms are poorly understood. An essential Zn2Cys6 transcription factor (TF) PoxCxrA was identified to be required for cellulase and xylanase production in *Penicillium oxalicum*. The genome-wide regulon and DNA binding sequences of PoxCxrA were further identified through RNA-Sequencing, DNase I footprinting experiments and in vitro electrophoretic mobility shift assays. Moreover, a minimal DNA-binding domain in PoxCxrA was recognised.

**Results:**

A PoxCxrA regulon of 1970 members was identified in *P*. *oxalicum*, and it was displayed that PoxCxrA regulated the expression of genes encoding major plant cell wall-degrading enzymes, as well as important cellodextrin and/or glucose transporters. Interestingly, PoxCxrA positively regulated the expression of a known important TF *PoxClrB*. DNase I footprinting experiments and in vitro electrophoretic mobility shift assays further revealed that PoxCxrA directly bound the promoter regions of *PoxClrB* and a cellobiohydrolase gene *cbh1* (*POX05587*/*Cel7A-2*) at different nucleic acid sequences. Remarkably, PoxCxrA autoregulated its own *PoxCxrA* gene expression. Additionally, a minimal 42-amino-acid PoxCxrA DNA-binding domain was identified.

**Conclusion:**

PoxCxrA could directly regulate the expression of cellulase genes and the regulatory gene *PoxClrB* via binding their promoters at different nucleic acid sequences. This work expands the diversity of DNA-binding motifs known to be recognised by Zn2Cys6 TFs, and demonstrates novel regulatory mechanisms of fungal cellulase gene expression.

**Electronic supplementary material:**

The online version of this article (10.1186/s13068-019-1444-5) contains supplementary material, which is available to authorized users.

## Background

Soil microorganisms that drive biogeochemical processes, including nutrient cycling, disease suppression and water dynamics, are critical to ecological balance and climate change, and highly sensitive to precipitation seasonality. In soils of tropical and subtropical forests that are hot spots of global carbon cycling, fungal strains from Ascomycota (66.3%) and Basidiomycota (31.3%) are particularly abundant [1], among which *Penicillium* and *Trichoderma* are the dominant decomposers, producing carbohydrate-active enzymes (CAZymes) that degrade plant cell walls into soluble sugars for nutrient availability [2, 3].

Cells sense and respond to extracellular stimuli such as starvation and stress, and these processes are controlled by complex signalling networks, resulting in multi-faceted physiological and morphological changes, including cell metabolism and growth. During starvation, cells attempt to reduce energy and carbon requirements, recycle structural components and undergo specialized processes such as sporulation, autophagy, apoptosis or necrosis [4]. Fungal cells control CAZyme production by utilising complex nutrient-sensing pathways comprising numerous sensors and receptors such as kinases, transcription factors (TFs) and their targets [5]. However, the overall process remains poorly understood.

*Penicillium oxalicum* produces integrated cellulolytic enzymes that degrade insoluble cellulose, and displays a preference for particular carbon sources. When *P*. *oxalicum* grows in the presence of glucose, expression of cellulolytic enzyme-encoding genes is repressed via carbon catabolite repression (CCR). Among the TFs involved in CCR, the core zinc finger TF CreA/Cre-1 is the one best studied in filamentous fungi, including *P*. *oxalicum*. CreA directly or indirectly represses the expression of all major CAZyme genes and their regulatory genes that are involved in the degradation of plant cell walls in the presence of glucose [6, 7].

When cellulose is present as a sole carbon source, induction of cellulolytic genes in *P*. *oxalicum* is dependent on a few essential TFs including the Zn2Cys6 TFs PoxCxrA and PoxClrB. Individual deletion of *PoxCxrA* and *PoxClrB* results in almost no cellulase production by *P*. *oxalicum* [2, 8]. Clr2, a homolog of PoxClrB in *Neurospora crassa*, binds to a DNA sequence identical to that bound to by the *Saccharomyces cerevisiae* TF Gal4p (CGGN11CCG) [9]. Although PoxCxrA binds directly to the promotor regions of major cellulase and xylanase genes [8], the DNA element recognised by PoxCxrA remains unknown.

In the present study, we employed high-throughput sequencing of transcripts (RNA-seq) to analyse transcriptional levels of genes in *P*. *oxalicum* deletion mutant ∆*PoxCxrA* following exposure to Avicel in comparison with the parental strain ∆*PoxKu70* to identify the regulon of *PoxCxrA*. Moreover, we identified two different DNA motifs recognised by PoxCxrA, as well as the minimal DNA-binding domain of PoxCxrA via in vitro DNase I footprinting and electrophoretic mobility shift assay (EMSA) experiments.

## Results

### *PoxCxrA* positively regulates the expression of most genes encoding plant-cell-wall-degrading enzymes in *P*. *oxalicum*

In previous work, an essential TF PoxCxrA was found to be required for cellulase production in *P*. *oxalicum*, when subjected to cellulose as a carbon source [2, 8]. However, its regulon is not yet fully understood. To comprehensively explore the regulatory roles of *PoxCxrA* in *P*. *oxalicum*, RNA-Seq was employed to analyse the transcriptomes of the *P*. *oxalicum* mutant strain ∆*PoxCxrA* and the parental strain ∆*PoxKu70* cultured in medium containing Avicel as the sole carbon source for 24 h after a shift from glucose. A total of 21–23 million clean reads with a length of 100 bp were generated across all samples (Additional file 1: Table S1), > 90% of which were successfully mapped into the genome of *P*. *oxalicum* wild-type strain HP7-1 [2]. To evaluate the correlations among the three biological replicates for each strain, the Pearson’s correlation coefficient (*r*) was calculated. The resulting high *r* value (> 0.85; Additional file 2: Figure S1) suggests that the transcriptomic data were reliable and suitable for further analysis.

**Fig. 1:**
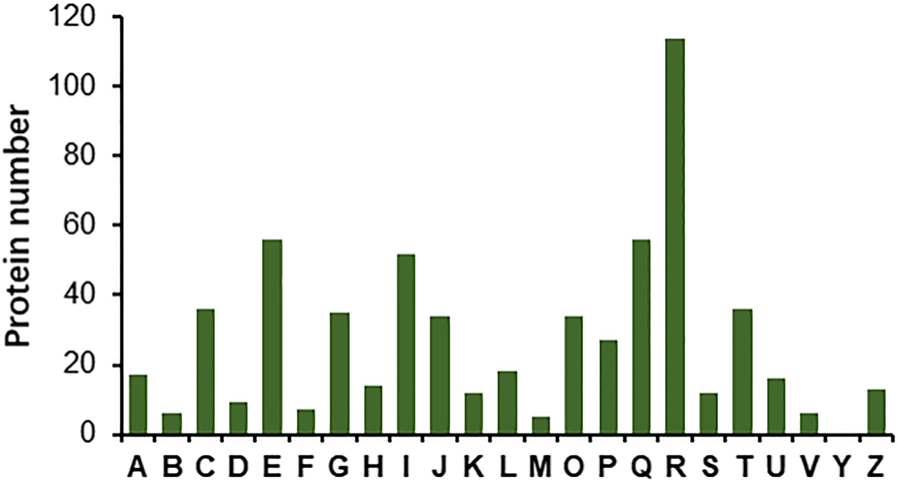
KOG annotation of DEGs in the *PoxCxrA* regulon. DEGs were selected as a |log2 fold change| ≥ 1 and a *p* value ≤ 0.05 as thresholds. *DEGs* differentially expressed genes, *KOG* Eukaryotic Orthologous Group

Comparative transcriptomic profiling identified 1970 DEGs in deletion mutant ∆*PoxCxrA*, compared with the parental strain ∆*PoxKu70*, according to the |log2 fold change| ≥ 1 and *p* value ≤ 0.05 thresholds (Additional file 3: Table S2), which were defined as the *PoxCxrA* regulon. The *PoxCxrA* regulon included 1010 genes down-regulated compared with ∆*PoxKu70*. Functional annotation based on Eukaryotic Orthologous Group (KOG) classification revealed that most of these DEGs were involved in primary and secondary metabolism (category E, amino acid transport and metabolism; category Q, secondary metabolite biosynthesis, transport and catabolism), and fell into the general function prediction only category (category R) (Fig. 1).

**Fig. 2:**
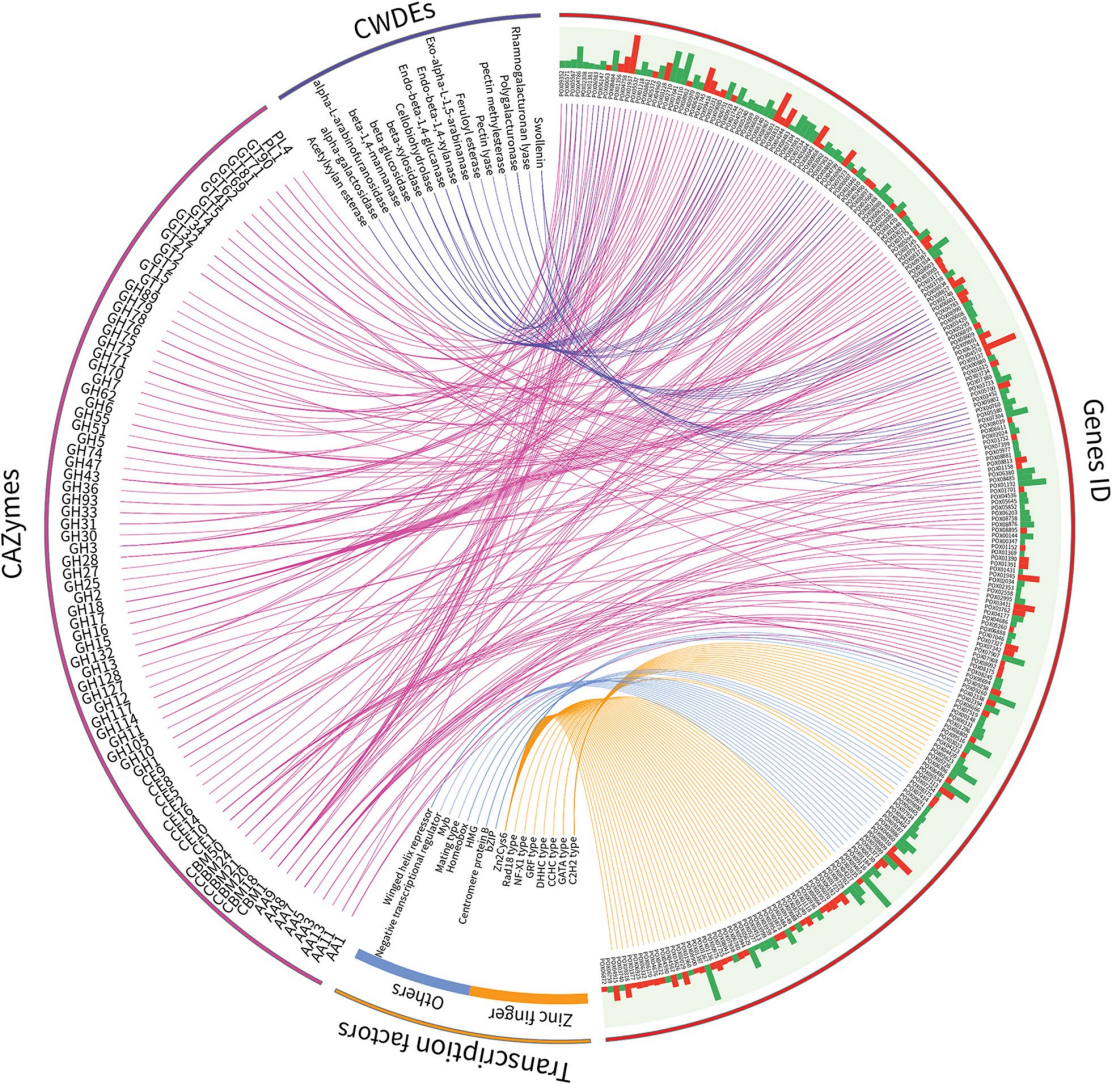
Regulation of genes encoding carbohydrate-active enzymes and putative transcription factors by *PoxCxrA*. Histogram indicates relative transcription levels of DEGs in *P. oxalicum* mutant ∆*PoxCxrA* exposed to Avicel compared with the parental strain ∆*PoxKu70*. *CWDEs* plant cell walls-degrading enzymes, *CAZymes* carbohydrate-active enzymes

Expression of genes encoding three key module signal carriers (i.e. sugar transporters), TFs, and functional proteins (i.e. CAZymes) formed a complex signalling network, and this was investigated in detail. Among the 1970 DEGs, annotation revealed that 154 DEGs encoded CAZymes, including seven auxiliary activity families, six carbohydrate-binding module families, eight carbohydrate esterase families, 42 glycoside hydrolase families and 14 glycosyltransferase families, as well as two polysaccharide lyase families. Among them, 46 genes encoding putative plant cell wall-degrading enzymes (CWDEs) were detected. Importantly, most key cellulase and xylanase genes in *P*. *oxalicum* were included in this DEG set, such as three cellobiohydrolase (CBH) genes *POX05587*/*Cel7A*-*2* (also called *cbh1*), *POX02490*/*Cel7A*-*1* and *POX04786*/*Cel6A*, three endo-β-1,4-glucanase (EG) genes *POX06147*/*Cel5A*, *POX02740* and *POX06983*, four β-glucosidase (BGL) genes *POX00968*, *POX03062*, *POX07963* and *POX08882*, five xylanase (Xyn) genes *POX00063/xyn10A*, *POX06783/xyn11A*, *POX06601*, *POX08484*/*Xyn11B* and *POX08990*, two lytic polysaccharide monooxygenase *POX02308*/*aa9A* and *POX08897* (Additional file 3: Table S2), which accounted for approximately 70% of cellulase and xylanase genes in the whole genome of *P. oxalicum* wild-type strain HP7-1 (Fig. 2).

Of these 154 CAZyme-encoding genes, 95 were down-regulated (− 7.0 < log2 fold change < − 1.0) compared with the parental strain ∆*PoxKu70*. Interestingly, expression of all 17 cellulase and xylanase genes described above were down-regulated in the ∆*PoxCxrA*, except for two genes (*POX00968* and *POX02490*/*Cel7A*-*1*) (Fig. 2).

### *PoxCxrA* regulates the expression of genes encoding cellodextrin and glucose transporters

In complex signal transduction pathways, transporters/sensors play important roles, as demonstrated for cellodextrin transporters PoxCdtC and PoxCdtD in cellulase production in *P*. *oxalicum* [10]. Among the 1970 DEGs, 34 were annotated as sugar/inositol transporters (IPR003663) and/or major facilitators, or sugar transporter-like (IPR005828). Remarkably, two known genes (*POX06051*/*PoxCdtC* and *POX05915*/*PoxCdtD*) encoding cellodextrin transporters PoxCdtC and PoxCdtD, three genes (*POX07576*, *POX07227* and *POX08783*) encoding *N. crassa* glucose transporter RCO3-like [11], and GLT1 and HGT-2 proteins [12], sharing 51–76% sequence identity, were included.

**Fig. 3:**
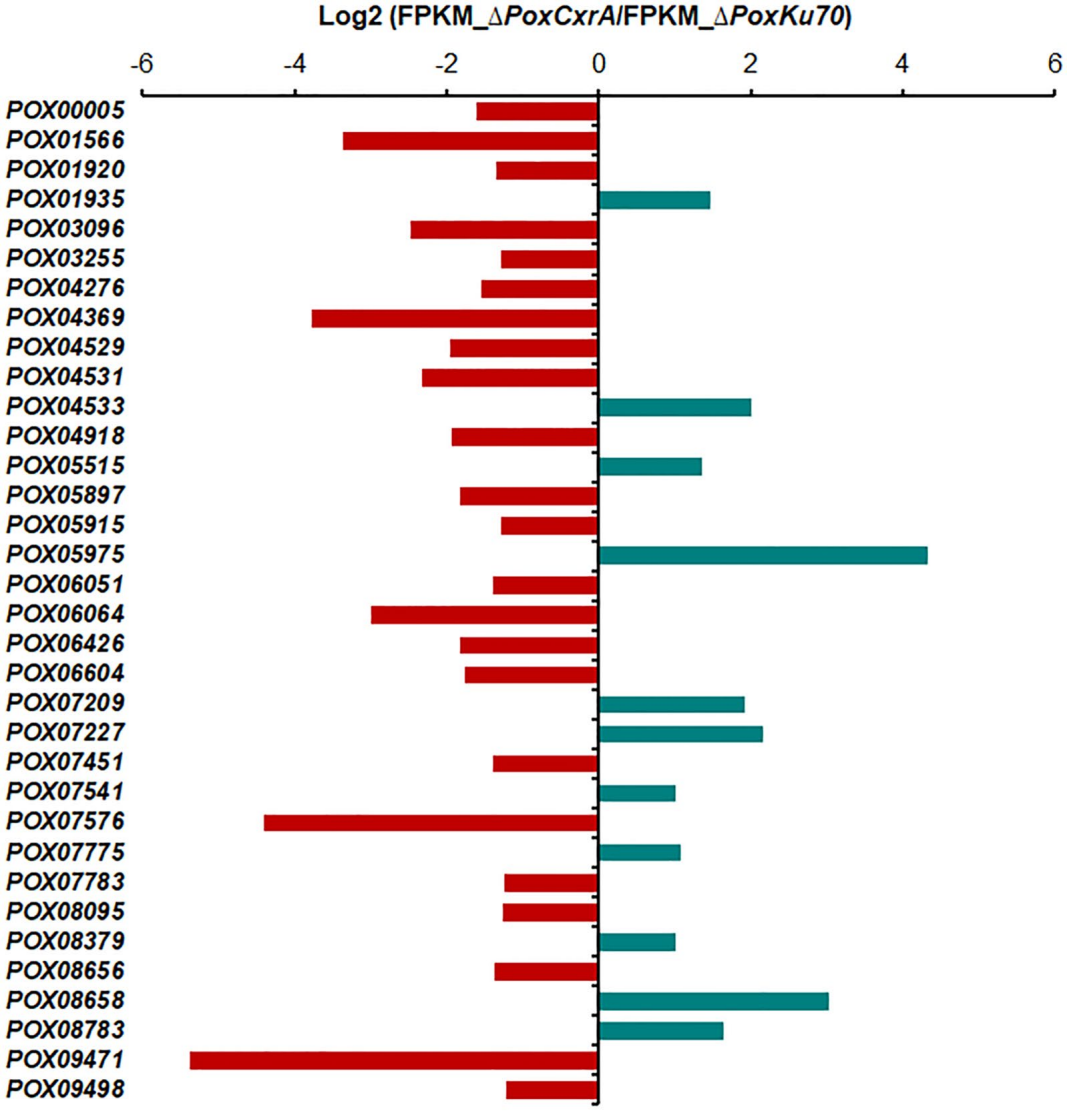
Regulation of putative transporter genes by *PoxCxrA*. Values in heatmaps were calculated by log2 (Gene_FPKM in ∆*PoxCxrA*/Gene_FPKM in ∆*PoxKu70*). *FPKM* fragments per kilobase of exon per million fragments mapped

The regulon of *PoxCxrA* included 23 down-regulated genes encoding cellodextrin and glucose transporters (− 5.4 < log2 fold change < − 1.2). Among them, transcriptional levels of *POX06051*/*PoxCdtC*, *POX05916*/*PoxCdtD* and *POX07576* were down-regulated in the ∆*PoxCxrA* compared with ∆*PoxKu70*, with a log2 fold change from − 4.4 to − 1.3 (Fig. 3 and Additional file 3: Table S2). In contrast, *POX07227* and *POX08783* transcript levels increased 4.5–3.1-fold in ∆*PoxCxrA* (Fig. 3).

### *PoxCxrA* regulates the expression of genes encoding TFs controlling cellulase gene expression

Genes encoding putative TFs among the 1970 DEGs were explored, and 88 candidates were identified, mostly encoding zinc finger proteins such as Zn2Cys6, C2H2 and CCHC families. Among the 88 TFs, 14 were known to regulate cellulase production in filamentous fungi, including eight activators (POX00331/FlbC, POX01184, POX01960/PoxClrB, POX02484, POX04420/PoxCxrB, POX05726, POX08415/NsdD and POX08910) and six repressors (POX00864, POX04860/PDE_07199, POX06534/BrlA, POX06759, POX07254/CreA and POX08375/Ace1) [2, 8, 13–17]. Comparative transcriptomics indicated that 45 down-regulated genes encoding putative TFs (− 9.6 < log2 fold change < − 1.0). Interestingly, deletion of *PoxCxrA* resulted in down-regulation of 11 known regulatory genes (*POX00331*/*FlbC*, *POX01184*, *POX01960*/*PoxClrB*, *POX02484*, *POX04420*/*PoxCxrB*, *POX04860*/*PDE_07199*, *POX05726*, *POX06534*/*BrlA*, *POX06759*, *POX07254*/*CreA* and *POX08415*/*NsdD*; − 6.8 < log2 fold change < − 1.1), and up-regulation of known regulatory genes *POX08910*, *POX00864* and *POX08375*/*Ace1* (1.1 < log2 fold change < 1.3), compared with the ∆*PoxKu70* transcriptome (Fig. 2 and Additional file 3: Table S2).

### PoxCxrA and PoxClrB dynamically regulates the expression of one another

**Fig. 4:**
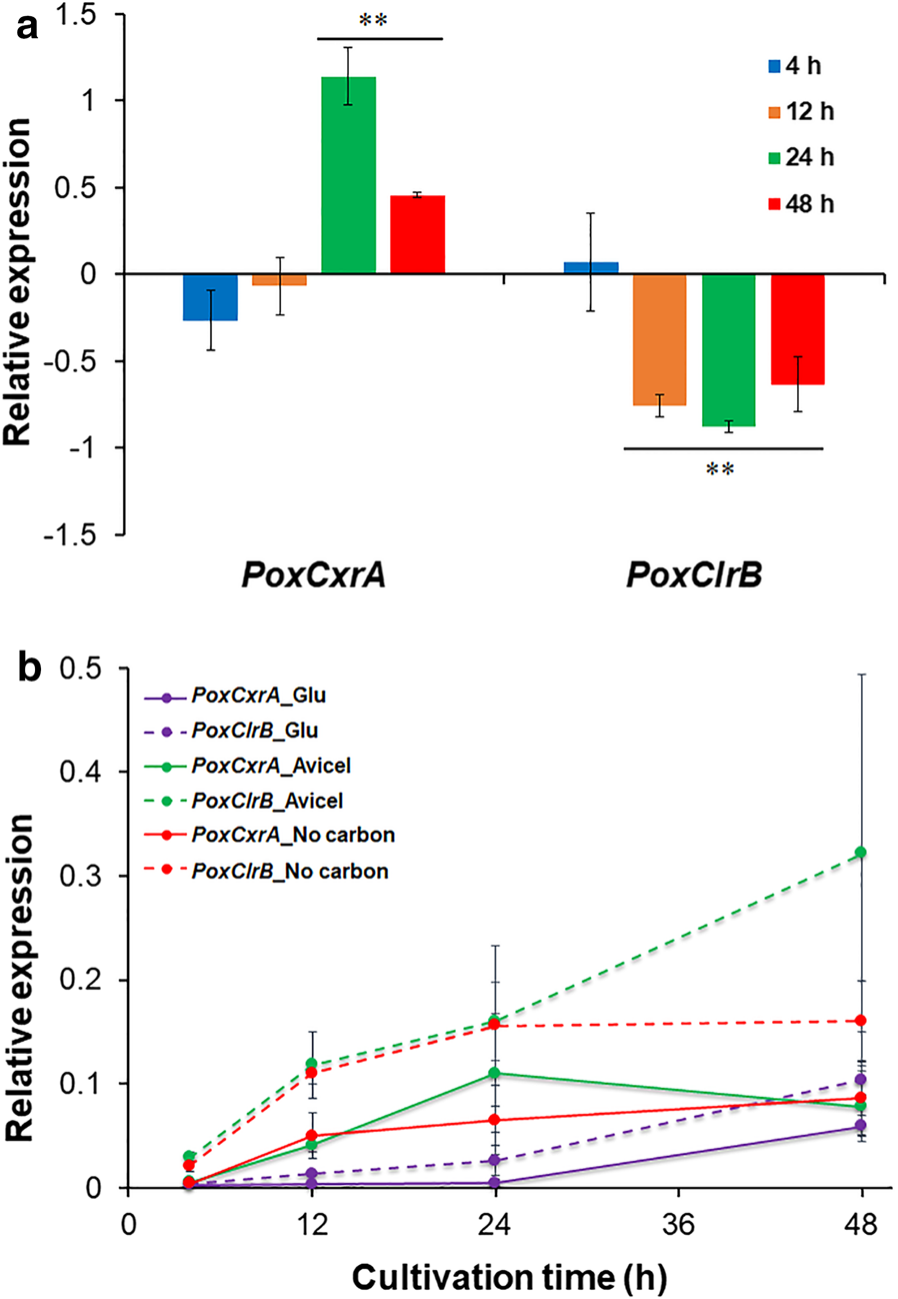
Expression of *PoxCxrA*, *PoxClrB* and major cellulase genes in *P. oxalicum*, and their regulation. **a** Expression of *PoxCxrA* and *PoxClrB* in the mutant strain ∆*PoxClrB* and ∆*PoxCxrA* subjected to Avicel as the sole carbon source, respectively. **b** Transcriptional levels of genes *PoxCxrA* and *PoxClrB* in ∆*PoxKu70* in the presence of different carbon sources. Gene expression levels were investigated in *P*. *oxalicum* strains cultivated on Avicel at four different time points (4, 12, 24 and 48 h) after a shift from glucose by real-time quantitative reverse-transcription PCR. Relative expression on the *y*-axis indicates differences in values for transcription between tested and reference genes, or between tested genes in deletion mutants and the parental strain Δ*PoxKu70*. *p ≤ 0.05 and **p ≤ 0.01 indicate differences among samples assessed by Student’s *t*-tests. All experiments were carried out with at least three independent replicates

Interestingly, PoxCxrA regulated the expression of a key regulatory gene *PoxClrB* through RNA-Seq. To further elucidate this regulation, RT-qPCR was employed. When Δ*PoxCxrA* was exposed to Avicel, transcription of *PoxClrB* was down-regulated to some extent (2.7- to 8.2-fold) after 4 h (*p *≤ 0.05, Student’s *t* test) compared with that in ∆*PoxKu70*. In contrast, *PoxCxrA* expression increased by 1.5- to 2.1-fold in ∆*PoxClrB* during the latter stages of cultivation (24–48 h after induction; Fig. 4a).

### Expression of both *PoxCxrA* and *PoxClrB* is induced by cellulose

When *P*. *oxalicum* strain ∆*PoxKu70* was transferred into medium containing Avicel (induced state), *PoxCxrA* and *PoxClrB* exhibited similar transcriptional levels to those without a carbon source (de-repressed state) during the early induction stage (0–12 h), but only the transcriptional level of *PoxClrB* was higher than that without a carbon source during later stage (48 h). The expression of both *PoxCxrA* and *PoxClrB* on Avicel were higher than that on glucose (repressed state). Surprisingly, *PoxCxrA* expression on Avicel increased by ~ 70% compared with its expression without a carbon source, but only at 24 h. Expression level of *PoxClrB* in ∆*PoxKu70* was higher than those of *PoxCxrA* during all states (induced, repressed and de-repressed). *PoxCxrA* expression under Avicel induction increased before 24 h, but reduced after 24 h (Fig. 4b).

### PoxCxrA directly binds to the promoter regions of *PoxClrB* and *PoxCxrA*, and genes encoding cellodextrin and glucose transporters

**Fig. 5:**
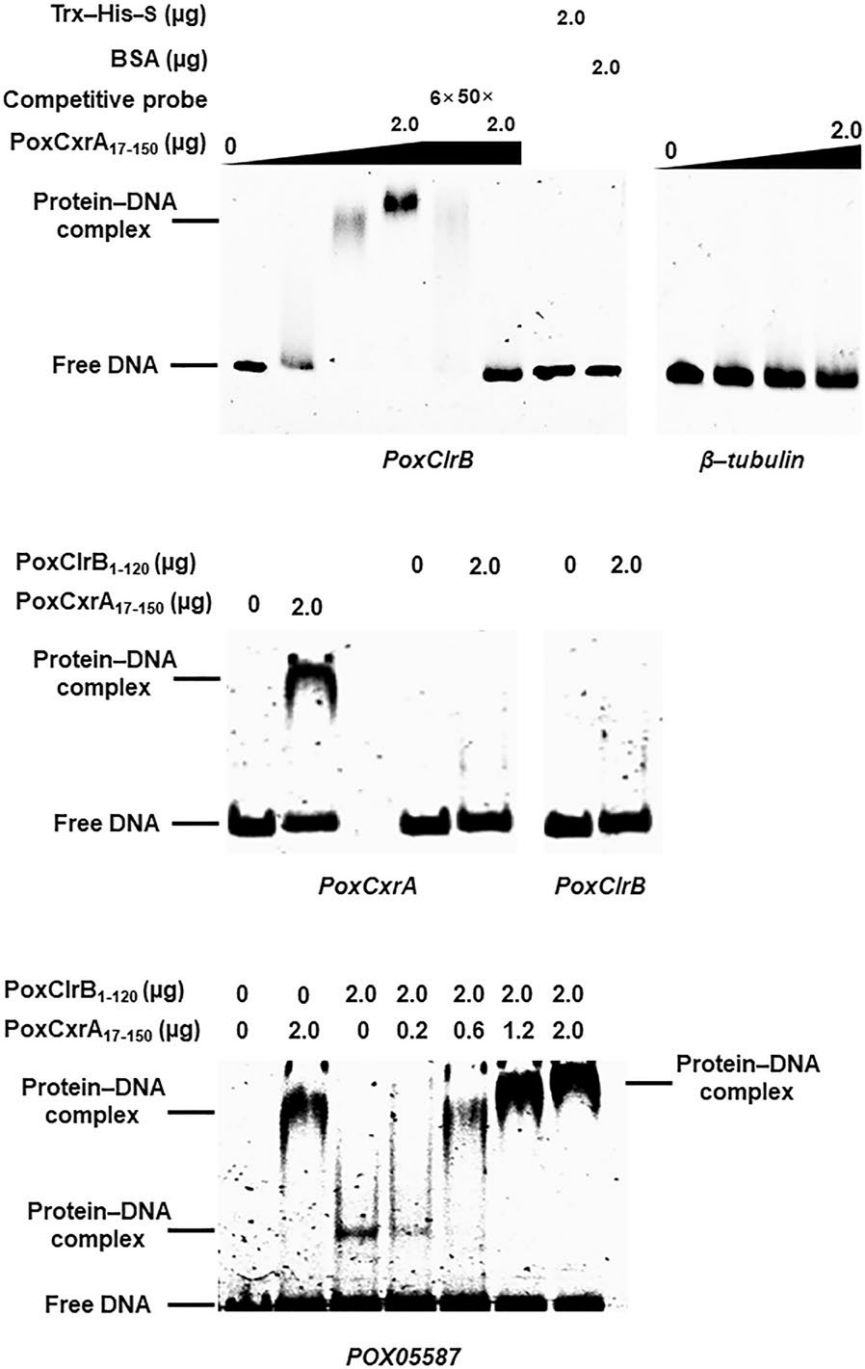
Electrophoretic mobility shift assay (EMSA) analysis of the binding of PoxCxrA_17–150_ and PoxClrB_1–120_ to the promoter regions of *PoxCxrA*, *PoxCxrB* and *POX05587/Cel7A*-*2*

To further confirm whether PoxCxrA directly or indirectly regulates *PoxClrB* expression, in vitro EMSA experiments were employed. The putative DNA-binding domain PoxCxrA_17–150_ was recombinantly expressed and purified by fusing with thioredoxin (Trx), His and S-tags. A 300-bp DNA fragment from the promoter region of *PoxClrB* tagged with 6-carboxyfluorescein (6-FAM) was amplified using specific primers (Additional file 4: Table S3) and used as the probe for EMSA experiments. A DNA fragment from the promoter region of the β-tubulin gene *POX05989* was used as a control. The results revealed that a complex was formed between PoxCxrA_17–150_ [8] and the promoter region of *PoxClrB*, and its concentration increased with an increasing amount of fusion protein (1.0–2.0 µg). Bovine serum albumin (BSA) and Trx–His–S did not interact with the *PoxClrB* probe, and there was no interaction between PoxCxrA_17–150_ and the control *POX05989* promoter region. Competitive EMSA experiments revealed that the concentration of the complex decreased gradually with an increasing amount of protein-binding DNA fragment without 6-FAM (Fig. 5), suggesting that PoxCxrA_17–150_ specifically bound the promoter region of *PoxClrB*.

Each EMSA experiment comprised different DNA-binding domains (PoxCxrA_17–150_, PoxClrB_1–120_ or both; 0–2 µg) and 50 ng of the target probe labelled with 6-FAM. Probes without 6-FAM, and the β-tubulin gene *POX05989*, were used as competitive probes and negative controls, respectively. The fusion protein Trx–His–S purified from *Escherichia coli* cells harbouring the empty plasmid pET32a (+), and BSA alone, were used as protein controls.

The RT-qPCR data described above revealed that PoxClrB negatively regulated *PoxCxrA* expression during the latter stages of induction, but the regulatory mode was unclear. In vitro binding experiments showed that the putative DNA-binding domain of PoxClrB_1–120_ was unable to bind to the promoter region of the *PoxCxrA* gene (Fig. 5), indicating that PoxClrB indirectly regulates transcription of *PoxCxrA* in *P*. *oxalicum*. EMSA experiments also showed that PoxCxrA_17–150_ bound to the promoter region of its own gene, but PoxClrB_1–120_ did not, suggesting the autoregulation of *PoxCxrA*. When a mixture of PoxCxrA_17–150_ and PoxClrB_1–120_ was treated with probe *POX05587/Cel7A*-*2*, a band representing a larger protein–DNA complex than that formed by either PoxCxrA_17–150_ or PoxClrB_1–120_ individually was observed (Fig. 5), indicating that the binding motifs recognised by PoxCxrA_17–150_ and PoxClrB_1–120_ are distinct.

**Fig. 6:**
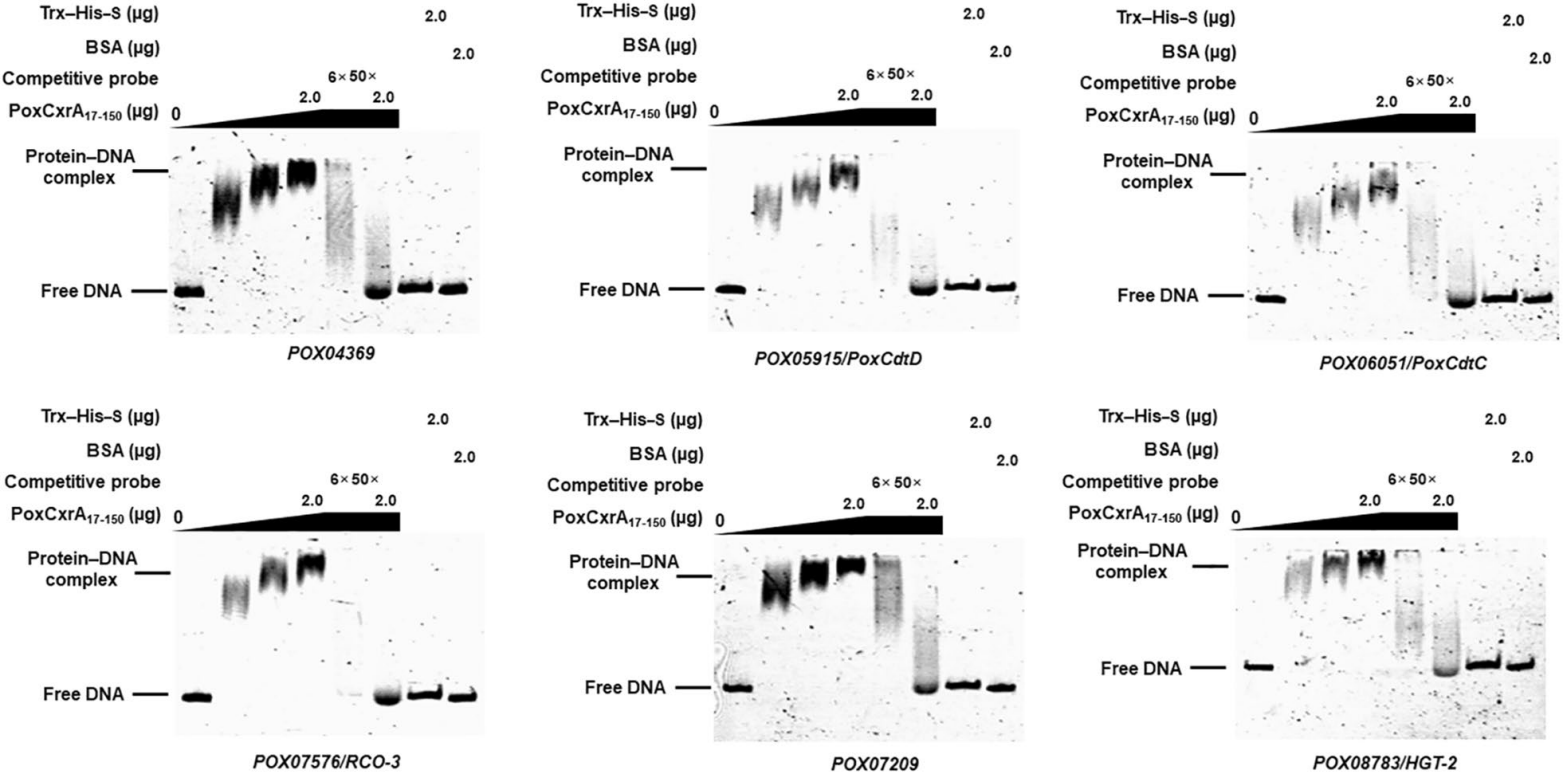
EMSA analysis of the interactions between the DNA-binding domain PoxCxrA_17–150_ and the promoter regions of genes encoding putative sugar transporters. Each EMSA experiment comprised PoxCxrA_17–150_ (0–2 µg) and 50 ng of target probes labelled with 6-carboxyfluorescein (6-FAM). Probes without 6-FAM, and the promoter of β-tubulin gene *POX05989*, were used as competitive probes and negative control, respectively. The fusion protein Trx–His–S purified from *E. coli* cells harbouring the empty plasmid pET32a (+), and BSA alone, were used as protein controls

Intriguingly, genes encoding cellodextrin and glucose transporters were included in the *PoxCxrA* regulon. EMSA experiments revealed that PoxCxrA_17–150_ could bind to the promoter regions of genes encoding cellodextrin transporters PoxCdtC and PoxCdtD, and putative glucose transporters POX07576/RCO-3, POX08783/HGT-2, POX07209 and POX04369 (Fig. 6).

### Different PoxCxrA-binding motifs are present in the promoter regions of target genes *POX05587/Cel7A*-*2* and *PoxClrB*

**Fig. 7:**
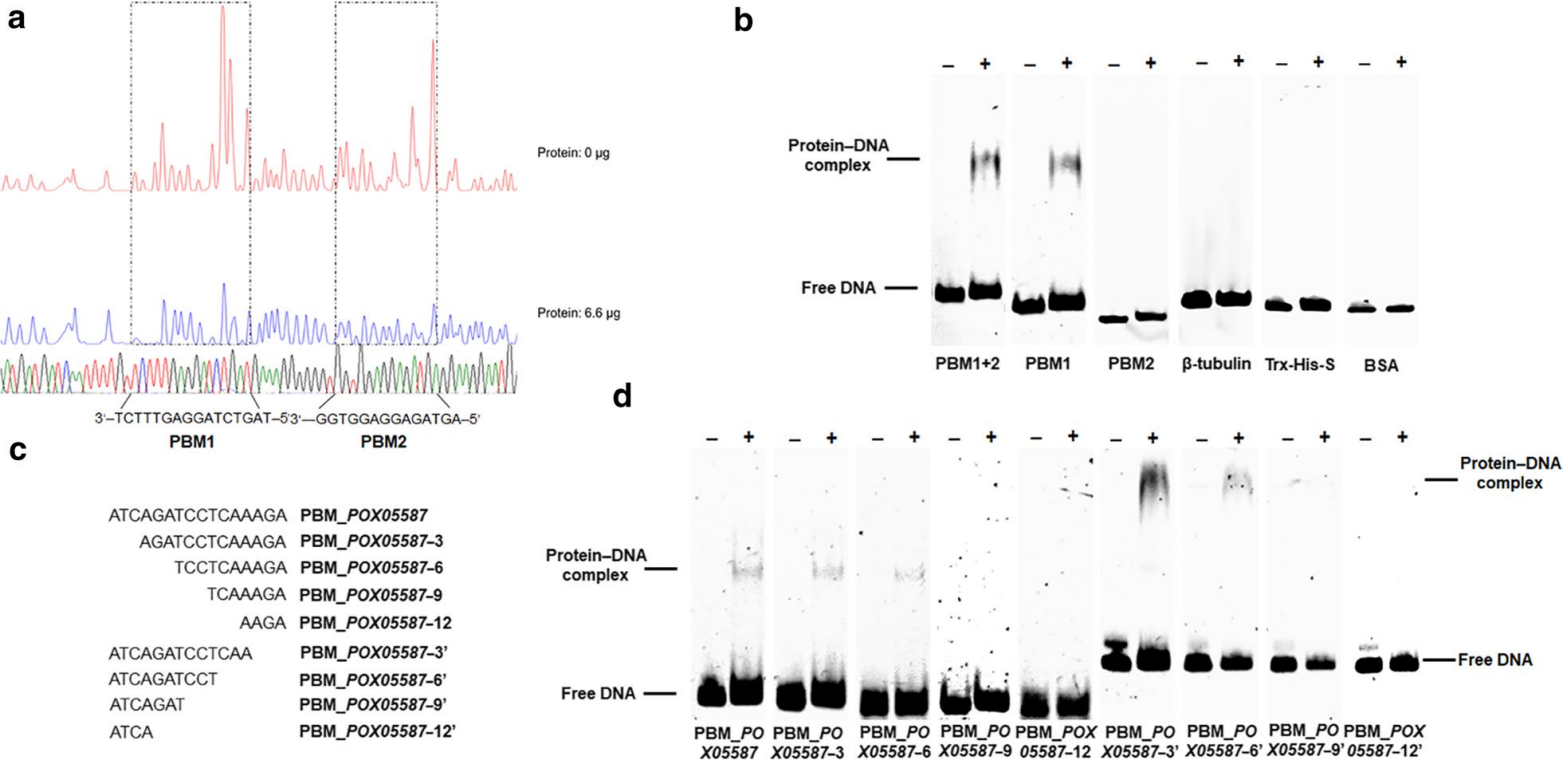
The PoxCxrA_17–150_-binding sequence in the promoter region of cellobiohydrolase gene *POX05587*/*Cel7A*-*2*. **a** DNase I footprinting experiment results. **b** EMSA confirmation of PBM_*POX05587*. **c** Schematic diagram of PBM_*POX05587* truncation. **d** Key nucleotides in PBM_*POX05587* confirmed via EMSA experiments. Each EMSA experiment comprised 2 µg of PoxCxrA_17–150_ and 50 ng of truncated PBM_*POX05587* as probe labelled with 6-carboxyfluorescein. The promoter of β-tubulin gene *POX05989* was used as a probe control. The fusion protein Trx–His–S purified from *E. coli* cells harbouring the empty plasmid pET32a (+), and BSA alone, were used as protein controls. ‘−’ and ‘+’ represent EMSA experiments without and with DBD PoxCxrA_17–150_, respectively

Based on the above results and those of previous work [8], PoxCxrA appears to regulate the expression of *cbh* gene *POX05587/Cel7A*-*2* and TF gene *PoxClrB* by directly binding to their promoters. In vitro EMSA and DNase I footprinting experiments were, therefore, performed to identify protein-binding motifs (PBMs) in the promoters of the target genes. An initial DNase I footprinting experiment was performed using a 100-bp DNA fragment corresponding to the upstream flanking sequence (starting from the transcription initiation ATG codon) labelled with 6-FAM at the 3′-terminus to identify the PBM of PoxCxrA_17–150_ in the promoter region of *POX05587*/*Cel7A*-*2*. The results revealed that two putative PBMs (PBM1, 5′-ATCAGATCCTCAAAGA-3′; PBM2, 5′-TCATCTCCTCCACC-3′) were protected by PoxCxrA_17–150_ to various degrees (Fig. 7a). Further EMSA experiments confirmed that PoxCxrA_17–150_ bound to probes containing PBM1 or PBM1 plus PBM2, but not PBM2 (Fig. 7b), suggesting that PBM1 contains the binding motif of PoxCxrA in the *POX05587*/*Cel7A*-*2* promoter region (PBM_*POX05587*).

**Fig. 8:**
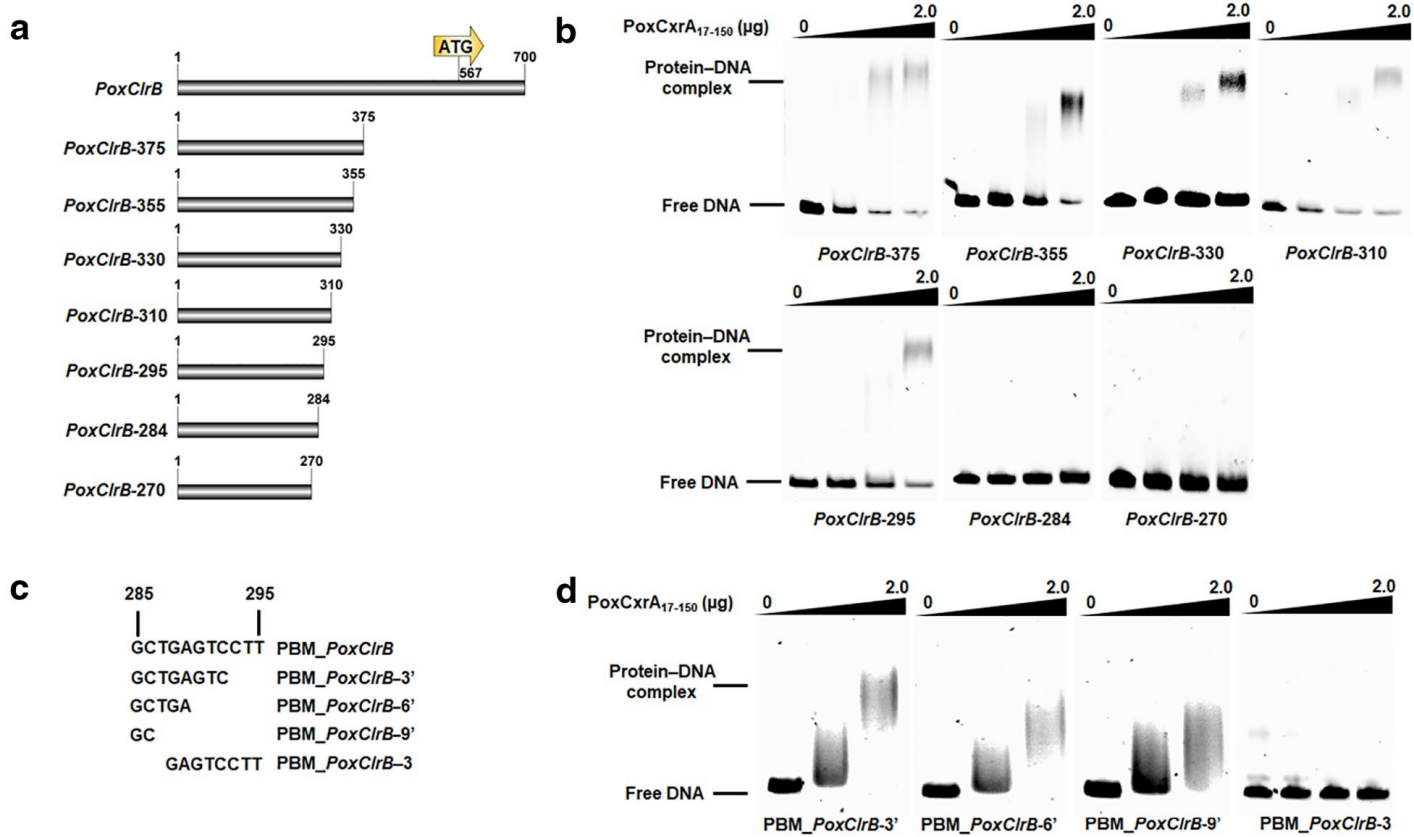
The PoxCxrA_17–150_-binding sequence in the promoter region of transcription factor gene *PoxClrB*. **a** Schematic diagram of putative PBM_*PoxClrB* truncation. **b** The PBM_*PoxClrB* sequence determined by in vitro EMSA. **c** EMSA analysis of key nucleotides in PBM_*PoxClrB*. Each EMSA experiments comprised 0–2 µg of PoxCxrA_17–150_ and 50 ng of truncated PBM_*PoxClrB* as probe labelled with 6-carboxyfluorescein. The promoter of β-tubulin gene *POX05989* was used as a probe control. The fusion protein Trx–His–S purified from *E. coli* cells harbouring the empty plasmid pET32a (+), and BSA alone, were used as protein controls

Meanwhile, a series of *PoxClrB* probes with different lengths (*PoxClrB*–375, *PoxClrB*–355, *PoxClrB*–330, *PoxClrB*–310, *PoxClrB*–295, *PoxClrB*–284 and *PoxClrB*–270 labelled with 6-FAM at the 5′-terminus) were amplified using specific primer pairs (Additional file 4: Table S3; Fig. 8a). EMSA experimental results revealed protein–DNA complexes between PoxCxrA_17–150_ and *PoxClrB*–375, *PoxClrB*–355, *PoxClrB*–330, *PoxClrB*–310 and *PoxClrB*–295, but not with the other probes or negative controls (Fig. 8b), suggesting that PoxCxrA binds the PBM_*PoxCrlB* in the promoter region of *PoxClrB* from positions 285–295 (5′-GCTGAGTCCTT-3′) (Fig. 8c). Compared with PBM_*POX05587*, three conserved sites were identified (i.e. GCTGAGTCCTT or ATCAGATCCTCAAAGA).

Subsequently, to characterise the key nucleotides in PBM_*PoxClrB* and PBM_*POX05587*, we generated a series of truncated PBM_*PoxClrB* and PBM_*POX05587* fragments as EMSA probes (Figs. 7c, 8c). The results revealed that protein–DNA complexes were formed between PoxCxrA_17–150_ and PBM_*POX05587*–3, PBM_*POX05587*–6, PBM_*POX05587*–3′, PBM_*POX05587*–6′, PBM_*PoxClrB*-3′, PBM_*PoxClrB*_6′ and PBM_*PoxClrB*_9′, and the concentrations of binding bands gradually decreased with the truncation of probe length (Figs. 7d, 8d). These results showed that the core nucleotides in PBM_*POX05587* and PBM_*PoxClrB* were TCCT and GC, respectively, whereas their flanking sequences were also required for PoxCxrA binding.

### Identification of a minimal DNA-binding domain of PoxCxrA

**Fig. 9:**
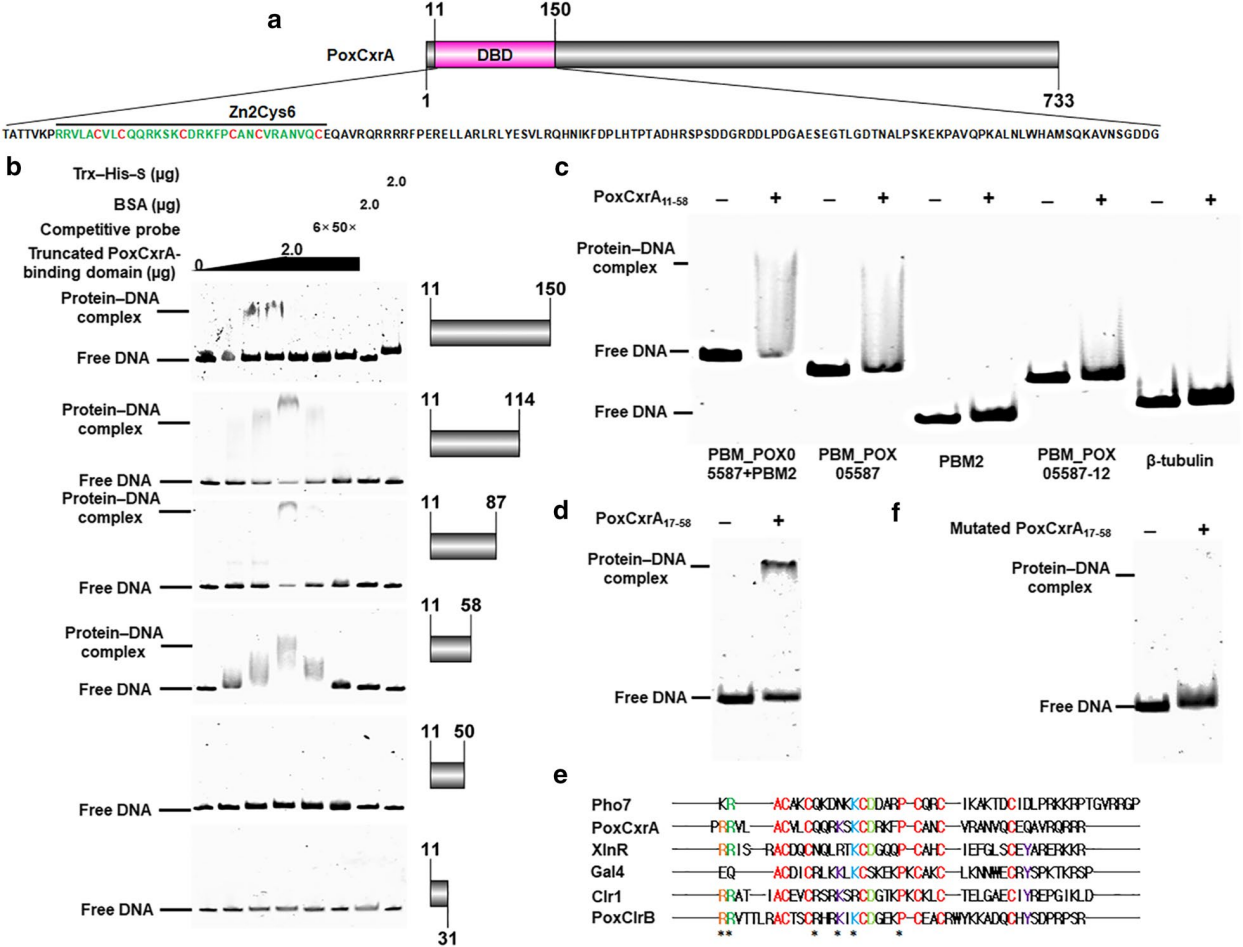
The DNA-binding domain (DBD) of PoxCxrA. **a** Schematic diagram of the PoxCxrA protein containing a Zn2Cys6 domain, and DBD truncation. **b** The minimal DBD_11–58_ determined via in vitro EMSA. **c** Interaction between PoxCxrA_11–58_ and PBM_*POX05587*. **d** Interaction between PoxCxrA_17–58_ and PBM_*POX05587*. Each EMSA experiment comprised 2 µg of the truncated PoxCxrA-binding domain and 50 ng of PBM_*POX05587* labelled with 6-carboxyfluorescein. The promoter of β-tubulin gene *POX05989* and PBM2 were used as control probes. The fusion protein Trx–His–S purified from *E. coli* cells harbouring the empty plasmid pET32a (+), and BSA alone, were used as protein controls. ‘−’ and ‘+’ represent EMSA without and with DBD of PoxCxrA_11–58_. ‘*’indicated the mutated amino acids into alanine in the mutated DBD of PoxCxrA_11–58_. **e** Alignment of DBD_17–58_ with known Zn2Cys6-containing transcription factors; PoxCxrA and PoxClrB are from *P*. *oxalicum* strain HP7-1 (accession numbers KY368172 and KU597415); Pho7, XlnR, Clr1 and Gal4 are from *Schizosaccharomyces pombe*, *P. oxalicum* strain 114-2 (EPS32714), *N. crassa* strain OR74A (XP_011394265) and *Saccharomyces cerevisiae* S288C (NP_015076), respectively. **f** Interaction between the mutated PoxCxrA_17–58_ and PBM_*POX05587*

The putative DNA-binding domain (DBD) of PoxCxrA used above (residues 17–150) was serially truncated from the C-terminus to identify a minimal functional DBD. To facilitate the purification of the expressed recombinant protein in *E. coli*, we first fixed at 11th amino acid of N-terminus. Recombinant proteins PoxCxrA_11–150_, PoxCxrA_11–114_, PoxCxrA_11–87_, PoxCxrA_11–58_ and PoxCxrA_11–31_ were produced in *E. coli* cells and purified (Fig. 9a, b), and in vitro EMSA experiments were carried out to investigate their ability to bind a 6-FAM-labelled 300-bp DNA fragment of the *POX05587*/*Cel7A*-*2* promoter region comprising the PoxCxrA binding site as EMSA probe. The results showed that each truncated protein bound the probe to form a protein–DNA complex that retarded electrophoretic mobility in gels, except for PoxCxrA_11–31_. The concentration of the protein–DNA complexes gradually increased with increasing protein loading (1.0–3.0 µg). Competitive EMSA experiments were simultaneously performed, and the results indicated that the amount of complex tended to reduce with increasing amounts of competitive probe without 6-FAM. BSA and Trx–His–S control proteins did not bind the *POX05587*/*Cel7A*-*2* promoter region (Fig. 9b). PoxCxrA_11–58_ was confirmed to bind to PMB_*POX05587* via in vitro ESMA experiments (Fig. 9c). Subsequently, the PoxCxrA_17–58_ was also expressed and purified. EMSA binding experiments indicated a clear band comprised of PoxCxrA_17–58_ and PMB_*POX05587* on the gel (Fig. 9d). Thus, the minimal 42-amino-acid segment of PoxCxrA_17–58_ suffices for DNA binding.

Subsequently, an alignment analysis of PoxCxrA_17–58_ with the DBDs in known Zn2Cys6-containing TFs including PoxClrB, Pho7, XlnR, Clr1 and Gal4 that came from *P*. *oxalicum* HP7-1 [2], *Schizosaccharomyces pombe* [18], *P. oxalicum* strain 114-2 [7], *N. crassa* strain OR74A [19] and *Saccharomyces cerevisiae* S288C [9], was respectively performed. The results found three pairs of highly conserved zinc-coordinating cysteines that are essential for protein binding [18] and relatively conserved flanking amino acids such as arginine (R), lysine (K), aspartic acid (D) and proline (P; Fig. 9e). The retained amino acids showed high diversity. Several relatively conserved amino acids (18th R, 19th R, 27th Q, 30th K, 32th K and 38th P) were respectively replaced by alanine to generate a mutated PoxCxrA_17–58_. In vitro EMSA further displayed that all the mutated PoxCxrA_17–58_ lost the ability to bind the PM_*POX05587* (Fig. 9f).

## Discussion

PoxCxrA is known to be a critical transcriptional activator in *P*. *oxalicum* exposed to cellulose as a carbon source, but its regulatory mechanism is unclear. Herein, we found that PoxCxrA regulates cellulase production in *P*. *oxalicum* by controlling the expression of *PoxClrB*, and further elucidated their regulatory network. PoxCxrA autoregulated the transcription of its own *PoxCxrA* gene, but PoxClrB did not. Moreover, the DNA-binding domain of PoxCxrA_17–150_ bound to the promoter regions of *PoxClrB* and *POX05587*/*Cel7A*-*2* at different binding sites (5′-ATCAGATCCTCAAAGA-3′ and 5′-GCTGAGTCCTT-3′, respectively) according to in vitro DNase I footprinting and EMSA experiments. A minimal functional DBD (residues 17–58) of PoxCxrA was identified. These findings provide novel insights into the regulatory mechanism governing cellulase gene expression in *P*. *oxalicum*.

The PoxCxrA regulon was identified, which included a number of members involved in primary and secondary metabolism. To withstand starvation caused by insoluble cellulose as the sole carbon source, expression of major cellulase genes in *P*. *oxalicum*, including genes encoding CBH1 (GH7) and EGs (GH5 and GH12), was rapidly up-regulated, resulting in abundant intra- and extracellular cellodextrin production in fungal cells [20–22]. Accumulated intracellular cellodextrin triggered signalling cascades that activated the expression of *PoxCxrA* and *PoxClrB*, subsequently resulting in activation of the expression of cellulase genes such as *POX05587*/*Cel7A*-*2*.

**Fig. 10:**
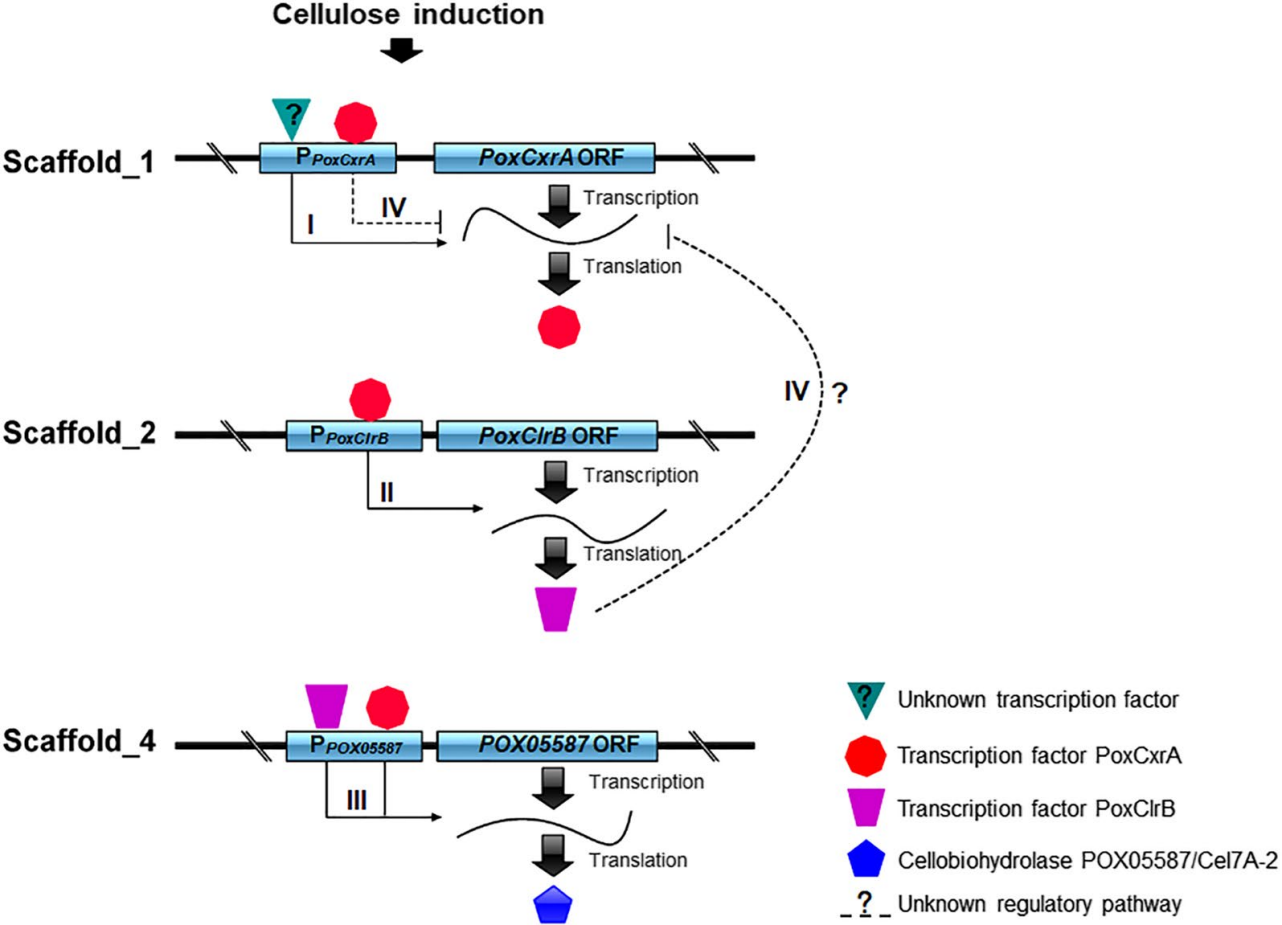
Postulated modules of the PoxCxrA and PoxClrB networks regulating cellulase gene expression in *P. oxalicum*. Lines with arrows indicate positive regulation, lines with flat ends indicate negative regulation, and dashed lines with flat ends represent unknown repressive expression. I–IV indicates predicted regulatory process over time

Intriguingly, PoxCxrA also directly activated *PoxClrB* expression, whereas PoxClrB indirectly repressed the transcription of *PoxCxrA* in *P*. *oxalicum*. Regrettably, exactly how *PoxCxrA* expression is repressed by PoxClrB remains unknown (Fig. 10). In the early phase of *P*. *oxalicum* exposed to Avicel, both *PoxCxrA* and *PoxClrB* were transcribed at a background level. Expression of *PoxCxrA* was first up-regulated at the middle of the culture period, and then reduced in the latter stages due to autoinhibition or repression by PoxClrB (Fig. 10). In contrast, *PoxClrB* expression gradually increased in the middle and later stages.

Moreover, PoxCxrA up-regulated the expression of genes involved in cellodextrin transportation, such as *POX06051*/*PoxCdtC* and *POX05915*/*PoxCdtD* [10] and retarded the expression of genes involved in glucose transportation such as *POX07576*/*GLT*-*1* and *POX08783*/*HGT*-*2* [12] that caused CCR at high concentration. PoxCxrA stimulated cellulase gene expression via two pathways; direct binding, and through key TF mediators such as PoxClrB (Fig. 10), PoxCxrB and/or PoxNsdD [8].

Differences in regulatory networks occurred in different host cells. In *N*. *crassa*, cellobiose-activated CLR1 was necessary for the increased expression of *clr2*, a homolog of *PoxClrB*. CLR1 also induced most cellulase genes, thereby positively affecting enzyme production [19]. In the present study, we found that PoxCxrA was required for *PoxClrB* expression, but not *POX03837*, which encodes a homolog of CLR1 in *P*. *oxalicum*. Knockout of *POX03837* had no effect on cellulase production in *P*. *oxalicum* HP7-1 cultured on Avicel (data not shown).

The PoxCxrA DBD resembles those of Gal4-like TFs (e.g. Gal4 in *S*. *cerevisiae*, CLR1 and XlnR in *N*. *crassa*, and PoxClrB). PoxCxrA_17–58_ was found to be the minimal functional DBD, and it comprises three pairs of zinc-coordinating cysteines and several conserved amino acids that are essential for protein binding [18]. The amino acids flanking these cysteines display high diversity, which might determine the binding motifs. In the literature, many binding motifs of Gal4-like proteins have been characterised, including CGGN5CGGNCCG (CLR1), CGGN11CCG (Gal4 or CLR2), GGNTAAA (XlnR) [9], TCG(G/C)(A/T)NNTTNAA (Pho7) [18] and 5′-ATCAGATCCTCAAAGA-3′ or 5′-GCTGAGTCCTT-3′ determined in this study. This suggests that the amino acids flanking the cysteines are also required for binding.

Moreover, screening PoxCxrA-binding sequences in other target genes confirmed by in vitro EMSA experiments using PBM_*POX05587* and PBM_*PoxClrB* revealed that the binding sequences were highly diverse, which supports the enormous regulon of PoxCxrA that includes 1970 members, accounting for almost a quarter of the entire *P*. *oxalicum* strain HP7-1 genome. However, identification and analysis of all PoxCxrA-binding sequences in the genome requires further study.

In summary, the present study determined regulon for the essential TF PoxCxrA that is required for cellulase production of *P*. *oxalicum*. PoxCxrA regulates cellulase gene expression via two mechanisms: regulating key TF mediator PoxClrB, and directly binding cellulase genes with diverse binding motifs. This work provides novel insights into the regulatory mechanisms of fungal cellulase gene expression.

## Methods

### Strains and culture conditions

*Penicillium oxalicum* mutant strains Δ*PoxCxrA* (no. 12965) and Δ*PoxClrB* (no. 3.15649), and the parental strain Δ*PoxKu70* (no. 3.15650) were obtained from the China General Microbiological Culture Collection (CGMCC) [8]. Spores were collected after maintaining strains on potato dextrose agar (PDA) plates at 28 °C for 6 days with buffer containing 0.1% Tween-80. In general, an aliquot of conidial suspension (1 × 10^8^ conidia per milliliter) was inoculated into 100 mL of modified culture medium (MMM; pH 5.5) containing (g/L) (NH_4_)_2_SO_4_ (4.0), KH_2_PO_4_ (4.0), CaCl_2_ (0.6), MgSO_4_·7H_2_O (0.60), FeSO_4_·7H_2_O (0.005), MnSO_4_ (0.0016), ZnCl_2_ (0.0017), CoCl_2_ (0.002), and 1 mL of Tween-80, containing either 1.0% glucose or 2.0% Avicel (Sigma-Aldrich, St. Louis, MO, USA) as the sole carbon source. Mixtures were incubated on an orbital shaker at 28 °C at 180 rpm for 6 days.

For transfer cultivation, *P*. *oxalicum* strains were first pre-cultured for 24 h on MMM supplemented with 1.0% glucose as the carbon source at 28 °C with shaking at 180 rpm. Mycelia of pre-cultured *P*. *oxalicum* strains were transferred into MMM containing 2.0% Avicel and cultured for 4–24 h at 28 °C with shaking at 180 rpm. Mycelia were harvested and total RNA was extracted for RNA-Seq and real-time quantitative reverse transcription-PCR (RT-qPCR) assays.

### RNA extraction

For total RNA extraction, mycelia harvested from three replicate independent cultures at each time point were frozen, ground under liquid nitrogen using a pestle and mortar, and RNA was purified using a TRIzol RNA Kit (Life Technologies, Carlsbad, CA, USA) according to the manufacturer’s instructions. RNA concentration and quality were determined from the ratio of the absorbance at 260 nm/280 nm measured using a Nanodrop ND-2000 spectrophotometer (ThermoFisher Scientific, Waltham, MA, USA).

### RNA-Seq

Total RNA was sequenced as described previously by Zhao et al. [2], and a cDNA library for each total RNA sample was constructed and assessed using an Agilent 2100 Bioanalyzer (Agilent Technologies, Santa Clara, CA, USA) and an ABI StepOnePlus Real-Time PCR System (Applied Biosystems, Foster City, CA, USA). After confirming eligibility, cDNA libraries were sequenced using an Illumina HiSeq 4000 system. After quality control, clean reads were mapped onto the genome of the wild-type *P*. *oxalicum* HP7-1 strain to assess sequence homology and functional annotation using BWA v0.7.10-r789 [23] and Bowtie2 v2.1.0 [24]. RSEM v1.2.12 software [25] was used to analyse gene expression levels using the fragments per kilobase of exon per million mapped reads (FPKM) method. Differentially expressed genes (DEGs) were screened using the DESeq tool [26] with |log2 fold change| ≥ 1 and *p* value ≤ 0.05 as thresholds. The reliability of RNA-Seq data was assessed by Pearson’s correlation coefficients for three biological replicates for each sample. BLAST v2.2.26 (http://blast.ncbi.nlm.nih.gov/Blast.cgi) was used for sequence homology and functional annotation analyses. DEGs detected by comparative assays were functionally analysed based on Eukaryotic Orthologous Group (KOG) annotation to the *P*. *oxalicum* HP7-1 genome [2].

### Expression and purification of truncated PoxCxrA constructs

Recombinant expression in *Escherichia coli* and protein purification were carried out as described previously by Yan et al. [8]. DNA sequences encoding a series of truncated PoxCxrA constructs (PoxCxrA_11–150_, PoxCxrA_11–114_, PoxCxrA_11–87_, PoxCxrA_11–58_, PoxCxrA_17–58_, PoxCxrA_11–50_ and PoxCxrA_11–31_) were amplified by PCR with specific primer pairs (Additional file 4: Table S3) and digested using appropriate restriction endonucleases. Digested DNA fragments were inserted into the expression vector pET32a (+) digested with the corresponding restriction endonucleases to generate recombinant plasmids that were subsequently introduced into competent *E. coli* cells by chemical recombination. After confirmation, cells harbouring constructs were pre-cultured for 12 h at 37 °C, then induced with 0.5 mM isopropyl-β-d-thiogalactopyranoside, with culturing being continued for 20 h at 28 °C to produce fusion proteins possessing thioredoxin (TRX), His and S tags. Fusion proteins were purified by affinity chromatography on TALON Metal Affinity Resin (Clontech, Palo Alto, CA, USA). Cells harbouring empty pET32a (+) vector were cultured as described above and the resulting Trx–His–S protein was used as a negative control.

### In vitro EMSA assays

In vitro EMSA experiments were performed as described previously by He et al. [17]. Briefly, DNA fragments of different lengths harbouring the putative promoter regions of *PoxClrB* and *POX05578*/*Cel7A*-*2*, labelled with 6-carboxyfluorescein (6-FAM) at the 3′ terminus, were amplified by PCR using specific primer pairs (Additional file 4: Table S3), and used as probes for in vitro EMSA experiments. Meanwhile, the same DNA fragments without 6-FAM and a 300-bp DNA fragment from the promoter region of the β-tubulin gene *POX05989* served as competitive and negative probes, respectively.

For EMSA experiments, mixtures containing ~ 50 ng EMSA probes and various amounts (0–2.0 µg) of fusion proteins in binding buffer consisting of 20 mM TRIS–HCl (pH 8.0), 0.1 mg/mL bovine serum albumin (BSA), 5% (v/v) glycerol, 50 mM KCl, 1 mM dithiothreitol and 1.0 µg sheared salmon sperm DNA) were cultured for 30 min at 30 °C, then separated by 4% polyacrylamide-TRIS–acetic acid-ethylene diamine tetraacetic acid (EDTA) gel electrophoresis. Gels were observed using a Bio-Rad ChemiDoc MP imaging system (BIO-RAD, Hercules, CA, USA) at an excitation wavelength range of 489–506 nm. Competitive binding experiments were performed as described above, except that probes were replaced with competitive probes.

### DNase I footprinting

DNase I footprinting experiments were carried out as reported by Wang et al. [27] with minor modifications. For each assay, 350 ng of each probe (100-bp DNA fragments corresponding to the region upstream from the start codon ATG of *POX05587*/*Cel7A*-*2*) were separately incubated with 6.6 µg of recombinant PoxCxrA_11–150_ protein for 20 min at 30 °C. Subsequently, 0.015 U DNase I (Promega, Beijing, China) and 100 nM CaCl_2_ were added and the reaction was incubated for 1 min at 30 °C. DNase I stop solution, containing 200 mM unbuffered sodium acetate, 30 mM EDTA and 0.15% sodium dodecyl sulphate (SDS), was used to stop the enzymatic reaction, and DNA was extracted and sequenced.

### RT-qPCR assays

RT-qPCR assays were carried out according to a previously reported method [8]. Briefly, total RNA was extracted from *P*. *oxalicum* deletion mutant ∆*PoxCxrA* grown on Avicel, and from the parental strain ∆*PoxKu70* that served as a control. First-strand cDNA was synthesized from the extracted RNA as template using a PrimeScript RT Reagent Kit (TaKaRa Bio Inc., Dalian, China) according to the manufacturer’s instructions. Each target gene was subjected to PCR amplification using the first-strand cDNA as template in a 20 µL reaction mixture containing 10 µM of each primer (0.8 µL; Additional file 4: Table S3), first-strand cDNA (0.2 µL) and SYBR Premix Ex Taq II (10 µL; TaKaRa Bio Inc.). Thermal cycling included 35 cycles at 95 °C for 3 s and 60 °C for 30 s. Fluorescent signals were measured at the end of each extension step at 80 °C. Transcriptional levels of each gene in deletion mutants ∆*PoxCxrA* and ∆*PoxClrB* were calculated relative to that of the control gene *POX09428* encoding actin, and relative expression levels were normalised against levels in the Δ*PoxKu70* strain. All RT-qPCR assays were performed independently in at least three replicates.

### Network construction

Network was constructed using Cytoscape v.3.6.1 software [28].

### Statistical analysis

Experimental data were statistically analysed by Student’s *t* tests using Microsoft Excel within Office 2016 (Microsoft, Redmond, WA, USA).

### Accession number

Transcriptomic data have been deposited in the Sequence Read Archive database under Accession Numbers SRR8377263–SRR8377265 for the ∆*PoxCxrA* and SRR8377258, SRR8377259 and SRR8377266.

## Additional files

**Additional file 1: Table S1.** Summary of transcriptomic data generated from *Penicillium oxalicum.*

**Additional file 2: Figure S1.** Pearson’s correlation analysis of transcriptomes from *Penicillium oxalicum* deletion mutant ∆*PoxCxrA* and the parental strain ∆*PoxKu70*. Total RNA was extracted from *P*. *oxalicum* strains cultivated in medium containing Avicel as the sole carbon source for 24 h after a shift from glucose, then sequenced.

**Additional file 3: Table S2.** *PoxCxrA* regulon in *Penicillium oxalicum* when subjected to Avicel as the sole carbon source.

**Additional file 4: Table S3.** Primers used in this study.

## Abbreviations

6-FAM: 6-carboxyfluorescein
BSA: bovine serum albumin
BGL: β-glucosidase
CAZymes: carbohydrate-active enzymes
CBH: cellobiohydrolase
CCR: carbon catabolite repression
CMCase: carboxymethylcellulase
CWDEs: plant cell wall-degrading enzymes
DBD: DNA-binding domain
DEGs: differentially expressed genes
EG: endo-β-1,4-glucanase
EMSA: electrophoretic mobility shift assay
FPase: filter paper cellulase
FPKM: fragments per kilobase of exon per million fragment mapped
MMM: modified culture medium
PBMs: protein-binding motifs
PDA: potato dextrose agar
pNPCase: *p*-nitrophenyl-β-cellobiosidase
pNPGase: *p*-nitrophenyl-β-glucopyranosidase
RT-qPCR: real-time quantitative reverse-transcription PCR
TFs: transcriptional factors
Xyn: xylanase
