# Supplementary material for: How an essential Zn2Cys6 transcription factor PoxCxrA regulates cellulase gene expression in ascomycete fungi?
Source: Biotechnol Biofuels. 2019 May 3;12:105. doi: 10.1186/s13068-019-1444-5 (PMC6498484; doi:10.1186/s13068-019-1444-5)
Supplement: Supplementary file 1 — Additional file 1: Table S1. Summary of transcriptomic data generated from Penicillium oxalicum. [file 13068_2019_1444_MOESM1_ESM.pdf]

Additional file 1: Table S1. Summary of transcriptomic data generated from *Penicillium oxalicum*

Samples[a]	Clean Reads	Number of nucleotides (bp)	Total mapping radio (%)	Uniquely mapping radio (%)	Number of expressed genes
Δ*PoxKu70*_24h-1	22756334	2275633400	92.62	86.89	8520
Δ*PoxKu70*_24h-2	21941480	2194148000	91.80	87.26	8320
Δ*PoxKu70*_24h-3	21955336	2195533600	90.47	86.28	8400
Δ*PoxCxrA*_24h-1	22050054	2205005400	92.05	86.78	8571
Δ*PoxCxrA*_24h-2	21835528	2183552800	91.03	86.88	8474
Δ*PoxCxrA*_24h-3	21329738	2132973800	91.34	87.01	8514
