# Supplementary figures and images for: How an essential Zn2Cys6 transcription factor PoxCxrA regulates cellulase gene expression in ascomycete fungi?
Source: Biotechnol Biofuels. 2019 May 3;12:105. doi: 10.1186/s13068-019-1444-5 (PMC6498484; doi:10.1186/s13068-019-1444-5)

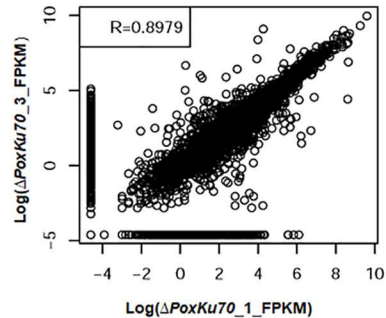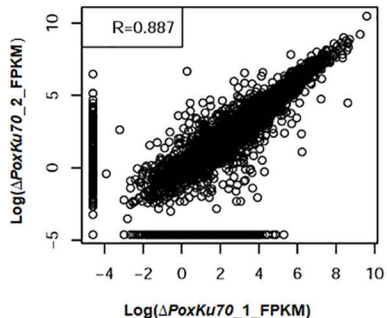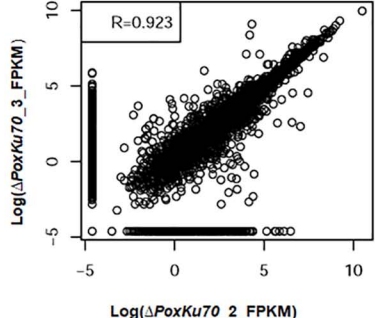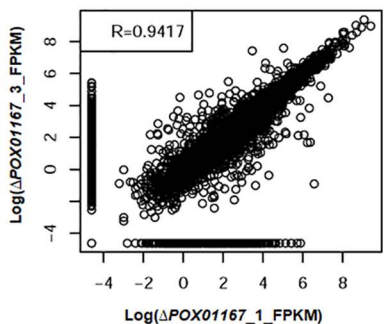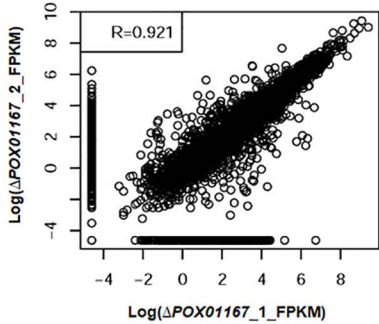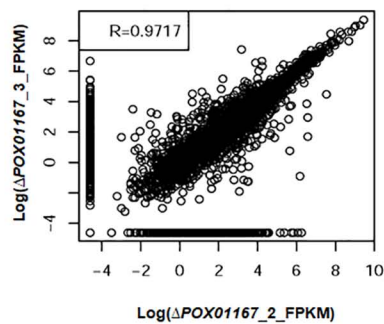

Supplement: Supplementary file 2 — Additional file 2: Figure S1. Pearson’s correlation analysis of transcriptomes from Penicillium oxalicum deletion mutant ∆PoxCxrA and the parental strain ∆PoxKu70. Total RNA was extracted from P. oxalicum strains cultivated in medium containing Avicel as the sole carbon source for 24 h after a shift from glucose, then sequenced. [file 13068_2019_1444_MOESM2_ESM.pdf]
