# Supplementary material for: How an essential Zn2Cys6 transcription factor PoxCxrA regulates cellulase gene expression in ascomycete fungi?
Source: Biotechnol Biofuels. 2019 May 3;12:105. doi: 10.1186/s13068-019-1444-5 (PMC6498484; doi:10.1186/s13068-019-1444-5)
Supplement: Supplementary file 3 — Additional file 3: Table S2. PoxCxrA regulon in Penicillium oxalicum when subjected to Avicel as the sole carbon source. [file 13068_2019_1444_MOESM3_ESM.pdf]

Additional file 3: Table S2 *PoxCxrA* regulon in *Penicillium oxalicum* subjected to Avicel as the sole carbon source.

Gene ID	Functional annotation	CAZy family	Log2 (∆*PoxCxrA* _24h/∆*PoxKu70* _24h)	*P* -value	Up/Down-Regulation
POX00003	Hypothetical protein	NA	-1.36	0.00	Down
POX00004	Hypothetical protein	NA	-1.45	0.00	Down
POX00005	Major facilitator, sugar transporter-like	NA	-1.60	0.00	Down
POX00007	Putative beta-xylosidase	GH3;AA5	-1.57	0.00	Down
POX00008	Putative exo-alpha-L-1,5-arabinanase	GH33;GH93	-1.59	0.00	Down
POX00009	Hypothetical protein	NA	-1.65	0.00	Down
POX00011	Hypothetical protein	NA	2.61	0.00	Up
POX00024	Hypothetical protein	NA	1.14	0.00	Up
POX00029	Hypothetical protein	NA	1.07	0.00	Up
POX00032	Hypothetical protein	NA	-1.34	0.00	Down
POX00036	Hypothetical protein	NA	-1.05	0.00	Down
POX00039	Hypothetical protein	NA	-3.45	0.00	Down
POX00040	Hypothetical protein	NA	-3.28	0.00	Down
POX00041	Hypothetical protein	NA	-2.50	0.00	Down
POX00042	Hypothetical protein	NA	-1.70	0.00	Down
POX00044	Hypothetical protein	NA	-2.98	0.00	Down
POX00054	Hypothetical protein	NA	-1.28	0.00	Down
POX00063	Endo-beta-1,4-xylanase	CBM1;GH10	-1.89	0.00	Down
POX00068	Hypothetical protein	NA	2.31	0.00	Up
POX00072	Hypothetical protein	NA	-1.63	0.00	Down
POX00089	Putative chitinase	GH18	-4.77	0.00	Down
POX00092	Hypothetical protein	NA	-1.75	0.00	Down
POX00093	Hypothetical protein	NA	-2.26	0.00	Down
POX00094	Hypothetical protein	NA	-1.58	0.00	Down
POX00096	Hypothetical protein	NA	-2.37	0.00	Down
POX00099	Hypothetical protein	NA	1.03	0.00	Up
POX00110	Hypothetical protein	NA	1.13	0.00	Up
POX00111	Hypothetical protein	NA	-1.92	0.00	Down
POX00120	Hypothetical protein	NA	-3.15	0.00	Down
POX00129	Hypothetical protein	NA	-1.17	0.00	Down
POX00130	Winged helix repressor DNA-binding	NA	1.25	0.00	Up
POX00142	Hypothetical protein	NA	-3.27	0.00	Down
POX00144	Hypothetical protein	AA7	-1.45	0.00	Down
POX00148	Zinc finger, C2H2-type	NA	-1.48	0.00	Down
POX00149	Hypothetical protein	NA	1.14	0.00	Up
POX00150	Hypothetical protein	NA	1.48	0.00	Up

POX00151	Hypothetical protein	NA	1.08	0.00	Up
POX00161	Hypothetical protein	NA	-1.33	0.00	Down
POX00169	Hypothetical protein	NA	-1.46	0.00	Down
POX00174	Hypothetical protein	NA	-1.99	0.00	Down
POX00176	Hypothetical protein	NA	-1.29	0.00	Down
POX00179	Hypothetical protein	NA	1.07	0.00	Up
POX00183	Hypothetical protein	NA	2.08	0.00	Up
POX00189	Hypothetical protein	NA	-1.39	0.00	Down
POX00223	Hypothetical protein	NA	1.01	0.00	Up
POX00227	Hypothetical protein	NA	2.53	0.00	Up
POX00232	Hypothetical protein	NA	-1.58	0.00	Down
POX00234	Hypothetical protein	NA	-1.20	0.00	Down
POX00235	Hypothetical protein	NA	-1.44	0.00	Down
POX00236	Hypothetical protein	NA	-2.33	0.05	Down
POX00243	Hypothetical protein	NA	-5.28	0.00	Down
POX00244	Hypothetical protein	NA	-4.48	0.00	Down
POX00246	Hypothetical protein	NA	-3.33	0.00	Down
POX00252	Hypothetical protein	NA	2.16	0.00	Up
POX00255	Hypothetical protein	NA	-1.15	0.00	Down
POX00260	Hypothetical protein	NA	-2.98	0.00	Down
POX00271	Hypothetical protein	NA	-1.06	0.00	Down
POX00284	Hypothetical protein	NA	2.81	0.00	Up
POX00289	Hypothetical protein	NA	1.89	0.00	Up
POX00308	Hypothetical protein	NA	-4.42	0.00	Down
POX00328	Hypothetical protein	NA	1.09	0.00	Up
POX00331	Zinc finger, C2H2-type	NA	-2.33	0.00	Down
POX00338	Hypothetical protein	NA	-3.51	0.00	Down
POX00343	Hypothetical protein	NA	2.47	0.04	Up
POX00347	Hypothetical protein	CBM21	-1.25	0.00	Down
POX00351	Hypothetical protein	NA	2.33	0.00	Up
POX00352	Hypothetical protein	NA	1.32	0.00	Up
POX00353	Hypothetical protein	NA	-2.26	0.00	Down
POX00355	Hypothetical protein	NA	-1.09	0.00	Down
POX00372	Hypothetical protein	NA	-1.19	0.00	Down
POX00375	Hypothetical protein	NA	3.24	0.00	Up
POX00376	Hypothetical protein	NA	1.70	0.00	Up
POX00379	Hypothetical protein	NA	1.91	0.00	Up
POX00381	Hypothetical protein	NA	1.10	0.00	Up
POX00395	Hypothetical protein	NA	1.93	0.00	Up
POX00401	Hypothetical protein	NA	-1.11	0.00	Down

POX00403	Hypothetical protein	NA	1.39	0.00	Up
POX00416	Hypothetical protein	NA	1.15	0.00	Up
POX00427	Hypothetical protein	NA	-1.17	0.00	Down
POX00429	Hypothetical protein	NA	-1.31	0.00	Down
POX00435	Hypothetical protein	NA	1.13	0.00	Up
POX00436	Hypothetical protein	NA	1.17	0.00	Up
POX00438	Hypothetical protein	NA	-1.11	0.00	Down
POX00449	Hypothetical protein	NA	-1.08	0.00	Down
POX00452	Hypothetical protein	NA	2.01	0.00	Up
POX00455	Hypothetical protein	NA	4.28	0.00	Up
POX00464	High mobility group box	NA	-2.53	0.00	Down
POX00471	Hypothetical protein	NA	1.53	0.00	Up
POX00497	Hypothetical protein	NA	-1.27	0.00	Down
POX00498	Hypothetical protein	NA	1.34	0.00	Up
POX00502	Hypothetical protein	NA	4.62	0.00	Up
POX00503	Hypothetical protein	NA	1.95	0.00	Up
POX00522	Hypothetical protein	NA	2.01	0.00	Up
POX00533	Hypothetical protein	NA	2.37	0.04	Up
POX00535	Hypothetical protein	NA	1.19	0.00	Up
POX00541	Hypothetical protein	NA	3.11	0.01	Up
POX00553	Hypothetical protein	NA	-1.84	0.00	Down
POX00555	Hypothetical protein	NA	3.01	0.00	Up
POX00556	Hypothetical protein	NA	2.62	0.00	Up
POX00558	Hypothetical protein	NA	-3.09	0.00	Down
POX00561	Hypothetical protein	NA	2.70	0.00	Up
POX00562	Hypothetical protein	NA	2.49	0.00	Up
POX00571	Hypothetical protein	NA	-1.75	0.00	Down
POX00579	Hypothetical protein	NA	-1.03	0.00	Down
POX00592	Hypothetical protein	NA	1.59	0.00	Up
POX00593	Hypothetical protein	NA	2.00	0.00	Up
POX00610	Hypothetical protein	NA	-1.21	0.00	Down
POX00618	Hypothetical protein	NA	-1.81	0.00	Down
POX00619	Putative chitin synthase	GT2	-1.06	0.00	Down
POX00634	Hypothetical protein	NA	3.09	0.00	Up
POX00639	Hypothetical protein	NA	-2.42	0.05	Down
POX00641	Hypothetical protein	NA	1.29	0.00	Up
POX00642	Hypothetical protein	NA	-1.51	0.00	Down
POX00652	Hypothetical protein	NA	1.18	0.00	Up
POX00654	Hypothetical protein	NA	3.50	0.00	Up
POX00656	Hypothetical protein	NA	-1.14	0.00	Down

POX00665	Hypothetical protein	NA	1.44	0.00	Up
POX00692	Hypothetical protein	NA	4.13	0.00	Up
POX00693	Hypothetical protein	NA	2.03	0.00	Up
POX00701	Hypothetical protein	NA	-1.01	0.00	Down
POX00702	Hypothetical protein	NA	2.82	0.00	Up
POX00713	Hypothetical protein	NA	-2.44	0.00	Down
POX00714	Hypothetical protein	NA	-1.54	0.00	Down
POX00715	Hypothetical protein	NA	-1.70	0.00	Down
POX00718	Hypothetical protein	NA	3.92	0.00	Up
POX00719	Hypothetical protein	NA	-1.55	0.00	Down
POX00729	Hypothetical protein	NA	2.20	0.00	Up
POX00742	Hypothetical protein	NA	3.72	0.00	Up
POX00756	Hypothetical protein	NA	3.68	0.00	Up
POX00760	Putative pectin methylesterase	CE8	-1.98	0.00	Down
POX00761	Hypothetical protein	NA	1.87	0.00	Up
POX00769	Hypothetical protein	NA	1.13	0.00	Up
POX00772	Hypothetical protein	NA	2.26	0.00	Up
POX00777	Hypothetical protein	NA	2.82	0.00	Up
POX00781	Hypothetical protein	NA	1.06	0.00	Up
POX00783	Hypothetical protein	NA	1.35	0.00	Up
POX00803	Hypothetical protein	NA	-1.01	0.04	Down
POX00804	Hypothetical protein	NA	-1.62	0.00	Down
POX00836	Hypothetical protein	NA	-1.45	0.00	Down
POX00837	Hypothetical protein	NA	-1.53	0.00	Down
POX00848	Hypothetical protein	NA	1.29	0.00	Up
POX00849	Hypothetical protein	NA	4.25	0.00	Up
POX00850	Hypothetical protein	NA	1.64	0.00	Up
POX00860	Hypothetical protein	NA	1.86	0.00	Up
POX00861	Hypothetical protein	NA	4.24	0.00	Up
POX00862	Hypothetical protein	NA	3.98	0.00	Up
POX00863	Hypothetical protein	NA	3.44	0.00	Up
POX00864	Zn2Cys6; Fungal transcriptional regulatory protein, N-terminal	NA	1.22	0.00	Up
POX00868	Hypothetical protein	NA	1.52	0.00	Up
POX00870	Hypothetical protein	NA	1.64	0.00	Up
POX00873	Hypothetical protein	NA	2.05	0.00	Up
POX00878	Hypothetical protein	NA	-1.10	0.00	Down
POX00880	Putative GDP-Man: alpha-1, 3-mannosyltransferase	GT69	1.20	0.04	Up
POX00883	Hypothetical protein	NA	1.55	0.00	Up
POX00884	Hypothetical protein	NA	1.25	0.00	Up
POX00919	Hypothetical protein	NA	-1.71	0.00	Down

POX00923	Putative alpha-L-arabinofuranosidase	GH43	-4.53	0.00	Down
POX00925	Hypothetical protein	NA	-2.96	0.00	Down
POX00927	Hypothetical protein	NA	1.09	0.00	Up
POX00936	Zn2Cys6; Fungal transcriptional regulatory protein, N-terminal	NA	-2.23	0.00	Down
POX00951	Hypothetical protein	NA	-1.10	0.00	Down
POX00954	Hypothetical protein	NA	-1.09	0.00	Down
POX00959	Hypothetical protein	NA	-2.81	0.00	Down
POX00961	Hypothetical protein	NA	-2.25	0.00	Down
POX00966	Hypothetical protein	NA	-2.67	0.00	Down
POX00968	Putative beta-glucosidase	GH3	1.80	0.00	Up
POX00970	Putative alpha-1, 6-mannosyltransferase	GT32	3.50	0.00	Up
POX00975	Zn2Cys6; Fungal transcriptional regulatory protein, N-terminal	NA	2.16	0.00	Up
POX00983	Hypothetical protein	NA	1.13	0.00	Up
POX00984	Hypothetical protein	NA	1.43	0.00	Up
POX00989	Hypothetical protein	NA	-3.55	0.00	Down
POX00997	Hypothetical protein	NA	1.38	0.00	Up
POX01001	Hypothetical protein	NA	-1.11	0.00	Down
POX01002	Hypothetical protein	NA	1.13	0.00	Up
POX01009	Hypothetical protein	NA	3.31	0.00	Up
POX01024	Hypothetical protein	NA	-1.03	0.00	Down
POX01031	Hypothetical protein	NA	-1.49	0.00	Down
POX01044	Hypothetical protein	NA	-2.47	0.00	Down
POX01054	Hypothetical protein	NA	-1.01	0.00	Down
POX01055	Hypothetical protein	NA	-1.15	0.01	Down
POX01057	Hypothetical protein	NA	2.70	0.00	Up
POX01060	Hypothetical protein	NA	3.72	0.00	Up
POX01067	Hypothetical protein	NA	1.10	0.00	Up
POX01079	Hypothetical protein	NA	2.56	0.00	Up
POX01081	Hypothetical protein	NA	1.21	0.00	Up
POX01083	Hypothetical protein	NA	1.08	0.00	Up
POX01087	Hypothetical protein	NA	-1.16	0.01	Down
POX01090	Hypothetical protein	NA	-1.03	0.00	Down
POX01092	Hypothetical protein	NA	1.27	0.00	Up
POX01103	Hypothetical protein	NA	1.35	0.00	Up
POX01105	Hypothetical protein	NA	-2.77	0.00	Down
POX01117	Hypothetical protein	NA	-1.52	0.00	Down
POX01118	Zn2Cys6; Fungal transcriptional regulatory protein, N-terminal	NA	-1.13	0.00	Down
POX01121	Hypothetical protein	NA	-1.54	0.03	Down
POX01126	Hypothetical protein	NA	-1.14	0.00	Down
POX01136	Zn2Cys6; Fungal transcriptional regulatory protein, N-terminal	NA	1.46	0.04	Up

POX01138	Hypothetical protein	NA	1.12	0.00	Up
POX01139	Hypothetical protein	NA	4.74	0.00	Up
POX01143	Hypothetical protein	NA	-1.28	0.00	Down
POX01150	Hypothetical protein	NA	-5.03	0.00	Down
POX01152	Hypothetical protein	AA7	8.43	0.00	Up
POX01153	Hypothetical protein	NA	1.98	0.00	Up
POX01158	SUN domain-containing protein	GH132	-1.11	0.00	Down
POX01167	Zn2Cys6; Fungal transcriptional regulatory protein, N-terminal	NA	-8.05	0.00	Down
POX01168	Hypothetical protein	NA	-5.20	0.00	Down
POX01170	Hypothetical protein	NA	-1.16	0.00	Down
POX01172	Hypothetical protein	NA	-1.05	0.00	Down
POX01173	Hypothetical protein	NA	-1.21	0.00	Down
POX01175	Hypothetical protein	NA	-3.92	0.00	Down
POX01177	Hypothetical protein	NA	-1.15	0.00	Down
POX01178	Hypothetical protein	NA	-1.06	0.00	Down
POX01179	Hypothetical protein	NA	-1.13	0.00	Down
POX01182	Hypothetical protein	NA	-1.46	0.00	Down
POX01184	Winged helix repressor DNA-binding	NA	-2.66	0.00	Down
POX01185	Hypothetical protein	NA	-1.72	0.00	Down
POX01188	Hypothetical protein	NA	-2.47	0.00	Down
POX01192	Hypothetical protein	GT34	-3.02	0.00	Down
POX01193	Hypothetical protein	NA	-4.88	0.00	Down
POX01203	Hypothetical protein	NA	1.13	0.00	Up
POX01209	Hypothetical protein	NA	1.59	0.02	Up
POX01210	Hypothetical protein	NA	2.87	0.00	Up
POX01214	Hypothetical protein	NA	2.64	0.00	Up
POX01215	Hypothetical protein	NA	5.19	0.00	Up
POX01217	Hypothetical protein	NA	1.22	0.00	Up
POX01218	Putative acetyl xylan esterase	CBM1;CE1	-1.52	0.00	Down
POX01224	Hypothetical protein	NA	-1.30	0.00	Down
POX01228	Hypothetical protein	NA	1.08	0.00	Up
POX01231	Hypothetical protein	NA	1.52	0.01	Up
POX01234	Hypothetical protein	NA	3.39	0.00	Up
POX01235	Hypothetical protein	NA	-1.06	0.00	Down
POX01249	Zn2Cys6; Fungal transcriptional regulatory protein, N-terminal	NA	2.05	0.00	Up
POX01254	Hypothetical protein	NA	4.14	0.00	Up
POX01262	Hypothetical protein	NA	-2.75	0.00	Down
POX01263	Hypothetical protein	NA	-1.33	0.00	Down
POX01273	Hypothetical protein	NA	1.07	0.00	Up
POX01286	Hypothetical protein	NA	-1.06	0.00	Down

POX01287	Hypothetical protein	NA	-1.04	0.00	Down
POX01289	Hypothetical protein	NA	1.62	0.00	Up
POX01290	Hypothetical protein	NA	1.44	0.00	Up
POX01296	Zinc finger, C2H2-type	NA	-2.53	0.00	Down
POX01302	Hypothetical protein	NA	-1.41	0.00	Down
POX01307	Hypothetical protein	NA	-1.22	0.04	Down
POX01318	Hypothetical protein	NA	-2.07	0.00	Down
POX01320	Hypothetical protein	NA	2.29	0.00	Up
POX01333	Hypothetical protein	NA	1.71	0.00	Up
POX01343	Hypothetical protein	NA	1.36	0.00	Up
POX01346	putative chitin synthase	NA	3.29	0.00	Up
POX01347	Hypothetical protein	NA	2.06	0.03	Up
POX01356	Glucoamylase Amy15A	CBM20;GH15	1.41	0.00	Up
POX01360	Hypothetical protein	NA	2.67	0.03	Up
POX01362	Hypothetical protein	NA	2.39	0.00	Up
POX01367	Hypothetical protein	NA	3.70	0.00	Up
POX01369	Hypothetical protein	GT41	-2.97	0.00	Down
POX01371	Hypothetical protein	NA	1.05	0.00	Up
POX01377	Zn2Cys6; Fungal specific transcription factor	NA	1.04	0.00	Up
POX01379	Hypothetical protein	NA	2.63	0.00	Up
POX01381	Chitin binding domain-containing protein	AA11	3.90	0.00	Up
POX01384	Hypothetical protein	NA	3.32	0.00	Up
POX01387	Zn2Cys6; Fungal transcriptional regulatory protein, N-terminal	NA	1.39	0.00	Up
POX01390	Hypothetical protein	CE1	-6.80	0.00	Down
POX01391	Hypothetical protein	AA1	-3.30	0.00	Down
POX01394	Hypothetical protein	NA	-1.30	0.00	Down
POX01400	Hypothetical protein	NA	4.89	0.00	Up
POX01413	Hypothetical protein	NA	-4.38	0.00	Down
POX01415	Hypothetical protein	NA	1.58	0.00	Up
POX01423	Hypothetical protein	NA	1.34	0.00	Up
POX01430	Hypothetical protein	NA	-3.57	0.00	Down
POX01431	Hypothetical protein	AA1	-7.02	0.00	Down
POX01439	Hypothetical protein	NA	-1.19	0.00	Down
POX01448	Hypothetical protein	NA	1.01	0.00	Up
POX01460	Hypothetical protein	NA	-2.69	0.00	Down
POX01462	Hypothetical protein	NA	-1.65	0.00	Down
POX01470	Putative chitinase	GH18	1.15	0.00	Up
POX01476	Hypothetical protein	NA	-1.95	0.00	Down
POX01487	Hypothetical protein	NA	1.19	0.00	Up
POX01489	Hypothetical protein	NA	-4.86	0.00	Down

POX01501	Hypothetical protein	NA	2.04	0.00	Up
POX01507	Hypothetical protein	NA	-5.56	0.00	Down
POX01509	Hypothetical protein	NA	1.23	0.00	Up
POX01513	Hypothetical protein	NA	-1.42	0.00	Down
POX01519	Hypothetical protein	NA	-4.55	0.00	Down
POX01531	Hypothetical protein	NA	1.56	0.00	Up
POX01536	Hypothetical protein	NA	-4.82	0.00	Down
POX01539	Hypothetical protein	NA	1.59	0.00	Up
POX01543	Hypothetical protein	NA	-1.89	0.00	Down
POX01546	Hypothetical protein	NA	-1.54	0.00	Down
POX01554	Hypothetical protein	NA	-1.84	0.00	Down
POX01562	Hypothetical protein	NA	2.80	0.02	Up
POX01566	Sugar/inositol transporter	NA	-3.37	0.00	Down
POX01576	Hypothetical protein	NA	-1.04	0.00	Down
POX01577	Hypothetical protein	NA	-1.73	0.01	Down
POX01578	Hypothetical protein	NA	-1.04	0.00	Down
POX01599	Hypothetical protein	NA	1.90	0.00	Up
POX01600	Hypothetical protein	NA	1.28	0.00	Up
POX01602	Hypothetical protein	NA	2.08	0.00	Up
POX01610	Hypothetical protein	NA	1.68	0.00	Up
POX01611	Hypothetical protein	NA	1.29	0.00	Up
POX01615	Putative GDP-Man: alpha-1, 3-mannosyltransferase	GT69	-2.92	0.00	Down
POX01617	Hypothetical protein	NA	1.60	0.00	Up
POX01618	Hypothetical protein	NA	5.04	0.00	Up
POX01619	Hypothetical protein	NA	1.36	0.00	Up
POX01622	Hypothetical protein	NA	4.79	0.00	Up
POX01635	Hypothetical protein	NA	-1.40	0.00	Down
POX01636	Hypothetical protein	NA	-2.11	0.00	Down
POX01637	Hypothetical protein	NA	-2.41	0.00	Down
POX01646	Putative beta-xylosidase	GH3	-1.67	0.00	Down
POX01651	Hypothetical protein	NA	-8.39	0.00	Down
POX01653	Hypothetical protein	NA	2.89	0.00	Up
POX01658	Hypothetical protein	NA	-1.11	0.00	Down
POX01680	Hypothetical protein	NA	2.42	0.00	Up
POX01682	Hypothetical protein	NA	-3.96	0.00	Down
POX01683	Hypothetical protein	NA	3.28	0.00	Up
POX01684	Hypothetical protein	NA	3.73	0.00	Up
POX01690	Hypothetical protein	NA	1.14	0.00	Up
POX01691	Hypothetical protein	NA	1.35	0.00	Up
POX01692	Hypothetical protein	NA	1.51	0.00	Up

POX01693	Hypothetical protein	NA	1.45	0.00	Up
POX01695	Hypothetical protein	NA	2.13	0.00	Up
POX01701	Hypothetical protein	CE9	5.37	0.00	Up
POX01702	Hypothetical protein	NA	1.19	0.00	Up
POX01704	Hypothetical protein	NA	-2.99	0.00	Down
POX01709	Hypothetical protein	NA	-1.17	0.00	Down
POX01713	Hypothetical protein	NA	-2.17	0.00	Down
POX01719	Hypothetical protein	NA	1.59	0.04	Up
POX01725	Zinc finger, DHHC-type	NA	2.71	0.00	Up
POX01726	Hypothetical protein	NA	7.95	0.00	Up
POX01730	Hypothetical protein	NA	3.61	0.00	Up
POX01735	Hypothetical protein	NA	1.06	0.00	Up
POX01741	Hypothetical protein	NA	1.22	0.00	Up
POX01744	Putative alpha-L-arabinofuranosidase	GH117;GH43	2.84	0.00	Up
POX01745	Putative alpha-1,6-mannosyltransferase	GT32	1.07	0.00	Up
POX01750	Hypothetical protein	NA	-1.02	0.00	Down
POX01762	Hypothetical protein	NA	-4.77	0.00	Down
POX01763	Hypothetical protein	NA	-2.52	0.00	Down
POX01764	Hypothetical protein	NA	-8.72	0.00	Down
POX01774	Hypothetical protein	NA	1.24	0.00	Up
POX01777	Hypothetical protein	NA	-2.07	0.00	Down
POX01780	Hypothetical protein	NA	1.24	0.00	Up
POX01783	Hypothetical protein	NA	1.01	0.01	Up
POX01797	Hypothetical protein	NA	-1.16	0.00	Down
POX01803	Hypothetical protein	NA	-4.59	0.00	Down
POX01804	Hypothetical protein	NA	-4.63	0.00	Down
POX01805	Hypothetical protein	NA	-2.28	0.00	Down
POX01807	Hypothetical protein	NA	-1.20	0.00	Down
POX01810	Hypothetical protein	NA	1.29	0.00	Up
POX01815	Hypothetical protein	NA	1.33	0.00	Up
POX01833	Putative alpha-mannosyltransferase	GT71	1.71	0.00	Up
POX01842	Hypothetical protein	NA	-6.80	0.00	Down
POX01843	Hypothetical protein	NA	-3.02	0.00	Down
POX01844	Hypothetical protein	NA	2.28	0.00	Up
POX01846	Hypothetical protein	NA	1.39	0.00	Up
POX01847	Hypothetical protein	NA	2.55	0.00	Up
POX01848	Hypothetical protein	NA	1.22	0.00	Up
POX01860	Hypothetical protein	NA	1.48	0.00	Up
POX01861	Hypothetical protein	NA	1.38	0.00	Up
POX01862	Hypothetical protein	NA	1.37	0.00	Up

POX01864	Hypothetical protein	NA	3.63	0.00	Up
POX01867	Hypothetical protein	NA	1.69	0.00	Up
POX01886	Hypothetical protein	NA	3.30	0.00	Up
POX01904	Hypothetical protein	NA	-1.01	0.00	Down
POX01910	Hypothetical protein	NA	1.03	0.00	Up
POX01912	Hypothetical protein	NA	2.10	0.01	Up
POX01913	Hypothetical protein	NA	2.71	0.00	Up
POX01916	Hypothetical protein	NA	-1.13	0.00	Down
POX01918	Hypothetical protein	NA	-1.30	0.00	Down
POX01919	Hypothetical protein	NA	2.58	0.01	Up
POX01920	Sugar/inositol transporter	NA	-1.34	0.00	Down
POX01922	Hypothetical protein	NA	1.65	0.00	Up
POX01925	Hypothetical protein	NA	-2.25	0.00	Down
POX01932	Hypothetical protein	NA	1.38	0.00	Up
POX01934	Putative chitosanase	GH75	-2.18	0.01	Down
POX01935	Sugar/inositol transporter	NA	1.47	0.00	Up
POX01937	Mannanase	CBM1;GH5	-1.44	0.00	Down
POX01940	Hypothetical protein	NA	-1.63	0.00	Down
POX01945	Hypothetical protein	AA7	3.97	0.00	Up
POX01949	Hypothetical protein	NA	-1.56	0.00	Down
POX01950	Hypothetical protein	NA	1.84	0.00	Up
POX01957	Zinc finger, Rad18-type putative	NA	1.80	0.00	Up
POX01960	Zn2Cys6; Fungal transcriptional regulatory protein, N-terminal	NA	-1.15	0.00	Down
POX01979	Hypothetical protein	NA	2.13	0.00	Up
POX01980	Hypothetical protein	NA	-1.52	0.00	Down
POX01981	Hypothetical protein	NA	1.57	0.00	Up
POX01984	Hypothetical protein	NA	1.58	0.00	Up
POX01987	Hypothetical protein	NA	-2.16	0.00	Down
POX01995	Hypothetical protein	NA	1.24	0.00	Up
POX02002	Hypothetical protein	NA	3.36	0.00	Up
POX02004	Hypothetical protein	NA	1.51	0.00	Up
POX02005	Hypothetical protein	NA	1.34	0.00	Up
POX02006	Hypothetical protein	NA	1.76	0.00	Up
POX02007	Hypothetical protein	NA	1.35	0.03	Up
POX02008	Hypothetical protein	NA	-3.33	0.00	Down
POX02011	Hypothetical protein	NA	-1.01	0.00	Down
POX02016	Hypothetical protein	NA	-1.78	0.00	Down
POX02017	Hypothetical protein	NA	2.99	0.01	Up
POX02021	Hypothetical protein	NA	1.13	0.00	Up
POX02024	Putative rhamnogalacturonase	GH28	-1.68	0.00	Down

POX02026	Hypothetical protein	NA	2.20	0.00	Up
POX02029	Zn2Cys6; Fungal transcriptional regulatory protein, N-terminal	NA	2.86	0.00	Up
POX02034	Hypothetical protein	GT41	1.07	0.00	Up
POX02035	Winged helix repressor DNA-binding	NA	1.21	0.00	Up
POX02038	Hypothetical protein	NA	1.42	0.00	Up
POX02044	Hypothetical protein	NA	1.19	0.00	Up
POX02045	Hypothetical protein	NA	1.10	0.00	Up
POX02048	Hypothetical protein	NA	5.62	0.00	Up
POX02049	Hypothetical protein	NA	1.01	0.00	Up
POX02054	Hypothetical protein	NA	-1.18	0.00	Down
POX02055	Hypothetical protein	NA	-2.12	0.00	Down
POX02057	Hypothetical protein	NA	-1.50	0.00	Down
POX02063	Hypothetical protein	NA	4.40	0.00	Up
POX02072	Hypothetical protein	NA	-1.88	0.00	Down
POX02073	Hypothetical protein	NA	-1.80	0.00	Down
POX02081	Hypothetical protein	NA	1.49	0.00	Up
POX02111	Hypothetical protein	NA	-2.70	0.00	Down
POX02131	Hypothetical protein	NA	1.74	0.00	Up
POX02133	Hypothetical protein	NA	-1.33	0.00	Down
POX02136	ABC drug exporter AtrF	NA	-1.93	0.00	Down
POX02151	Hypothetical protein	NA	1.84	0.00	Up
POX02157	Hypothetical protein	NA	-1.07	0.00	Down
POX02188	Hypothetical protein	NA	-1.86	0.00	Down
POX02191	Hypothetical protein	NA	2.12	0.04	Up
POX02209	Hypothetical protein	NA	2.94	0.00	Up
POX02214	Hypothetical protein	NA	1.42	0.00	Up
POX02215	Hypothetical protein	NA	2.45	0.00	Up
POX02216	Hypothetical protein	NA	-1.02	0.02	Down
POX02219	Hypothetical protein	NA	-1.18	0.00	Down
POX02221	Hypothetical protein	NA	-1.52	0.02	Down
POX02244	Hypothetical protein	NA	1.45	0.00	Up
POX02253	Hypothetical protein	NA	1.15	0.00	Up
POX02260	Hypothetical protein	NA	2.06	0.00	Up
POX02269	Hypothetical protein	NA	1.88	0.00	Up
POX02274	Hypothetical protein	NA	1.64	0.00	Up
POX02276	Hypothetical protein	NA	1.30	0.04	Up
POX02277	Hypothetical protein	NA	4.35	0.00	Up
POX02282	Hypothetical protein	NA	-1.92	0.02	Down
POX02285	Hypothetical protein	NA	2.50	0.00	Up
POX02287	Hypothetical protein	NA	-1.02	0.00	Down

POX02288	Hypothetical protein	NA	1.03	0.01	Up
POX02291	Hypothetical protein	NA	-1.31	0.00	Down
POX02306	Hypothetical protein	NA	-1.21	0.00	Down
POX02308	Cellulose monooxygenase Cel61A	AA9	-2.42	0.00	Down
POX02316	Hypothetical protein	NA	1.08	0.00	Up
POX02317	Hypothetical protein	NA	-1.10	0.00	Down
POX02319	Hypothetical protein	NA	1.91	0.00	Up
POX02321	Hypothetical protein	NA	3.98	0.00	Up
POX02326	Hypothetical protein	NA	1.55	0.00	Up
POX02337	Hypothetical protein	NA	2.98	0.00	Up
POX02338	Basic-leucine zipper (bZIP) transcription factor	NA	2.06	0.01	Up
POX02339	Hypothetical protein	NA	1.04	0.00	Up
POX02352	Hypothetical protein	NA	-2.06	0.00	Down
POX02353	Hypothetical protein	AA7	-1.22	0.00	Down
POX02355	Hypothetical protein	NA	1.18	0.00	Up
POX02360	Hypothetical protein	NA	-2.06	0.00	Down
POX02361	Hypothetical protein	NA	-1.91	0.00	Down
POX02365	Hypothetical protein	NA	-1.94	0.00	Down
POX02366	Hypothetical protein	NA	-3.16	0.00	Down
POX02371	Hypothetical protein	NA	3.28	0.00	Up
POX02378	Hypothetical protein	NA	2.22	0.01	Up
POX02393	Hypothetical protein	NA	-1.01	0.00	Down
POX02394	Basic-leucine zipper (bZIP) transcription factor	NA	-3.46	0.00	Down
POX02396	Hypothetical protein	NA	-1.49	0.00	Down
POX02402	Hypothetical protein	NA	1.92	0.00	Up
POX02407	Hypothetical protein	NA	1.37	0.00	Up
POX02408	Hypothetical protein	NA	1.21	0.01	Up
POX02410	Hypothetical protein	NA	1.17	0.00	Up
POX02414	Hypothetical protein	NA	-3.01	0.00	Down
POX02415	Hypothetical protein	NA	-3.07	0.00	Down
POX02417	Hypothetical protein	NA	1.66	0.02	Up
POX02418	Hypothetical protein	NA	2.09	0.00	Up
POX02421	Hypothetical protein	NA	1.23	0.00	Up
POX02431	Hypothetical protein	NA	1.85	0.00	Up
POX02432	Hypothetical protein	NA	1.35	0.00	Up
POX02442	Hypothetical protein	NA	2.52	0.03	Up
POX02453	Hypothetical protein	NA	1.05	0.00	Up
POX02461	Hypothetical protein	NA	-3.14	0.00	Down
POX02466	Hypothetical protein	NA	-1.15	0.00	Down
POX02468	Hypothetical protein	NA	-1.32	0.00	Down

POX02471	Hypothetical protein	NA	1.44	0.00	Up
POX02473	Hypothetical protein	NA	-1.70	0.00	Down
POX02475	Hypothetical protein	NA	-4.36	0.00	Down
POX02478	Hypothetical protein	NA	-1.38	0.00	Down
POX02483	Hypothetical protein	NA	-2.52	0.00	Down
POX02484	Zn2Cys6; Fungal transcriptional regulatory protein, N-terminal	NA	-3.57	0.00	Down
POX02485	Hypothetical protein	NA	-2.42	0.00	Down
POX02488	Hypothetical protein	NA	-4.21	0.00	Down
POX02490	Putative cellobiohydrolase	GH7	1.36	0.04	Up
POX02497	Hypothetical protein	NA	1.62	0.00	Up
POX02512	Hypothetical protein	NA	1.47	0.00	Up
POX02519	Hypothetical protein	NA	1.04	0.00	Up
POX02520	Hypothetical protein	NA	-2.37	0.00	Down
POX02525	Hypothetical protein	NA	-2.00	0.00	Down
POX02529	Hypothetical protein	NA	-1.71	0.00	Down
POX02533	Hypothetical protein	NA	1.10	0.00	Up
POX02537	Hypothetical protein	NA	-1.07	0.00	Down
POX02545	Hypothetical protein	NA	1.13	0.00	Up
POX02547	Hypothetical protein	NA	2.01	0.00	Up
POX02549	Hypothetical protein	NA	2.93	0.00	Up
POX02553	Hypothetical protein	NA	-1.35	0.00	Down
POX02558	Hypothetical protein	AA8	1.38	0.00	Up
POX02563	Hypothetical protein	NA	-1.22	0.00	Down
POX02564	Hypothetical protein	NA	-1.14	0.01	Down
POX02568	Hypothetical protein	NA	-1.13	0.00	Down
POX02569	Hypothetical protein	NA	1.59	0.00	Up
POX02570	Hypothetical protein	NA	1.16	0.00	Up
POX02577	Hypothetical protein	NA	-3.29	0.00	Down
POX02593	Hypothetical protein	NA	-2.63	0.00	Down
POX02595	Hypothetical protein	NA	-4.75	0.00	Down
POX02600	Hypothetical protein	NA	-1.05	0.01	Down
POX02610	Hypothetical protein	NA	2.93	0.00	Up
POX02611	Hypothetical protein	NA	1.07	0.03	Up
POX02623	Hypothetical protein	NA	2.58	0.00	Up
POX02628	Hypothetical protein	NA	2.26	0.00	Up
POX02633	Hypothetical protein	NA	1.73	0.00	Up
POX02640	Hypothetical protein	NA	-1.02	0.00	Down
POX02668	Putative chitin glucanosyltransferase	GH16	-1.04	0.00	Down
POX02675	Hypothetical protein	NA	1.64	0.00	Up
POX02689	Hypothetical protein	NA	-1.97	0.00	Down

POX02710	Putative alpha-1, 3-glucanase	GH71	-3.41	0.00	Down
POX02718	Hypothetical protein	NA	-1.86	0.00	Down
POX02724	Hypothetical protein	NA	-1.24	0.00	Down
POX02730	Hypothetical protein	NA	-1.02	0.00	Down
POX02735	Hypothetical protein	NA	1.82	0.00	Up
POX02736	Hypothetical protein	NA	-1.62	0.00	Down
POX02740	Putative endo-beta-1,4-glucanase	GH5	-1.54	0.00	Down
POX02741	Hypothetical protein	NA	2.67	0.00	Up
POX02754	Hypothetical protein	NA	-1.20	0.00	Down
POX02757	Hypothetical protein	NA	-3.88	0.00	Down
POX02762	Hypothetical protein	NA	-1.03	0.01	Down
POX02776	Hypothetical protein	NA	-1.51	0.00	Down
POX02783	Hypothetical protein	NA	-1.71	0.00	Down
POX02798	Hypothetical protein	NA	-1.02	0.00	Down
POX02803	Hypothetical protein	NA	-1.05	0.00	Down
POX02810	Hypothetical protein	NA	2.07	0.00	Up
POX02826	Hypothetical protein	NA	1.19	0.00	Up
POX02833	Hypothetical protein	NA	4.19	0.00	Up
POX02834	Hypothetical protein	NA	4.47	0.00	Up
POX02835	Hypothetical protein	NA	1.18	0.05	Up
POX02837	Hypothetical protein	NA	-4.22	0.00	Down
POX02847	Hypothetical protein	NA	-2.77	0.00	Down
POX02848	Putative chitinase	CBM18;CBM50;GH18	-1.66	0.00	Down
POX02850	Hypothetical protein	NA	1.51	0.02	Up
POX02852	Hypothetical protein	NA	-1.22	0.00	Down
POX02853	Hypothetical protein	NA	-1.75	0.00	Down
POX02855	Hypothetical protein	NA	1.57	0.00	Up
POX02858	Hypothetical protein	NA	-3.19	0.00	Down
POX02860	Hypothetical protein	NA	-4.28	0.00	Down
POX02861	Hypothetical protein	NA	-1.08	0.00	Down
POX02862	Hypothetical protein	NA	-1.13	0.00	Down
POX02867	Hypothetical protein	NA	2.47	0.00	Up
POX02905	Hypothetical protein	NA	3.71	0.00	Up
POX02912	Hypothetical protein	NA	1.15	0.00	Up
POX02923	Hypothetical protein	NA	1.53	0.00	Up
POX02924	Hypothetical protein	NA	1.37	0.00	Up
POX02928	Hypothetical protein	NA	1.22	0.00	Up
POX02930	Hypothetical protein	NA	-1.29	0.00	Down
POX02931	Hypothetical protein	NA	2.33	0.02	Up
POX02950	Hypothetical protein	NA	1.62	0.00	Up

POX02951	Hypothetical protein	NA	1.13	0.00	Up
POX02972	Hypothetical protein	NA	-3.08	0.00	Down
POX02981	Hypothetical protein	NA	3.66	0.00	Up
POX02985	Hypothetical protein	NA	-4.79	0.00	Down
POX02986	Hypothetical protein	NA	-4.95	0.00	Down
POX02989	Hypothetical protein	NA	2.99	0.00	Up
POX02991	Hypothetical protein	NA	2.02	0.01	Up
POX02995	Hypothetical protein	CE1	1.29	0.00	Up
POX02996	Hypothetical protein	NA	1.77	0.00	Up
POX02999	Hypothetical protein	NA	-7.66	0.00	Down
POX03000	Hypothetical protein	NA	-1.23	0.02	Down
POX03001	Hypothetical protein	NA	-1.13	0.00	Down
POX03005	Putative cutinase	CE5	-2.95	0.00	Down
POX03006	Hypothetical protein	NA	-1.03	0.00	Down
POX03012	Hypothetical protein	NA	1.28	0.00	Up
POX03015	Hypothetical protein	NA	1.04	0.00	Up
POX03018	Hypothetical protein	NA	2.04	0.00	Up
POX03020	Hypothetical protein	NA	-1.69	0.00	Down
POX03021	Putative chitinase	GH18	1.74	0.00	Up
POX03023	Zinc finger, C2H2-type	NA	-1.33	0.00	Down
POX03030	Hypothetical protein	NA	1.02	0.00	Up
POX03040	Hypothetical protein	NA	1.40	0.00	Up
POX03051	Hypothetical protein	NA	1.37	0.00	Up
POX03052	Hypothetical protein	NA	-1.54	0.00	Down
POX03054	Hypothetical protein	NA	1.53	0.00	Up
POX03060	Hypothetical protein	NA	-1.42	0.00	Down
POX03062	Putative beta-glucosidase	GH1	-1.56	0.00	Down
POX03067	Hypothetical protein	NA	-2.81	0.02	Down
POX03068	Hypothetical protein	NA	-3.46	0.00	Down
POX03075	Hypothetical protein	NA	3.24	0.00	Up
POX03077	Hypothetical protein	NA	-1.37	0.00	Down
POX03080	putative G protein complex alpha subunit	NA	-1.21	0.00	Down
POX03085	Hypothetical protein	NA	1.20	0.00	Up
POX03086	Hypothetical protein	NA	1.23	0.00	Up
POX03089	Hypothetical protein	NA	-1.06	0.00	Down
POX03090	Hypothetical protein	NA	-1.46	0.00	Down
POX03092	Hypothetical protein	NA	3.05	0.00	Up
POX03094	Hypothetical protein	NA	-2.41	0.00	Down
POX03095	Hypothetical protein	NA	-2.65	0.00	Down
POX03096	Sugar/inositol transporter	NA	-2.48	0.00	Down

POX03097	Hypothetical protein	NA	1.42	0.00	Up
POX03099	Hypothetical protein	NA	1.31	0.00	Up
POX03104	Hypothetical protein	NA	1.34	0.00	Up
POX03106	Hypothetical protein	NA	2.56	0.00	Up
POX03110	Hypothetical protein	NA	1.33	0.00	Up
POX03111	Hypothetical protein	NA	1.04	0.00	Up
POX03119	Hypothetical protein	NA	1.04	0.03	Up
POX03130	Hypothetical protein	NA	1.14	0.00	Up
POX03134	Hypothetical protein	NA	2.87	0.00	Up
POX03139	Hypothetical protein	NA	-2.08	0.00	Down
POX03155	Hypothetical protein	NA	-1.67	0.00	Down
POX03177	Putative dolichyl-phosphate beta-D-mannosyltransferase	GT27;GT2;GT81	-1.38	0.00	Down
POX03182	Hypothetical protein	NA	-1.00	0.00	Down
POX03183	Hypothetical protein	NA	-1.49	0.02	Down
POX03193	Hypothetical protein	NA	-1.02	0.00	Down
POX03202	Hypothetical protein	NA	1.36	0.00	Up
POX03209	Hypothetical protein	NA	-1.22	0.00	Down
POX03214	Hypothetical protein	NA	1.04	0.00	Up
POX03221	Hypothetical protein	NA	1.19	0.00	Up
POX03222	Hypothetical protein	NA	-1.06	0.00	Down
POX03224	Hypothetical protein	NA	1.10	0.00	Up
POX03227	Hypothetical protein	NA	1.23	0.01	Up
POX03228	Putative alpha-1, 3-glucan synthase	GH13;GT4;GT5	-1.04	0.00	Down
POX03231	Putative alpha-amylase	GH13;GH70	1.02	0.00	Up
POX03232	Hypothetical protein	NA	-1.82	0.00	Down
POX03233	Hypothetical protein	NA	1.32	0.00	Up
POX03235	Hypothetical protein	NA	1.01	0.01	Up
POX03243	Hypothetical protein	NA	-1.11	0.00	Down
POX03248	Hypothetical protein	NA	-1.25	0.00	Down
POX03250	Hypothetical protein	NA	-6.73	0.00	Down
POX03251	Hypothetical protein	NA	-1.47	0.00	Down
POX03252	Hypothetical protein	NA	-1.01	0.00	Down
POX03255	Major facilitator, sugar transporter-like	NA	-1.29	0.00	Down
POX03259	Hypothetical protein	NA	-3.31	0.00	Down
POX03262	Hypothetical protein	NA	2.22	0.00	Up
POX03268	Hypothetical protein	NA	-1.16	0.00	Down
POX03270	Mitochondrial carrier protein	NA	-1.05	0.00	Down
POX03274	Hypothetical protein	NA	-3.51	0.00	Down
POX03280	Hypothetical protein	NA	2.46	0.00	Up
POX03281	Hypothetical protein	NA	3.94	0.00	Up

POX03288	Hypothetical protein	NA	1.42	0.00	Up
POX03289	Hypothetical protein	NA	1.04	0.00	Up
POX03292	Hypothetical protein	NA	-1.83	0.00	Down
POX03293	Hypothetical protein	NA	-1.33	0.00	Down
POX03298	Hypothetical protein	NA	3.40	0.00	Up
POX03306	Hypothetical protein	NA	1.81	0.00	Up
POX03314	Hypothetical protein	NA	1.54	0.00	Up
POX03317	Hypothetical protein	NA	-1.23	0.00	Down
POX03322	Hypothetical protein	NA	1.83	0.00	Up
POX03325	Hypothetical protein	NA	-2.02	0.00	Down
POX03344	Hypothetical protein	NA	-2.62	0.00	Down
POX03345	Hypothetical protein	NA	-7.87	0.00	Down
POX03346	Hypothetical protein	NA	-1.41	0.00	Down
POX03347	Hypothetical protein	NA	1.36	0.00	Up
POX03349	Hypothetical protein	NA	-1.28	0.01	Down
POX03351	Hypothetical protein	NA	1.25	0.00	Up
POX03357	Hypothetical protein	NA	3.25	0.00	Up
POX03363	Hypothetical protein	NA	3.55	0.00	Up
POX03364	Hypothetical protein	NA	1.52	0.01	Up
POX03377	Hypothetical protein	NA	1.27	0.00	Up
POX03388	Hypothetical protein	NA	1.24	0.00	Up
POX03389	Hypothetical protein	NA	-1.56	0.00	Down
POX03395	Hypothetical protein	NA	-2.50	0.00	Down
POX03396	Hypothetical protein	NA	-2.54	0.00	Down
POX03402	Hypothetical protein	NA	-1.68	0.00	Down
POX03403	Hypothetical protein	NA	-1.06	0.04	Down
POX03411	Hypothetical protein	CE10	-2.93	0.00	Down
POX03412	Hypothetical protein	NA	-7.14	0.00	Down
POX03413	Hypothetical protein	NA	-9.60	0.00	Down
POX03414	Hypothetical protein	NA	-1.60	0.00	Down
POX03416	Hypothetical protein	NA	1.11	0.00	Up
POX03419	Hypothetical protein	NA	3.64	0.00	Up
POX03420	Putative exo-alpha-L-1,5-arabinanase	GH33;GH74;GH93	2.47	0.00	Up
POX03421	Hypothetical protein	NA	-2.14	0.00	Down
POX03426	Hypothetical protein	NA	-2.06	0.00	Down
POX03432	Hypothetical protein	NA	-1.48	0.00	Down
POX03434	Hypothetical protein	NA	-8.95	0.00	Down
POX03435	Hypothetical protein	NA	-6.13	0.00	Down
POX03437	Hypothetical protein	NA	-1.20	0.00	Down
POX03444	Hypothetical protein	NA	-1.55	0.00	Down

POX03445	Hypothetical protein	NA		-1.51	0.00	Down
POX03452	Putative oligo-alpha-1, 6-glucosidase	GH13		1.54	0.00	Up
POX03455	Hypothetical protein	NA		1.54	0.00	Up
POX03456	Hypothetical protein	NA		-2.16	0.00	Down
POX03459	Hypothetical protein	NA		-2.52	0.00	Down
POX03460	Hypothetical protein	NA		-4.86	0.00	Down
POX03462	Hypothetical protein	NA		5.36	0.00	Up
POX03464	Hypothetical protein	NA		-2.51	0.00	Down
POX03465	Hypothetical protein	NA		-3.18	0.00	Down
POX03473	Hypothetical protein	NA		-1.26	0.00	Down
POX03474	Hypothetical protein	NA		2.59	0.00	Up
POX03477	Hypothetical protein	NA		-1.22	0.02	Down
POX03486	Hypothetical protein	NA		1.76	0.03	Up
POX03489	Hypothetical protein	NA		1.67	0.00	Up
POX03493	Hypothetical protein	NA		1.16	0.00	Up
POX03496	Hypothetical protein	NA		-4.30	0.00	Down
POX03501	Hypothetical protein	NA		-2.45	0.00	Down
POX03505	Hypothetical protein	NA		2.92	0.00	Up
POX03521	Hypothetical protein	NA		2.04	0.00	Up
POX03524	Hypothetical protein	NA		-2.77	0.00	Down
POX03530	Hypothetical protein	NA		1.50	0.00	Up
POX03532	Hypothetical protein	NA		-6.45	0.00	Down
POX03540	Hypothetical protein	NA		-1.22	0.04	Down
POX03546	Hypothetical protein	NA		1.15	0.00	Up
POX03572	Hypothetical protein	NA		-4.31	0.00	Down
POX03575	Hypothetical protein	NA		-3.45	0.00	Down
POX03576	Hypothetical protein	NA		1.69	0.00	Up
POX03580	Hypothetical protein	NA		1.09	0.00	Up
POX03588	Hypothetical protein	NA		1.03	0.00	Up
POX03593	Hypothetical protein	NA		1.96	0.00	Up
POX03596	Hypothetical protein	NA		-1.32	0.00	Down
POX03598	Hypothetical protein	NA		-1.41	0.00	Down
POX03600	Hypothetical protein	NA		-2.50	0.00	Down
POX03603	Hypothetical protein	NA		-3.34	0.00	Down
POX03616	Hypothetical protein	NA		1.44	0.00	Up
POX03617	Hypothetical protein	NA		2.36	0.00	Up
POX03619	Hypothetical protein	NA		1.83	0.00	Up
POX03626	Zn2Cys6; Fungal transcriptional regulatory protein, N-terminal	NA		-1.37	0.00	Down
POX03628	Hypothetical protein	NA		-1.13	0.00	Down
POX03629	Hypothetical protein	NA		1.86	0.00	Up

POX03632	Hypothetical protein	NA	-5.86	0.00	Down
POX03633	Hypothetical protein	NA	2.65	0.00	Up
POX03639	Hypothetical protein	NA	-1.49	0.00	Down
POX03645	Hypothetical protein	NA	1.64	0.02	Up
POX03646	Hypothetical protein	NA	1.44	0.00	Up
POX03658	Hypothetical protein	NA	-1.10	0.00	Down
POX03666	Hypothetical protein	NA	4.07	0.00	Up
POX03668	Hypothetical protein	NA	1.26	0.00	Up
POX03669	Hypothetical protein	NA	3.27	0.00	Up
POX03682	Hypothetical protein	NA	2.18	0.00	Up
POX03689	Hypothetical protein	NA	2.50	0.00	Up
POX03697	Hypothetical protein	NA	-1.66	0.01	Down
POX03703	Hypothetical protein	NA	3.02	0.01	Up
POX03706	Hypothetical protein	NA	1.37	0.00	Up
POX03728	Hypothetical protein	NA	1.13	0.00	Up
POX03729	Hypothetical protein	NA	-1.48	0.00	Down
POX03732	Putative UDP-GalNAc: alpha-1, 4-N-acetylgalactosaminyltransferase	GT32	1.51	0.00	Up
POX03733	Putative N-acetyl-1-D-myo-inosityl-2-amino-2-deoxy-alpha-D-glucopyranoside deacetylase	CE14	1.26	0.00	Up
POX03734	Putative GDP-Man: alpha-1, 3-mannosyltransferase	GT69	1.12	0.00	Up
POX03738	Putative endo-alpha-1, 4-polygalactosaminidase	GH114	-2.20	0.00	Down
POX03740	Zn2Cys6; Fungal specific transcription factor	NA	-1.31	0.00	Down
POX03745	Hypothetical protein	NA	2.40	0.00	Up
POX03749	Hypothetical protein	NA	-3.07	0.00	Down
POX03755	Hypothetical protein	NA	1.01	0.00	Up
POX03762	Hypothetical protein	AA8	1.25	0.00	Up
POX03764	Hypothetical protein	NA	2.41	0.01	Up
POX03769	Hypothetical protein	NA	2.07	0.00	Up
POX03773	Hypothetical protein	NA	-1.30	0.00	Down
POX03775	Putative chitinase	GH18	3.18	0.00	Up
POX03776	Hypothetical protein	NA	-6.42	0.00	Down
POX03777	Hypothetical protein	NA	-7.70	0.00	Down
POX03778	Hypothetical protein	NA	-6.10	0.00	Down
POX03782	Hypothetical protein	NA	5.26	0.00	Up
POX03791	Hypothetical protein	NA	1.30	0.00	Up
POX03793	Hypothetical protein	NA	2.39	0.00	Up
POX03796	Hypothetical protein	NA	-1.99	0.00	Down
POX03797	Hypothetical protein	NA	-1.76	0.00	Down
POX03804	Hypothetical protein	NA	-1.45	0.00	Down

POX03805	Hypothetical protein	NA	-1.52	0.00	Down
POX03814	Hypothetical protein	NA	1.55	0.00	Up
POX03815	Hypothetical protein	NA	1.88	0.00	Up
POX03817	Hypothetical protein	NA	1.09	0.00	Up
POX03818	Hypothetical protein	NA	-1.86	0.00	Down
POX03844	Hypothetical protein	NA	-8.18	0.00	Down
POX03849	Hypothetical protein	NA	-2.52	0.00	Down
POX03852	Hypothetical protein	NA	1.04	0.00	Up
POX03854	Hypothetical protein	NA	6.79	0.00	Up
POX03855	Hypothetical protein	NA	6.56	0.00	Up
POX03856	Hypothetical protein	NA	4.17	0.00	Up
POX03857	Hypothetical protein	NA	2.67	0.00	Up
POX03858	Hypothetical protein	NA	5.09	0.00	Up
POX03859	Hypothetical protein	NA	6.19	0.00	Up
POX03860	Hypothetical protein	NA	3.52	0.00	Up
POX03861	Hypothetical protein	NA	2.59	0.00	Up
POX03863	Hypothetical protein	NA	1.80	0.00	Up
POX03864	Hypothetical protein	NA	5.32	0.00	Up
POX03865	beta-ketoacyl synthase	NA	2.51	0.00	Up
POX03866	Hypothetical protein	NA	2.92	0.00	Up
POX03867	Hypothetical protein	NA	8.30	0.00	Up
POX03868	Hypothetical protein	NA	5.10	0.00	Up
POX03873	Zn2Cys6; Fungal transcriptional regulatory protein, N-terminal	NA	1.54	0.00	Up
POX03874	Hypothetical protein	NA	4.99	0.00	Up
POX03875	Hypothetical protein	NA	2.84	0.00	Up
POX03886	Homeodomain-like	NA	-4.35	0.00	Down
POX03894	Hypothetical protein	NA	-1.25	0.00	Down
POX03898	Hypothetical protein	NA	-4.42	0.00	Down
POX03900	Hypothetical protein	NA	-4.23	0.00	Down
POX03905	Hypothetical protein	NA	-2.44	0.00	Down
POX03915	Hypothetical protein	NA	-1.27	0.00	Down
POX03916	Hypothetical protein	NA	1.62	0.00	Up
POX03917	Hypothetical protein	NA	2.12	0.00	Up
POX03918	Hypothetical protein	NA	1.04	0.00	Up
POX03931	Hypothetical protein	NA	1.19	0.00	Up
POX03933	Hypothetical protein	NA	5.98	0.00	Up
POX03934	Hypothetical protein	NA	5.07	0.00	Up
POX03935	Hypothetical protein	NA	7.76	0.00	Up
POX03946	Hypothetical protein	NA	-2.74	0.01	Down
POX03954	Zn2Cys6; Fungal transcriptional regulatory protein, N-terminal	NA	1.39	0.00	Up

POX03959	Zn2Cys6; Fungal transcriptional regulatory protein, N-terminal	NA	-2.78	0.00	Down
POX03966	Hypothetical protein	NA	1.06	0.00	Up
POX03968	Hypothetical protein	NA	1.67	0.00	Up
POX03976	Hypothetical protein	NA	-6.57	0.00	Down
POX03999	Zn2Cys6; Fungal transcriptional regulatory protein, N-terminal	NA	-1.97	0.00	Down
POX04010	Putative carbohydrate acetylesterase	CE16	-2.25	0.00	Down
POX04012	Hypothetical protein	NA	1.07	0.00	Up
POX04013	Hypothetical protein	NA	1.39	0.00	Up
POX04020	Hypothetical protein	NA	-3.01	0.01	Down
POX04034	Hypothetical protein	NA	2.48	0.00	Up
POX04040	Hypothetical protein	NA	1.62	0.00	Up
POX04051	Hypothetical protein	NA	1.54	0.00	Up
POX04053	Hypothetical protein	NA	-1.11	0.02	Down
POX04054	Hypothetical protein	NA	1.03	0.00	Up
POX04064	Hypothetical protein	NA	-2.36	0.00	Down
POX04067	Hypothetical protein	NA	-1.22	0.00	Down
POX04070	Hypothetical protein	NA	2.99	0.00	Up
POX04079	Hypothetical protein	NA	1.72	0.00	Up
POX04082	Hypothetical protein	NA	3.03	0.00	Up
POX04083	Hypothetical protein	NA	1.13	0.01	Up
POX04084	Hypothetical protein	NA	-1.70	0.00	Down
POX04087	Hypothetical protein	NA	-1.79	0.00	Down
POX04088	Hypothetical protein	NA	1.60	0.00	Up
POX04103	Hypothetical protein	NA	-1.68	0.02	Down
POX04104	Hypothetical protein	NA	-3.32	0.00	Down
POX04107	Hypothetical protein	NA	1.61	0.00	Up
POX04108	Hypothetical protein	NA	-1.18	0.01	Down
POX04109	Hypothetical protein	NA	-1.31	0.00	Down
POX04114	Hypothetical protein	NA	1.68	0.00	Up
POX04125	Hypothetical protein	NA	1.47	0.00	Up
POX04129	Hypothetical protein	NA	-1.08	0.00	Down
POX04135	Hypothetical protein	NA	1.37	0.00	Up
POX04136	Hypothetical protein	NA	1.64	0.00	Up
POX04143	Hypothetical protein	NA	-1.46	0.00	Down
POX04146	Hypothetical protein	NA	-1.43	0.00	Down
POX04147	Hypothetical protein	NA	-3.30	0.00	Down
POX04151	Hypothetical protein	NA	1.23	0.03	Up
POX04177	Hypothetical protein	CE10;CE1	2.45	0.00	Up
POX04181	Hypothetical protein	NA	-1.09	0.00	Down
POX04182	Hypothetical protein	NA	1.25	0.00	Up

POX04194	Hypothetical protein	NA	-1.40	0.00	Down
POX04195	Hypothetical protein	NA	-1.20	0.00	Down
POX04197	Hypothetical protein	NA	-1.53	0.00	Down
POX04221	Hypothetical protein	NA	4.38	0.00	Up
POX04222	Hypothetical protein	NA	1.72	0.00	Up
POX04223	Zinc finger, C2H2-type	NA	1.16	0.00	Up
POX04224	Hypothetical protein	NA	-1.27	0.00	Down
POX04225	Hypothetical protein	NA	-1.40	0.00	Down
POX04226	Hypothetical protein	NA	-4.63	0.00	Down
POX04227	Winged helix repressor DNA-binding	NA	-3.33	0.00	Down
POX04230	Hypothetical protein	NA	-1.22	0.00	Down
POX04232	Hypothetical protein	NA	-1.83	0.02	Down
POX04234	Hypothetical protein	NA	1.24	0.00	Up
POX04235	Hypothetical protein	NA	3.73	0.00	Up
POX04240	Hypothetical protein	NA	1.95	0.00	Up
POX04241	Hypothetical protein	NA	1.69	0.00	Up
POX04244	Putative alpha-mannosyltransferase	GT71	1.74	0.00	Up
POX04258	Hypothetical protein	NA	-1.41	0.00	Down
POX04268	Hypothetical protein	NA	1.43	0.02	Up
POX04276	Sugar/inositol transporter	NA	-1.55	0.00	Down
POX04282	Hypothetical protein	NA	1.47	0.00	Up
POX04289	Hypothetical protein	NA	2.47	0.00	Up
POX04292	Hypothetical protein	NA	-6.22	0.00	Down
POX04314	Hypothetical protein	NA	2.28	0.00	Up
POX04315	Hypothetical protein	NA	2.47	0.03	Up
POX04319	Hypothetical protein	NA	-1.35	0.00	Down
POX04320	Hypothetical protein	NA	-1.28	0.00	Down
POX04322	Hypothetical protein	NA	1.89	0.00	Up
POX04323	Hypothetical protein	NA	1.04	0.00	Up
POX04339	Hypothetical protein	NA	-1.26	0.00	Down
POX04347	Hypothetical protein	NA	3.21	0.00	Up
POX04362	Hypothetical protein	NA	-1.28	0.00	Down
POX04365	Hypothetical protein	NA	1.72	0.03	Up
POX04369	Sugar/inositol transporter	NA	-3.78	0.00	Down
POX04370	Hypothetical protein	NA	3.18	0.01	Up
POX04374	Hypothetical protein	NA	-1.49	0.00	Down
POX04375	Hypothetical protein	NA	2.76	0.00	Up
POX04380	Hypothetical protein	NA	1.44	0.00	Up
POX04387	Hypothetical protein	NA	-3.05	0.00	Down
POX04388	Hypothetical protein	NA	-5.47	0.00	Down

POX04389	Hypothetical protein	NA	-3.13	0.00	Down
POX04408	Hypothetical protein	NA	-1.45	0.00	Down
POX04416	Hypothetical protein	NA	1.34	0.03	Up
POX04420	Zinc finger, C2H2-type	NA	-1.46	0.00	Down
POX04431	Hypothetical protein	NA	1.35	0.02	Up
POX04446	Hypothetical protein	NA	1.65	0.00	Up
POX04452	Hypothetical protein	NA	-1.33	0.00	Down
POX04456	Hypothetical protein	NA	-2.91	0.00	Down
POX04467	Hypothetical protein	NA	-1.55	0.00	Down
POX04478	Hypothetical protein	NA	-3.41	0.00	Down
POX04479	Hypothetical protein	NA	-1.28	0.02	Down
POX04480	Hypothetical protein	NA	1.33	0.00	Up
POX04485	Hypothetical protein	NA	-2.70	0.00	Down
POX04499	Hypothetical protein	NA	-5.13	0.00	Down
POX04513	Zn2Cys6; Fungal transcriptional regulatory protein, N-terminal	NA	-2.01	0.00	Down
POX04516	Hypothetical protein	NA	-1.05	0.00	Down
POX04521	Hypothetical protein	NA	-1.43	0.00	Down
POX04522	Hypothetical protein	NA	-1.59	0.00	Down
POX04524	Hypothetical protein	NA	-2.06	0.00	Down
POX04526	Hypothetical protein	NA	-2.48	0.00	Down
POX04529	Sugar/inositol transporter	NA	-1.96	0.00	Down
POX04531	Sugar/inositol transporter	NA	-2.32	0.00	Down
POX04532	Carbohydrate binding domain-containing protein	NA	-1.11	0.00	Down
POX04533	Sugar/inositol transporter	NA	2.02	0.00	Up
POX04536	Hypothetical protein	CBM24;GH71	-2.10	0.00	Down
POX04539	Hypothetical protein	NA	1.90	0.00	Up
POX04540	Neutrophil cytosol factor 2	NA	1.50	0.00	Up
POX04552	Hypothetical protein	NA	-2.47	0.00	Down
POX04556	Hypothetical protein	NA	1.11	0.00	Up
POX04559	putative beta-1, 3-galactosyltransferase	NA	-2.91	0.00	Down
POX04560	Hypothetical protein	NA	-6.41	0.00	Down
POX04561	Hypothetical protein	NA	-5.80	0.00	Down
POX04562	Hypothetical protein	NA	-6.83	0.00	Down
POX04565	Hypothetical protein	NA	2.25	0.00	Up
POX04566	Hypothetical protein	NA	2.08	0.00	Up
POX04567	Zn2Cys6; Fungal transcriptional regulatory protein, N-terminal	NA	2.72	0.00	Up
POX04568	Hypothetical protein	NA	-4.71	0.00	Down
POX04569	Hypothetical protein	NA	-2.88	0.00	Down
POX04570	Putative exopolygalacturonase	GH28	-1.76	0.02	Down
POX04571	Hypothetical protein	NA	-1.55	0.02	Down

POX04573	Hypothetical protein	NA	-1.29	0.00	Down
POX04577	Hypothetical protein	NA	-2.91	0.00	Down
POX04578	Hypothetical protein	NA	2.11	0.00	Up
POX04581	Hypothetical protein	NA	-1.47	0.00	Down
POX04583	Hypothetical protein	NA	1.44	0.00	Up
POX04589	Hypothetical protein	NA	-3.12	0.00	Down
POX04590	Zn2Cys6; Fungal transcriptional regulatory protein, N-terminal	NA	-1.42	0.00	Down
POX04591	Hypothetical protein	NA	1.60	0.02	Up
POX04605	Hypothetical protein	NA	2.15	0.00	Up
POX04622	Zn2Cys6; Fungal transcriptional regulatory protein, N-terminal	NA	1.35	0.00	Up
POX04628	Hypothetical protein	NA	-1.95	0.00	Down
POX04629	Hypothetical protein	NA	-1.49	0.00	Down
POX04639	Hypothetical protein	NA	1.59	0.03	Up
POX04654	Hypothetical protein	NA	-1.26	0.03	Down
POX04662	Hypothetical protein	NA	-6.47	0.00	Down
POX04671	Hypothetical protein	NA	2.60	0.01	Up
POX04673	Sphingolipid long chain base-responsive protein LSP1-like	NA	-2.23	0.00	Down
POX04676	Zn2Cys6; Fungal transcriptional regulatory protein, N-terminal	NA	1.42	0.00	Up
POX04678	trans-2-enoyl-CoA reductase, mitochondrial-like	NA	-1.21	0.01	Down
POX04681	Hypothetical protein	NA	-1.72	0.00	Down
POX04686	Hypothetical protein	GH128	-1.82	0.00	Down
POX04693	Hypothetical protein	NA	-5.93	0.00	Down
POX04704	Hypothetical protein	NA	1.30	0.00	Up
POX04712	Hypothetical protein	NA	1.43	0.00	Up
POX04714	Hypothetical protein	NA	-1.26	0.00	Down
POX04720	Hypothetical protein	NA	1.35	0.00	Up
POX04726	Hypothetical protein	NA	-3.10	0.00	Down
POX04728	Hypothetical protein	NA	-1.43	0.00	Down
POX04735	Hypothetical protein	NA	-3.29	0.00	Down
POX04745	Hypothetical protein	NA	1.03	0.00	Up
POX04752	Putative alpha-L-arabinofuranosidase	GH51	-1.15	0.00	Down
POX04756	Hypothetical protein	NA	2.01	0.01	Up
POX04758	Glycoside hydrolase, family 71	CBM24;GH71	1.51	0.00	Up
POX04764	Hypothetical protein	NA	-2.00	0.00	Down
POX04766	Hypothetical protein	NA	-1.70	0.00	Down
POX04768	Hypothetical protein	NA	-1.17	0.00	Down
POX04770	Hypothetical protein	NA	-1.00	0.00	Down
POX04773	Hypothetical protein	NA	-1.98	0.00	Down
POX04774	Hypothetical protein	NA	1.18	0.00	Up
POX04778	Hypothetical protein	NA	1.26	0.00	Up

POX04781	Hypothetical protein	NA	2.27	0.00	Up
POX04786	Cellobiohydrolase Cel6A	CBM1;GH6	-1.46	0.00	Down
POX04799	Putative beta-glucuronidase	GH79	2.66	0.00	Up
POX04803	Hypothetical protein	NA	-1.58	0.00	Down
POX04808	Hypothetical protein	NA	1.17	0.00	Up
POX04823	Hypothetical protein	NA	-1.43	0.00	Down
POX04824	Hypothetical protein	NA	-2.86	0.00	Down
POX04826	Hypothetical protein	NA	-3.85	0.00	Down
POX04830	Hypothetical protein	NA	1.74	0.00	Up
POX04831	Hypothetical protein	NA	1.28	0.00	Up
POX04836	Hypothetical protein	NA	1.66	0.04	Up
POX04837	Hypothetical protein	NA	-4.79	0.00	Down
POX04838	Hypothetical protein	NA	-1.34	0.00	Down
POX04840	Hypothetical protein	NA	-1.13	0.00	Down
POX04847	Hypothetical protein	NA	1.40	0.00	Up
POX04856	Hypothetical protein	NA	-2.74	0.00	Down
POX04860	Homeodomain-like	NA	-3.02	0.00	Down
POX04866	Hypothetical protein	NA	1.33	0.00	Up
POX04867	Hypothetical protein	NA	1.16	0.00	Up
POX04868	Hypothetical protein	NA	1.39	0.00	Up
POX04869	Hypothetical protein	NA	2.21	0.00	Up
POX04879	Hypothetical protein	NA	1.02	0.00	Up
POX04883	Hypothetical protein	NA	3.82	0.00	Up
POX04886	Hypothetical protein	NA	1.22	0.00	Up
POX04887	Hypothetical protein	NA	-2.14	0.00	Down
POX04904	Hypothetical protein	NA	3.15	0.00	Up
POX04905	Hypothetical protein	NA	-1.17	0.01	Down
POX04912	Hypothetical protein	NA	2.36	0.00	Up
POX04915	Zn2Cys6; Fungal specific transcription factor	NA	3.33	0.00	Up
POX04916	Hypothetical protein	NA	-3.61	0.00	Down
POX04918	Sugar/inositol transporter	NA	-1.93	0.00	Down
POX04919	Hypothetical protein	NA	-1.22	0.01	Down
POX04923	Hypothetical protein	NA	-1.18	0.00	Down
POX04927	Hypothetical protein	NA	-5.77	0.00	Down
POX04942	Hypothetical protein	NA	2.66	0.00	Up
POX04943	Hypothetical protein	NA	1.37	0.00	Up
POX04944	Hypothetical protein	NA	-1.16	0.00	Down
POX04950	Hypothetical protein	NA	-1.23	0.00	Down
POX04960	Putative alpha-1, 2-mannosyltransferase	GT15	1.05	0.00	Up
POX04979	Hypothetical protein	NA	-1.31	0.00	Down

POX05007	Hypothetical protein	NA	1.55	0.00	Up
POX05011	Hypothetical protein	NA	-2.43	0.00	Down
POX05016	Hypothetical protein	NA	-7.10	0.00	Down
POX05017	Hypothetical protein	NA	-1.38	0.00	Down
POX05020	Hypothetical protein	NA	1.10	0.00	Up
POX05026	Hypothetical protein	NA	4.57	0.00	Up
POX05030	Hypothetical protein	NA	1.12	0.00	Up
POX05038	Hypothetical protein	NA	-1.42	0.03	Down
POX05045	Hypothetical protein	NA	-1.21	0.01	Down
POX05046	Hypothetical protein	NA	-1.12	0.00	Down
POX05048	Hypothetical protein	NA	-1.28	0.00	Down
POX05051	Hypothetical protein	NA	-1.93	0.00	Down
POX05062	Hypothetical protein	NA	-1.27	0.00	Down
POX05081	Hypothetical protein	NA	-1.38	0.00	Down
POX05082	Hypothetical protein	NA	2.16	0.00	Up
POX05090	Hypothetical protein	NA	-2.80	0.00	Down
POX05091	Hypothetical protein	NA	-2.59	0.00	Down
POX05092	Hypothetical protein	NA	-6.09	0.00	Down
POX05100	Hypothetical protein	NA	2.90	0.00	Up
POX05134	Hypothetical protein	NA	1.34	0.00	Up
POX05138	Hypothetical protein	NA	1.63	0.00	Up
POX05148	Hypothetical protein	NA	1.59	0.00	Up
POX05155	Hypothetical protein	NA	3.37	0.00	Up
POX05166	Hypothetical protein	NA	1.14	0.00	Up
POX05173	Hypothetical protein	NA	8.32	0.00	Up
POX05174	Hypothetical protein	NA	1.19	0.00	Up
POX05188	Putative chitin glucanosyltransferase	GH16	7.43	0.00	Up
POX05209	Hypothetical protein	NA	-1.13	0.00	Down
POX05220	Hypothetical protein	NA	-2.78	0.00	Down
POX05223	Hypothetical protein	NA	-3.91	0.00	Down
POX05232	Hypothetical protein	NA	-1.04	0.00	Down
POX05233	Hypothetical protein	NA	-1.26	0.00	Down
POX05236	Hypothetical protein	NA	-1.40	0.00	Down
POX05239	Hypothetical protein	NA	-2.13	0.01	Down
POX05240	Putative alpha-L-arabinofuranosidase	GH117;GH43	1.13	0.00	Up
POX05242	Hypothetical protein	NA	1.11	0.00	Up
POX05246	Hypothetical protein	NA	1.12	0.00	Up
POX05247	Hypothetical protein	NA	2.66	0.00	Up
POX05260	Hypothetical protein	AA7	-1.65	0.00	Down
POX05262	Hypothetical protein	NA	1.29	0.02	Up

POX05265	Hypothetical protein	NA	-1.66	0.00	Down
POX05269	Hypothetical protein	NA	-1.39	0.00	Down
POX05271	Hypothetical protein	NA	-2.04	0.00	Down
POX05275	Hypothetical protein	NA	-2.40	0.00	Down
POX05276	Hypothetical protein	NA	-1.48	0.00	Down
POX05277	Zn2Cys6; Fungal transcriptional regulatory protein, N-terminal	NA	-1.03	0.00	Down
POX05280	Hypothetical protein	NA	-1.49	0.00	Down
POX05286	Hypothetical protein	NA	-1.61	0.00	Down
POX05289	Hypothetical protein	NA	1.06	0.00	Up
POX05294	Putative chitinase	CBM18;GH18	1.87	0.01	Up
POX05295	Putative exo-beta-1, 3-glucanase	GH55	-1.24	0.03	Down
POX05298	Hypothetical protein	NA	1.06	0.03	Up
POX05300	Hypothetical protein	NA	1.43	0.05	Up
POX05306	Hypothetical protein	NA	-3.70	0.00	Down
POX05307	Hypothetical protein	NA	-1.14	0.00	Down
POX05316	Hypothetical protein	NA	-1.19	0.02	Down
POX05329	Hypothetical protein	NA	-1.92	0.03	Down
POX05332	Hypothetical protein	NA	-2.65	0.00	Down
POX05335	Hypothetical protein	NA	-4.92	0.00	Down
POX05342	Hypothetical protein	NA	1.12	0.01	Up
POX05344	Hypothetical protein	NA	1.32	0.02	Up
POX05355	Hypothetical protein	NA	3.36	0.00	Up
POX05358	Hypothetical protein	NA	-1.06	0.02	Down
POX05359	Hypothetical protein	NA	2.42	0.00	Up
POX05372	Putative alpha-1, 2 / alpha-1, 6-mannosyltransferase	GT62	-1.33	0.00	Down
POX05376	Hypothetical protein	NA	-1.97	0.00	Down
POX05377	Hypothetical protein	NA	1.85	0.00	Up
POX05385	Hypothetical protein	NA	1.14	0.00	Up
POX05391	Hypothetical protein	NA	1.04	0.00	Up
POX05421	Hypothetical protein	NA	1.13	0.00	Up
POX05430	Hypothetical protein	NA	-2.38	0.00	Down
POX05435	Hypothetical protein	NA	2.76	0.00	Up
POX05437	Hypothetical protein	NA	3.05	0.00	Up
POX05440	Hypothetical protein	NA	-1.06	0.00	Down
POX05441	Hypothetical protein	NA	1.42	0.00	Up
POX05442	Hypothetical protein	NA	3.21	0.00	Up
POX05444	Hypothetical protein	NA	1.69	0.00	Up
POX05446	Hypothetical protein	NA	1.46	0.00	Up
POX05464	Hypothetical protein	NA	-1.05	0.00	Down
POX05467	Hypothetical protein	NA	1.09	0.00	Up

POX05469	Hypothetical protein	NA	1.28	0.00	Up
POX05478	Hypothetical protein	NA	2.10	0.04	Up
POX05482	Hypothetical protein	NA	-4.28	0.00	Down
POX05483	Hypothetical protein	NA	-3.50	0.00	Down
POX05487	Hypothetical protein	NA	1.22	0.04	Up
POX05490	Hypothetical protein	NA	1.46	0.00	Up
POX05492	Hypothetical protein	NA	1.06	0.00	Up
POX05495	Hypothetical protein	NA	1.14	0.00	Up
POX05515	Sugar/inositol transporter	NA	1.36	0.00	Up
POX05529	Hypothetical protein	NA	1.37	0.00	Up
POX05537	Peptidoglycan binding domain-containing protein	CBM50	-3.42	0.00	Down
POX05538	Hypothetical protein	NA	-1.20	0.00	Down
POX05542	Hypothetical protein	NA	2.11	0.00	Up
POX05548	Hypothetical protein	NA	-1.34	0.00	Down
POX05550	Hypothetical protein	NA	1.92	0.00	Up
POX05559	Hypothetical protein	NA	1.07	0.00	Up
POX05563	Hypothetical protein	NA	1.11	0.00	Up
POX05565	Hypothetical protein	NA	-1.03	0.00	Down
POX05567	Hypothetical protein	NA	-1.10	0.00	Down
POX05572	Hypothetical protein	NA	1.36	0.00	Up
POX05573	Hypothetical protein	NA	1.92	0.00	Up
POX05576	Hypothetical protein	NA	2.09	0.00	Up
POX05580	Putative polygalacturonase	GH28	2.70	0.00	Up
POX05581	Hypothetical protein	NA	-1.14	0.00	Down
POX05586	Hypothetical protein	NA	1.02	0.00	Up
POX05587	Cellobiohydrolase CBHI/Cel7A-2	CBM1;GH7	-2.05	0.00	Down
POX05591	Hypothetical protein	NA	-1.20	0.00	Down
POX05598	Hypothetical protein	NA	-1.88	0.01	Down
POX05601	Hypothetical protein	NA	-1.16	0.00	Down
POX05604	Putative beta-1, 3-glucanosyltransglycosylase	GH72	-1.25	0.00	Down
POX05609	Hypothetical protein	NA	-1.22	0.00	Down
POX05611	Hypothetical protein	NA	3.10	0.00	Up
POX05623	Zinc finger, C2H2-type	NA	-2.84	0.00	Down
POX05626	Hypothetical protein	NA	-1.89	0.00	Down
POX05629	Zn2Cys6; Fungal transcriptional regulatory protein, N-terminal	NA	-4.67	0.00	Down
POX05632	Hypothetical protein	NA	1.07	0.00	Up
POX05641	Hypothetical protein	NA	-1.06	0.00	Down
POX05644	Hypothetical protein	NA	1.15	0.00	Up
POX05645	Hypothetical protein	AA11	-2.79	0.00	Down
POX05646	Hypothetical protein	NA	-3.95	0.00	Down

POX05654	Hypothetical protein	NA	-2.23	0.00	Down
POX05664	Hypothetical protein	NA	2.28	0.00	Up
POX05667	Hypothetical protein	NA	1.11	0.00	Up
POX05668	Hypothetical protein	NA	1.09	0.00	Up
POX05680	Hypothetical protein	NA	-1.28	0.03	Down
POX05683	Hypothetical protein	NA	1.15	0.03	Up
POX05693	Hypothetical protein	NA	-2.48	0.04	Down
POX05696	Hypothetical protein	NA	-1.89	0.00	Down
POX05700	Putative N-acetyl-glucosamine-6-phosphate deacetylase	CE9	-1.82	0.00	Down
POX05702	Hypothetical protein	NA	2.40	0.00	Up
POX05703	Hypothetical protein	NA	-1.87	0.00	Down
POX05707	Hypothetical protein	NA	-1.58	0.00	Down
POX05717	Hypothetical protein	NA	-2.70	0.00	Down
POX05726	Zinc finger, C2H2-type	NA	-5.96	0.00	Down
POX05728	Hypothetical protein	NA	-2.12	0.00	Down
POX05736	Hypothetical protein	NA	-2.11	0.01	Down
POX05738	Hypothetical protein	NA	1.65	0.00	Up
POX05740	Hypothetical protein	NA	-1.05	0.00	Down
POX05741	Hypothetical protein	NA	-4.95	0.00	Down
POX05744	Hypothetical protein	NA	1.40	0.00	Up
POX05750	Hypothetical protein	NA	-1.07	0.00	Down
POX05754	Hypothetical protein	NA	2.54	0.00	Up
POX05756	Hypothetical protein	NA	-5.38	0.00	Down
POX05767	Hypothetical protein	NA	-2.43	0.00	Down
POX05776	Hypothetical protein	NA	-1.27	0.00	Down
POX05778	Hypothetical protein	NA	-1.44	0.00	Down
POX05781	Hypothetical protein	NA	1.40	0.00	Up
POX05782	Hypothetical protein	NA	3.37	0.00	Up
POX05795	Hypothetical protein	NA	1.46	0.03	Up
POX05796	Hypothetical protein	NA	1.61	0.01	Up
POX05797	Hypothetical protein	NA	1.42	0.05	Up
POX05804	Hypothetical protein	NA	-1.32	0.00	Down
POX05824	Hypothetical protein	NA	2.00	0.00	Up
POX05829	Hypothetical protein	NA	1.04	0.00	Up
POX05835	Hypothetical protein	NA	1.88	0.01	Up
POX05844	Nucleic acid-binding, OB-fold	NA	-1.11	0.00	Down
POX05847	MFS toxin transporter	NA	1.35	0.00	Up
POX05850	Hypothetical protein	NA	-6.50	0.00	Down
POX05851	Hypothetical protein	NA	-2.37	0.00	Down
POX05852	Hypothetical protein	AA7	-4.84	0.00	Down

POX05853	Hypothetical protein	NA	-4.75	0.00	Down
POX05854	Hypothetical protein	NA	-8.49	0.00	Down
POX05855	Hypothetical protein	NA	-7.06	0.00	Down
POX05856	Hypothetical protein	NA	-6.67	0.00	Down
POX05857	Hypothetical protein	NA	-6.77	0.00	Down
POX05858	Hypothetical protein	NA	-6.70	0.00	Down
POX05859	Hypothetical protein	NA	-5.19	0.00	Down
POX05860	Hypothetical protein	NA	-7.67	0.00	Down
POX05861	Hypothetical protein	NA	-6.86	0.00	Down
POX05862	Hypothetical protein	NA	-8.73	0.00	Down
POX05863	Hypothetical protein	NA	-8.56	0.00	Down
POX05864	Hypothetical protein	NA	-7.50	0.00	Down
POX05865	Hypothetical protein	NA	-8.44	0.00	Down
POX05866	Hypothetical protein	NA	-8.73	0.00	Down
POX05867	Hypothetical protein	NA	-2.76	0.00	Down
POX05868	Hypothetical protein	NA	-5.50	0.00	Down
POX05869	Hypothetical protein	NA	-2.08	0.00	Down
POX05870	Hypothetical protein	NA	-5.54	0.00	Down
POX05871	Hypothetical protein	NA	-3.21	0.00	Down
POX05881	Hypothetical protein	NA	1.69	0.00	Up
POX05886	Hypothetical protein	NA	-2.39	0.00	Down
POX05887	Hypothetical protein	NA	-8.64	0.00	Down
POX05888	Hypothetical protein	NA	-7.99	0.00	Down
POX05892	Hypothetical protein	NA	-2.92	0.01	Down
POX05894	Hypothetical protein	NA	1.51	0.00	Up
POX05895	Hypothetical protein	NA	-2.36	0.03	Down
POX05896	Hypothetical protein	NA	-1.31	0.01	Down
POX05897	Sugar/inositol transporter	NA	-1.83	0.00	Down
POX05898	Hypothetical protein	NA	-3.97	0.00	Down
POX05905	Hypothetical protein	NA	-1.54	0.00	Down
POX05912	Hypothetical protein	NA	-2.05	0.00	Down
POX05913	Hypothetical protein	NA	-2.03	0.01	Down
POX05915	Cellodextrin transporter cdt-d	NA	-1.29	0.00	Down
POX05917	Hypothetical protein	NA	-1.20	0.00	Down
POX05918	Hypothetical protein	NA	4.44	0.00	Up
POX05924	Hypothetical protein	NA	-1.13	0.00	Down
POX05929	Hypothetical protein	NA	-2.36	0.00	Down
POX05944	Zn2Cys6; Fungal transcriptional regulatory protein, N-terminal	NA	1.35	0.00	Up
POX05957	Hypothetical protein	NA	2.18	0.00	Up
POX05959	Hypothetical protein	NA	1.24	0.00	Up

POX05964	Putative chitin synthase	NA	4.68	0.00	Up
POX05975	Sugar/inositol transporter	NA	4.33	0.00	Up
POX05976	Hypothetical protein	NA	3.75	0.00	Up
POX05977	Putative UDP-Xyl: (mannosyl) glucuronoxylomannan/galactoxylomannan beta-1,2-xylosyltransferase	GT90	1.06	0.00	Up
POX05980	Hypothetical protein	NA	1.49	0.00	Up
POX05989	Hypothetical protein	NA	-1.03	0.00	Down
POX05998	Hypothetical protein	NA	3.33	0.00	Up
POX06002	Hypothetical protein	NA	-3.13	0.00	Down
POX06016	Hypothetical protein	NA	-1.17	0.00	Down
POX06025	Hypothetical protein	NA	-1.69	0.00	Down
POX06030	Hypothetical protein	NA	-1.03	0.00	Down
POX06032	Hypothetical protein	NA	2.90	0.00	Up
POX06041	Hypothetical protein	NA	1.09	0.00	Up
POX06042	Putative beta-1,3-glucanosyltransferase	GH17	-1.33	0.00	Down
POX06047	Hypothetical protein	NA	-2.75	0.00	Down
POX06049	Hypothetical protein	NA	-1.60	0.00	Down
POX06051	Cellodextrin transporter cdt-c	NA	-1.38	0.00	Down
POX06052	Hypothetical protein	NA	1.15	0.00	Up
POX06054	Hypothetical protein	NA	1.08	0.00	Up
POX06057	Hypothetical protein	NA	-1.07	0.00	Down
POX06064	Sugar transporter, conserved site	NA	-2.99	0.00	Down
POX06066	Hypothetical protein	NA	2.62	0.00	Up
POX06069	Hypothetical protein	NA	-1.02	0.00	Down
POX06070	Zinc finger, NF-X1-type	NA	2.07	0.00	Up
POX06085	Hypothetical protein	NA	3.06	0.00	Up
POX06091	Hypothetical protein	NA	1.43	0.00	Up
POX06102	Hypothetical protein	NA	-6.84	0.00	Down
POX06112	Hypothetical protein	NA	-1.47	0.00	Down
POX06130	Hypothetical protein	NA	-1.21	0.00	Down
POX06145	Hypothetical protein	NA	-1.06	0.00	Down
POX06147	Endo-beta-1,4-glucanase Cel5A	CBM1;GH5	-2.03	0.00	Down
POX06151	Hypothetical protein	NA	-2.30	0.00	Down
POX06165	Hypothetical protein	NA	-2.64	0.00	Down
POX06167	Hypothetical protein	NA	1.45	0.00	Up
POX06168	Hypothetical protein	NA	2.87	0.00	Up
POX06169	Hypothetical protein	NA	2.24	0.00	Up
POX06170	Zn2Cys6; Fungal transcriptional regulatory protein, N-terminal	NA	1.18	0.00	Up
POX06171	Hypothetical protein	NA	1.14	0.00	Up

POX06178	Hypothetical protein	NA	-2.01	0.00	Down
POX06191	Hypothetical protein	NA	1.03	0.00	Up
POX06192	Zinc finger, GRF-type	NA	1.38	0.00	Up
POX06203	Hypothetical protein	CE10	1.23	0.00	Up
POX06204	Hypothetical protein	NA	-1.24	0.00	Down
POX06207	Hypothetical protein	NA	-1.46	0.00	Down
POX06223	Hypothetical protein	NA	1.52	0.00	Up
POX06228	Hypothetical protein	NA	1.40	0.00	Up
POX06238	Hypothetical protein	NA	1.13	0.00	Up
POX06249	Hypothetical protein	NA	-1.25	0.00	Down
POX06260	Hypothetical protein	NA	1.24	0.00	Up
POX06261	Hypothetical protein	NA	1.47	0.00	Up
POX06265	Hypothetical protein	NA	1.30	0.00	Up
POX06283	Hypothetical protein	NA	2.83	0.00	Up
POX06285	Hypothetical protein	NA	2.07	0.00	Up
POX06288	Hypothetical protein	NA	1.98	0.00	Up
POX06301	Putative alpha-mannosidase	GH47	-1.29	0.00	Down
POX06307	Hypothetical protein	NA	-1.57	0.00	Down
POX06308	Hypothetical protein	NA	-1.47	0.00	Down
POX06309	Hypothetical protein	NA	-1.90	0.00	Down
POX06312	Hypothetical protein	NA	6.28	0.00	Up
POX06317	Hypothetical protein	NA	-4.43	0.00	Down
POX06318	Hypothetical protein	NA	1.82	0.00	Up
POX06324	Putative exo-beta-1,3-glucanase	GH55	-1.24	0.00	Down
POX06326	Chitin binding domain-containing protein	NA	1.10	0.05	Up
POX06327	Hypothetical protein	NA	1.98	0.00	Up
POX06328	Hypothetical protein	NA	3.85	0.00	Up
POX06332	Zn2Cys6; Fungal transcriptional regulatory protein, N-terminal	NA	1.33	0.00	Up
POX06345	Hypothetical protein	NA	-2.42	0.00	Down
POX06354	Hypothetical protein	NA	3.48	0.00	Up
POX06357	Hypothetical protein	NA	1.09	0.00	Up
POX06362	Hypothetical protein	NA	-1.09	0.00	Down
POX06365	Hypothetical protein	NA	3.11	0.00	Up
POX06369	Hypothetical protein	NA	1.79	0.02	Up
POX06377	Negative transcriptional regulator	NA	2.12	0.00	Up
POX06380	SUN domain-containing protein	GH132	-1.46	0.00	Down
POX06388	Hypothetical protein	NA	1.98	0.00	Up
POX06391	Hypothetical protein	NA	-2.65	0.00	Down
POX06395	Hypothetical protein	NA	-2.41	0.00	Down
POX06396	Zinc finger, C2H2-type	NA	-1.63	0.00	Down

POX06398	Hypothetical protein	NA	-1.37	0.00	Down
POX06403	Hypothetical protein	NA	1.53	0.00	Up
POX06410	Hypothetical protein	NA	-4.25	0.00	Down
POX06413	Hypothetical protein	NA	-1.89	0.00	Down
POX06417	Hypothetical protein	NA	-1.03	0.00	Down
POX06426	Sugar/inositol transporter	NA	-1.82	0.00	Down
POX06428	Hypothetical protein	NA	-1.32	0.00	Down
POX06444	Hypothetical protein	NA	-2.01	0.00	Down
POX06450	Hypothetical protein	NA	-1.73	0.00	Down
POX06461	Hypothetical protein	NA	4.19	0.00	Up
POX06464	Hypothetical protein	NA	-1.23	0.00	Down
POX06466	Hypothetical protein	NA	-1.22	0.00	Down
POX06470	Hypothetical protein	NA	2.53	0.00	Up
POX06471	Hypothetical protein	NA	-1.48	0.00	Down
POX06474	Hypothetical protein	NA	-1.30	0.00	Down
POX06478	Hypothetical protein	NA	-1.68	0.00	Down
POX06486	Zinc finger, C2H2-type	NA	1.40	0.00	Up
POX06487	Hypothetical protein	NA	2.12	0.00	Up
POX06488	Hypothetical protein	NA	1.94	0.00	Up
POX06508	Hypothetical protein	NA	2.95	0.00	Up
POX06513	Hypothetical protein	NA	-2.34	0.00	Down
POX06519	Hypothetical protein	NA	3.05	0.00	Up
POX06520	Hypothetical protein	NA	3.01	0.00	Up
POX06525	Hypothetical protein	NA	-3.23	0.00	Down
POX06526	Hypothetical protein	NA	-3.12	0.00	Down
POX06527	Hypothetical protein	NA	-3.28	0.00	Down
POX06528	Hypothetical protein	NA	-4.38	0.00	Down
POX06530	Hypothetical protein	NA	1.31	0.00	Up
POX06531	Hypothetical protein	NA	1.30	0.00	Up
POX06534	Zinc finger, C2H2-type	NA	-6.76	0.00	Down
POX06539	Hypothetical protein	NA	-1.41	0.00	Down
POX06541	Hypothetical protein	NA	-1.77	0.00	Down
POX06563	Hypothetical protein	NA	1.67	0.00	Up
POX06565	Hypothetical protein	NA	1.91	0.00	Up
POX06569	Putative alpha-1,6-mannanase	GH76	2.50	0.00	Up
POX06570	Hypothetical protein	NA	1.95	0.01	Up
POX06571	beta-xylosidase	GH3	1.27	0.00	Up
POX06575	Hypothetical protein	NA	1.34	0.00	Up
POX06583	Hypothetical protein	NA	-1.67	0.00	Down
POX06590	Hypothetical protein	NA	1.41	0.00	Up

POX06595	Hypothetical protein	NA	-7.56	0.00	Down
POX06599	Putative alpha-L-arabinofuranosidase	CBM1;GH62	-3.94	0.00	Down
POX06600	Putative alpha-L-arabinofuranosidase	CBM1;GH43	-3.20	0.00	Down
POX06601	Putative endo-beta-1,4-xylanase	CBM1;GH30	-6.13	0.00	Down
POX06603	Hypothetical protein	NA	-1.04	0.00	Down
POX06604	Major facilitator, sugar transporter-like	NA	-1.77	0.00	Down
POX06605	Hypothetical protein	NA	-1.82	0.00	Down
POX06606	Hypothetical protein	NA	-1.90	0.00	Down
POX06607	Hypothetical protein	NA	-3.23	0.00	Down
POX06608	Hypothetical protein	NA	-2.48	0.00	Down
POX06611	Putative rhamnogalacturonan lyase	PL4	1.21	0.00	Up
POX06613	Hypothetical protein	NA	-1.58	0.00	Down
POX06626	TPA: COPII vesicles protein Yip3	NA	1.41	0.00	Up
POX06627	Hypothetical protein	NA	1.34	0.00	Up
POX06631	Hypothetical protein	NA	-1.42	0.00	Down
POX06633	Hypothetical protein	NA	-1.14	0.00	Down
POX06636	Hypothetical protein	NA	-1.56	0.00	Down
POX06641	Hypothetical protein	NA	-1.78	0.00	Down
POX06651	Hypothetical protein	NA	2.11	0.00	Up
POX06652	Hypothetical protein	NA	1.49	0.00	Up
POX06661	Hypothetical protein	NA	1.32	0.00	Up
POX06666	Basic-leucine zipper (bZIP) transcription factor	NA	1.65	0.00	Up
POX06683	Hypothetical protein	NA	3.50	0.00	Up
POX06686	Hypothetical protein	NA	1.35	0.00	Up
POX06700	Hypothetical protein	NA	1.12	0.00	Up
POX06712	Hypothetical protein	NA	2.10	0.00	Up
POX06715	Hypothetical protein	NA	1.54	0.00	Up
POX06725	Hypothetical protein	NA	-1.36	0.00	Down
POX06740	Hypothetical protein	NA	-1.19	0.00	Down
POX06743	Hypothetical protein	NA	-1.55	0.00	Down
POX06746	Hypothetical protein	NA	-1.43	0.00	Down
POX06748	Hypothetical protein	NA	-1.25	0.00	Down
POX06750	Hypothetical protein	NA	-1.59	0.00	Down
POX06751	Putative alpha-glucosidase	GH31	-1.73	0.00	Down
POX06754	Hypothetical protein	NA	-1.86	0.03	Down
POX06757	Hypothetical protein	NA	-1.15	0.00	Down
POX06758	Hypothetical protein	NA	-1.11	0.00	Down
POX06759	Zn2Cys6; Fungal specific transcription factor	NA	-1.36	0.00	Down
POX06780	Zn2Cys6; Fungal transcriptional regulatory protein, N-terminal	NA	1.54	0.00	Up
POX06783	Putative endo-beta-1,4-xylanase	CBM1;GH11	-1.95	0.00	Down

POX06784	Hypothetical protein	NA	1.14	0.00	Up
POX06791	Hypothetical protein	NA	-2.21	0.00	Down
POX06795	Hypothetical protein	NA	1.19	0.00	Up
POX06796	Hypothetical protein	NA	1.87	0.00	Up
POX06797	Hypothetical protein	NA	2.25	0.00	Up
POX06803	Hypothetical protein	NA	-1.31	0.02	Down
POX06804	Hypothetical protein	NA	-1.47	0.00	Down
POX06807	Hypothetical protein	NA	-1.02	0.00	Down
POX06815	Hypothetical protein	NA	1.12	0.00	Up
POX06819	Hypothetical protein	NA	1.27	0.00	Up
POX06820	Hypothetical protein	NA	-2.24	0.00	Down
POX06826	Hypothetical protein	NA	2.00	0.00	Up
POX06827	Hypothetical protein	NA	2.19	0.00	Up
POX06829	Hypothetical protein	NA	1.91	0.00	Up
POX06843	Hypothetical protein	NA	1.32	0.00	Up
POX06848	Hypothetical protein	NA	1.46	0.00	Up
POX06853	Hypothetical protein	NA	-1.12	0.00	Down
POX06858	Hypothetical protein	NA	1.32	0.00	Up
POX06862	Hypothetical protein	NA	1.79	0.00	Up
POX06865	Centromere protein B, DNA-binding region	NA	-1.57	0.01	Down
POX06870	Hypothetical protein	NA	1.54	0.00	Up
POX06872	Zn2Cys6; Fungal specific transcription factor	NA	1.09	0.00	Up
POX06875	Hypothetical protein	NA	-1.34	0.00	Down
POX06877	Hypothetical protein	NA	2.42	0.00	Up
POX06884	Hypothetical protein	NA	-1.31	0.00	Down
POX06888	Hypothetical protein	CE10	-1.10	0.00	Down
POX06889	Hypothetical protein	NA	1.98	0.00	Up
POX06899	Putative exo-beta-1, 3-glucanase	GH5	-2.06	0.00	Down
POX06900	Putative beta-glucuronidase	GH2	-2.35	0.00	Down
POX06919	Hypothetical protein	NA	1.94	0.00	Up
POX06921	Hypothetical protein	NA	-3.32	0.00	Down
POX06922	Hypothetical protein	NA	-1.08	0.00	Down
POX06925	Zn2Cys6; Fungal transcriptional regulatory protein, N-terminal	NA	1.40	0.00	Up
POX06926	Hypothetical protein	NA	-1.44	0.00	Down
POX06936	Hypothetical protein	NA	-1.13	0.00	Down
POX06940	Hypothetical protein	NA	-1.07	0.00	Down
POX06951	Hypothetical protein	NA	-4.62	0.00	Down
POX06972	Hypothetical protein	NA	-1.32	0.00	Down
POX06976	Hypothetical protein	NA	-1.62	0.00	Down
POX06977	Hypothetical protein	NA	-1.78	0.00	Down

POX06979	Hypothetical protein	NA	1.13	0.00	Up
POX06981	Hypothetical protein	NA	1.19	0.00	Up
POX06983	Endo-beta-1,4-glucanase	GH12	-3.02	0.00	Down
POX06986	Hypothetical protein	NA	-2.49	0.00	Down
POX07020	Hypothetical protein	NA	1.16	0.00	Up
POX07024	Hypothetical protein	NA	3.53	0.00	Up
POX07029	Hypothetical protein	NA	1.65	0.00	Up
POX07040	Hypothetical protein	NA	-1.59	0.00	Down
POX07046	Hypothetical protein	GT41	1.45	0.00	Up
POX07060	Hypothetical protein	NA	1.44	0.00	Up
POX07083	Putative beta-1, 3-1, 4-glucanase	GH16	-2.85	0.00	Down
POX07099	Myb	NA	-3.90	0.00	Down
POX07104	Putative alpha-xylosidase	GH31	-1.07	0.00	Down
POX07107	Hypothetical protein	NA	-1.49	0.00	Down
POX07113	Zinc finger, C2H2-type	NA	3.58	0.00	Up
POX07116	Hypothetical protein	NA	1.76	0.00	Up
POX07117	Hypothetical protein	NA	3.03	0.00	Up
POX07123	Hypothetical protein	NA	-4.57	0.00	Down
POX07134	Hypothetical protein	NA	1.37	0.00	Up
POX07135	Hypothetical protein	NA	-3.43	0.00	Down
POX07145	Putative chitinase	GH18	1.22	0.00	Up
POX07147	Hypothetical protein	NA	1.03	0.00	Up
POX07157	Hypothetical protein	NA	-1.33	0.00	Down
POX07173	Hypothetical protein	NA	-1.34	0.00	Down
POX07195	Hypothetical protein	NA	-1.19	0.00	Down
POX07202	Hypothetical protein	NA	-1.04	0.00	Down
POX07203	Hypothetical protein	NA	-1.60	0.00	Down
POX07208	Hypothetical protein	NA	2.71	0.00	Up
POX07209	Sugar/inositol transporter	NA	1.93	0.04	Up
POX07211	Hypothetical protein	NA	1.25	0.00	Up
POX07221	Hypothetical protein	NA	1.19	0.00	Up
POX07227	Sugar/inositol transporter	NA	2.17	0.00	Up
POX07232	Hypothetical protein	NA	-3.12	0.00	Down
POX07238	Hypothetical protein	NA	-8.01	0.00	Down
POX07240	Hypothetical protein	NA	-1.10	0.00	Down
POX07251	Hypothetical protein	NA	2.36	0.00	Up
POX07254	Carbon catabolite repressor CreA	NA	-1.12	0.00	Down
POX07268	putative cell-wall beta-1, 6-glucan active enzyme	NA	-1.76	0.00	Down
POX07269	Hypothetical protein	NA	-4.38	0.00	Down
POX07270	Hypothetical protein	NA	-4.43	0.00	Down

POX07271	Hypothetical protein	NA	1.52	0.00	Up
POX07279	Hypothetical protein	NA	-2.86	0.00	Down
POX07291	Hypothetical protein	NA	-1.18	0.00	Down
POX07304	Putative polygalacturonase	GH28	-1.07	0.00	Down
POX07325	Hypothetical protein	NA	1.08	0.00	Up
POX07327	Hypothetical protein	CE10	2.07	0.00	Up
POX07328	Hypothetical protein	NA	1.41	0.00	Up
POX07337	Hypothetical protein	NA	1.05	0.00	Up
POX07339	Hypothetical protein	NA	3.78	0.00	Up
POX07340	Hypothetical protein	NA	2.37	0.00	Up
POX07341	Hypothetical protein	NA	1.42	0.00	Up
POX07342	Hypothetical protein	AA8	2.07	0.03	Up
POX07360	Hypothetical protein	NA	-1.13	0.00	Down
POX07363	Hypothetical protein	NA	-3.58	0.00	Down
POX07365	Hypothetical protein	NA	3.11	0.01	Up
POX07366	Hypothetical protein	NA	1.99	0.00	Up
POX07367	Hypothetical protein	NA	2.68	0.00	Up
POX07368	Hypothetical protein	NA	1.32	0.04	Up
POX07380	Putative lysozyme	GH25	-1.01	0.00	Down
POX07386	Hypothetical protein	NA	1.50	0.00	Up
POX07388	Hypothetical protein	NA	-4.78	0.00	Down
POX07389	Hypothetical protein	NA	-7.25	0.00	Down
POX07390	Hypothetical protein	NA	-7.33	0.00	Down
POX07391	Hypothetical protein	NA	-5.36	0.00	Down
POX07392	Hypothetical protein	NA	-6.64	0.00	Down
POX07397	Carbohydrate binding domain-containing protein	NA	1.74	0.00	Up
POX07398	Hypothetical protein	NA	2.44	0.00	Up
POX07399	Putative UDP-Glc: sterol glucosyltransferase	GT1	4.75	0.00	Up
POX07400	Hypothetical protein	NA	3.48	0.00	Up
POX07401	Hypothetical protein	NA	-2.03	0.00	Down
POX07405	Hypothetical protein	NA	-1.21	0.00	Down
POX07408	Hypothetical protein	NA	-1.27	0.00	Down
POX07411	Hypothetical protein	NA	1.74	0.04	Up
POX07414	Centromere protein B, DNA-binding region	NA	1.18	0.01	Up
POX07419	Hypothetical protein	NA	-2.36	0.00	Down
POX07420	Hypothetical protein	NA	-2.88	0.00	Down
POX07422	Hypothetical protein	NA	1.05	0.00	Up
POX07432	Hypothetical protein	NA	-2.61	0.00	Down
POX07438	Hypothetical protein	NA	-1.28	0.00	Down
POX07451	Sugar/inositol transporter	NA	-1.39	0.00	Down

POX07458	Hypothetical protein	NA	2.17	0.00	Up
POX07466	Hypothetical protein	NA	-2.00	0.00	Down
POX07475	Hypothetical protein	NA	-1.08	0.00	Down
POX07477	Hypothetical protein	NA	1.12	0.00	Up
POX07511	Hypothetical protein	NA	-2.82	0.00	Down
POX07516	Hypothetical protein	NA	-1.00	0.00	Down
POX07519	Basic-leucine zipper (bZIP) transcription factor	NA	-1.40	0.00	Down
POX07528	Hypothetical protein	NA	2.18	0.00	Up
POX07531	Hypothetical protein	NA	1.25	0.00	Up
POX07532	Hypothetical protein	NA	1.64	0.01	Up
POX07534	Putative beta-1, 3-glucanosyltransferase	GH17	-1.66	0.00	Down
POX07538	Hypothetical protein	NA	-2.69	0.01	Down
POX07539	Hypothetical protein	NA	2.19	0.00	Up
POX07541	Sugar/inositol transporter	NA	1.02	0.00	Up
POX07542	Hypothetical protein	NA	2.13	0.03	Up
POX07553	Putative chitin synthase	GT2	-1.13	0.00	Down
POX07556	Hypothetical protein	NA	1.62	0.00	Up
POX07557	Hypothetical protein	NA	1.14	0.00	Up
POX07560	Hypothetical protein	NA	1.50	0.00	Up
POX07561	Hypothetical protein	NA	4.29	0.00	Up
POX07562	Hypothetical protein	NA	3.84	0.00	Up
POX07564	Hypothetical protein	NA	2.21	0.00	Up
POX07565	Hypothetical protein	NA	1.21	0.00	Up
POX07573	Putative beta-N-acetylhexosaminidase	GH3	-1.07	0.00	Down
POX07576	Sugar/inositol transporter	NA	-4.41	0.00	Down
POX07577	Hypothetical protein	NA	1.91	0.00	Up
POX07584	Hypothetical protein	NA	2.38	0.00	Up
POX07586	Hypothetical protein	NA	-1.58	0.00	Down
POX07592	Hypothetical protein	NA	2.32	0.00	Up
POX07602	Hypothetical protein	NA	1.58	0.00	Up
POX07610	Hypothetical protein	NA	1.31	0.00	Up
POX07618	Hypothetical protein	NA	2.91	0.00	Up
POX07620	Hypothetical protein	NA	-1.63	0.00	Down
POX07626	Hypothetical protein	NA	1.62	0.00	Up
POX07627	Hypothetical protein	NA	2.27	0.00	Up
POX07630	Hypothetical protein	NA	2.86	0.00	Up
POX07633	Hypothetical protein	NA	1.52	0.00	Up
POX07634	Hypothetical protein	NA	1.38	0.00	Up
POX07636	Hypothetical protein	NA	1.35	0.00	Up
POX07638	Hypothetical protein	NA	-1.11	0.00	Down

POX07641	Putative alpha-1, 3-glucanase	CBM24;GH71	-2.30	0.00	Down
POX07644	Hypothetical protein	NA	-1.12	0.00	Down
POX07646	Hypothetical protein	NA	-2.55	0.00	Down
POX07649	Hypothetical protein	NA	-1.55	0.00	Down
POX07666	Hypothetical protein	NA	1.09	0.00	Up
POX07668	Hypothetical protein	NA	-1.08	0.00	Down
POX07677	Hypothetical protein	NA	-2.00	0.00	Down
POX07690	Hypothetical protein	NA	1.65	0.00	Up
POX07718	Hypothetical protein	NA	3.01	0.00	Up
POX07722	Hypothetical protein	NA	-1.83	0.00	Down
POX07735	Hypothetical protein	NA	2.37	0.00	Up
POX07738	Hypothetical protein	NA	-1.06	0.00	Down
POX07750	Hypothetical protein	NA	2.62	0.02	Up
POX07751	Hypothetical protein	NA	7.36	0.00	Up
POX07754	Hypothetical protein	NA	3.11	0.00	Up
POX07755	Zn2Cys6; Fungal transcriptional regulatory protein, N-terminal	NA	1.23	0.03	Up
POX07757	Hypothetical protein	NA	4.85	0.00	Up
POX07762	Hypothetical protein	NA	1.21	0.00	Up
POX07771	Hypothetical protein	NA	1.91	0.00	Up
POX07772	Hypothetical protein	NA	-2.75	0.00	Down
POX07775	Sugar/inositol transporter	NA	1.09	0.01	Up
POX07778	Hypothetical protein	NA	1.04	0.00	Up
POX07783	Sugar transporter, conserved site	NA	-1.23	0.01	Down
POX07785	Hypothetical protein	NA	-2.31	0.00	Down
POX07793	Hypothetical protein	NA	1.43	0.00	Up
POX07797	Hypothetical protein	NA	-1.72	0.00	Down
POX07801	Hypothetical protein	NA	1.38	0.04	Up
POX07804	Hypothetical protein	NA	-2.65	0.00	Down
POX07805	Hypothetical protein	NA	2.28	0.00	Up
POX07819	Hypothetical protein	NA	-5.30	0.00	Down
POX07820	Ecm33 domain-containing protein	NA	-1.22	0.00	Down
POX07830	Hypothetical protein	NA	-1.87	0.00	Down
POX07832	Hypothetical protein	NA	-2.01	0.00	Down
POX07833	peptidoglycan binding domain-containing protein	NA	-2.47	0.00	Down
POX07842	Hypothetical protein	NA	-2.67	0.00	Down
POX07847	Hypothetical protein	NA	-1.75	0.00	Down
POX07852	Hypothetical protein	NA	-1.28	0.00	Down
POX07862	Hypothetical protein	NA	-1.93	0.00	Down
POX07863	Hypothetical protein	NA	-1.10	0.00	Down
POX07868	Hypothetical protein	NA	-1.84	0.00	Down

POX07880	Hypothetical protein	NA	1.39	0.00	Up
POX07883	Hypothetical protein	NA	-1.15	0.00	Down
POX07886	Hypothetical protein	NA	3.63	0.00	Up
POX07888	Hypothetical protein	NA	-4.18	0.00	Down
POX07889	Hypothetical protein	NA	-1.95	0.00	Down
POX07896	Hypothetical protein	NA	2.34	0.00	Up
POX07899	Hypothetical protein	NA	-5.28	0.00	Down
POX07907	Hypothetical protein	AA3;AA8	4.61	0.00	Up
POX07908	Hypothetical protein	AA7	2.76	0.00	Up
POX07923	Hypothetical protein	NA	-1.89	0.00	Down
POX07924	Hypothetical protein	NA	-1.81	0.01	Down
POX07928	Hypothetical protein	NA	-1.19	0.00	Down
POX07934	Zinc finger, GATA-type	NA	-1.30	0.00	Down
POX07935	Hypothetical protein	NA	-1.11	0.00	Down
POX07938	Zn2Cys6; Fungal transcriptional regulatory protein, N-terminal	NA	-1.70	0.00	Down
POX07951	Hypothetical protein	NA	1.75	0.00	Up
POX07954	Hypothetical protein	NA	-5.38	0.00	Down
POX07962	Hypothetical protein	NA	-1.59	0.00	Down
POX07963	Putative beta-glucosidase	GH3	-2.60	0.00	Down
POX07971	Putative chitinase	GH18	-1.39	0.00	Down
POX08002	Hypothetical protein	NA	1.17	0.01	Up
POX08009	Mating-type protein MAT alpha 1	NA	6.79	0.00	Up
POX08010	Hypothetical protein	NA	1.29	0.00	Up
POX08016	Hypothetical protein	NA	1.01	0.00	Up
POX08029	Hypothetical protein	NA	2.29	0.00	Up
POX08030	Hypothetical protein	NA	2.34	0.00	Up
POX08032	Hypothetical protein	NA	-3.42	0.00	Down
POX08034	Putative endo-alpha-1, 4-polygalactosaminidase	GH114	1.02	0.00	Up
POX08039	Putative polysialyltransferase	GT4	-1.73	0.00	Down
POX08041	Zn2Cys6; Fungal transcriptional regulatory protein, N-terminal	NA	1.08	0.00	Up
POX08045	Hypothetical protein	NA	-1.02	0.00	Down
POX08046	Hypothetical protein	NA	-1.65	0.00	Down
POX08048	Hypothetical protein	NA	-2.65	0.00	Down
POX08049	Hypothetical protein	NA	-2.52	0.00	Down
POX08065	Hypothetical protein	NA	-2.06	0.04	Down
POX08066	Hypothetical protein	NA	-1.14	0.00	Down
POX08068	Hypothetical protein	NA	1.27	0.00	Up
POX08073	Hypothetical protein	NA	2.80	0.00	Up
POX08079	Hypothetical protein	NA	1.17	0.00	Up
POX08080	Putative chitin glucanosyltransferase	GH16	-1.15	0.00	Down

POX08085	Hypothetical protein	NA	-1.29	0.00	Down
POX08089	Hypothetical protein	NA	2.65	0.00	Up
POX08091	Hypothetical protein	NA	1.45	0.00	Up
POX08092	Hypothetical protein	CE10	2.91	0.00	Up
POX08095	Sugar/inositol transporter	NA	-1.25	0.00	Down
POX08105	Hypothetical protein	NA	1.53	0.00	Up
POX08107	Hypothetical protein	NA	-6.77	0.00	Down
POX08108	Hypothetical protein	NA	-7.53	0.00	Down
POX08109	Hypothetical protein	NA	-5.74	0.00	Down
POX08110	Hypothetical protein	NA	-1.61	0.00	Down
POX08114	Hypothetical protein	NA	3.62	0.00	Up
POX08115	Hypothetical protein	NA	2.00	0.00	Up
POX08117	Hypothetical protein	NA	-1.32	0.00	Down
POX08135	Hypothetical protein	NA	1.37	0.00	Up
POX08136	Hypothetical protein	NA	2.28	0.00	Up
POX08137	Hypothetical protein	NA	2.00	0.00	Up
POX08140	Putative alpha-L-arabinofuranosidase	GH43	2.36	0.00	Up
POX08141	Hypothetical protein	NA	1.62	0.00	Up
POX08153	Hypothetical protein	NA	-1.67	0.00	Down
POX08154	Hypothetical protein	NA	-5.22	0.00	Down
POX08165	Hypothetical protein	NA	1.10	0.00	Up
POX08171	Putative chitinase	GH18	-5.09	0.00	Down
POX08175	Hypothetical protein	AA7	1.04	0.00	Up
POX08182	Hypothetical protein	NA	3.75	0.00	Up
POX08183	Hypothetical protein	NA	5.67	0.00	Up
POX08190	Hypothetical protein	NA	-3.20	0.00	Down
POX08194	Hypothetical protein	NA	1.15	0.00	Up
POX08208	Hypothetical protein	NA	-2.05	0.00	Down
POX08214	Hypothetical protein	NA	1.84	0.00	Up
POX08245	Hypothetical protein	CE10;CE1	1.17	0.00	Up
POX08268	Hypothetical protein	NA	3.24	0.00	Up
POX08273	Hypothetical protein	NA	7.02	0.00	Up
POX08280	Hypothetical protein	NA	1.15	0.00	Up
POX08282	Hypothetical protein	NA	1.65	0.00	Up
POX08284	Hypothetical protein	NA	-1.45	0.00	Down
POX08290	Hypothetical protein	NA	1.55	0.00	Up
POX08296	Hypothetical protein	NA	-2.17	0.00	Down
POX08302	Hypothetical protein	NA	-1.29	0.00	Down
POX08303	Hypothetical protein	NA	1.07	0.00	Up
POX08305	Hypothetical protein	NA	-5.48	0.00	Down

POX08306	Hypothetical protein	NA	-1.03	0.00	Down
POX08310	Hypothetical protein	NA	-1.14	0.00	Down
POX08311	Hypothetical protein	NA	-2.66	0.00	Down
POX08316	Hypothetical protein	NA	-1.27	0.00	Down
POX08320	Hypothetical protein	NA	1.78	0.00	Up
POX08330	Hypothetical protein	NA	1.06	0.00	Up
POX08339	Hypothetical protein	NA	-6.65	0.00	Down
POX08341	Hypothetical protein	NA	-1.78	0.00	Down
POX08345	Hypothetical protein	NA	-1.71	0.00	Down
POX08346	Hypothetical protein	NA	2.33	0.00	Up
POX08355	Hypothetical protein	NA	-2.95	0.01	Down
POX08356	Hypothetical protein	NA	-1.06	0.00	Down
POX08359	Hypothetical protein	NA	-1.97	0.00	Down
POX08373	Hypothetical protein	NA	1.44	0.00	Up
POX08374	Hypothetical protein	NA	2.80	0.00	Up
POX08375	Cys2-His2 zinc finger transcription factor AceA	NA	1.28	0.00	Up
POX08376	Hypothetical protein	NA	1.57	0.00	Up
POX08379	Sugar/inositol transporter	NA	1.01	0.00	Up
POX08381	Hypothetical protein	NA	-3.93	0.00	Down
POX08383	Hypothetical protein	NA	1.08	0.00	Up
POX08384	Hypothetical protein	NA	1.09	0.01	Up
POX08386	Hypothetical protein	NA	-2.96	0.00	Down
POX08410	Hypothetical protein	NA	2.07	0.00	Up
POX08415	Zinc finger, GATA-type	NA	-1.67	0.00	Down
POX08422	Hypothetical protein	NA	-1.05	0.00	Down
POX08439	Hypothetical protein	NA	-1.05	0.00	Down
POX08441	Hypothetical protein	NA	1.12	0.00	Up
POX08443	Hypothetical protein	NA	-1.51	0.00	Down
POX08444	Hypothetical protein	NA	-2.52	0.00	Down
POX08445	Hypothetical protein	NA	-1.35	0.05	Down
POX08446	Hypothetical protein	NA	1.91	0.00	Up
POX08447	Hypothetical protein	NA	3.29	0.00	Up
POX08451	Hypothetical protein	NA	-1.23	0.00	Down
POX08452	Hypothetical protein	NA	-1.84	0.00	Down
POX08454	Hypothetical protein	NA	-1.17	0.00	Down
POX08456	Hypothetical protein	NA	-1.36	0.00	Down
POX08459	Hypothetical protein	NA	1.12	0.00	Up
POX08461	Hypothetical protein	NA	2.67	0.00	Up
POX08473	Hypothetical protein	NA	2.23	0.00	Up
POX08474	Hypothetical protein	NA	-2.40	0.00	Down

POX08480	Hypothetical protein	NA	1.37	0.00	Up
POX08482	Hypothetical protein	NA	-1.05	0.00	Down
POX08483	Putative alpha-N-acetylglucosaminidase	GH89	1.18	0.00	Up
POX08484	Endo-beta-1,4-xylanase	CBM1;GH11	-1.79	0.00	Down
POX08485	Swollenin	CBM1	-1.83	0.00	Down
POX08486	Hypothetical protein	NA	-1.28	0.00	Down
POX08487	Hypothetical protein	NA	1.16	0.00	Up
POX08496	Hypothetical protein	NA	2.04	0.02	Up
POX08497	Hypothetical protein	NA	1.97	0.00	Up
POX08498	Hypothetical protein	NA	2.29	0.00	Up
POX08506	Hypothetical protein	NA	-1.32	0.00	Down
POX08519	Hypothetical protein	NA	1.06	0.00	Up
POX08520	Hypothetical protein	NA	2.21	0.00	Up
POX08529	Hypothetical protein	NA	-1.31	0.00	Down
POX08562	Hypothetical protein	NA	-1.85	0.00	Down
POX08572	Hypothetical protein	NA	1.33	0.00	Up
POX08578	Hypothetical protein	NA	-1.13	0.00	Down
POX08579	Hypothetical protein	NA	-6.38	0.00	Down
POX08583	Hypothetical protein	NA	-1.18	0.00	Down
POX08596	Hypothetical protein	NA	-5.29	0.00	Down
POX08598	Hypothetical protein	NA	1.63	0.04	Up
POX08603	Hypothetical protein	NA	4.57	0.00	Up
POX08609	Putative exo-beta-1, 3-glucanase	GH55	2.27	0.00	Up
POX08610	Hypothetical protein	NA	1.37	0.00	Up
POX08611	Hypothetical protein	NA	1.17	0.00	Up
POX08622	Hypothetical protein	NA	1.04	0.00	Up
POX08627	Hypothetical protein	NA	-2.90	0.00	Down
POX08633	Hypothetical protein	NA	-3.69	0.00	Down
POX08636	Hypothetical protein	NA	-2.02	0.00	Down
POX08646	Hypothetical protein	NA	-1.28	0.00	Down
POX08650	Hypothetical protein	NA	-1.34	0.00	Down
POX08652	Hypothetical protein	NA	-2.01	0.00	Down
POX08654	Hypothetical protein	NA	-1.77	0.00	Down
POX08656	Sugar transporter, conserved site	NA	-1.38	0.02	Down
POX08658	Sugar/inositol transporter	NA	3.02	0.00	Up
POX08673	Hypothetical protein	NA	-1.03	0.00	Down
POX08674	Hypothetical protein	NA	1.73	0.00	Up
POX08677	Hypothetical protein	NA	-2.17	0.00	Down
POX08682	Hypothetical protein	NA	-6.06	0.00	Down
POX08683	Hypothetical protein	NA	-5.88	0.00	Down

POX08684	Hypothetical protein	NA	-7.47	0.00	Down
POX08685	Hypothetical protein	NA	-7.77	0.00	Down
POX08688	Hypothetical protein	NA	1.01	0.01	Up
POX08689	Hypothetical protein	NA	2.77	0.00	Up
POX08693	Hypothetical protein	NA	2.91	0.00	Up
POX08694	Hypothetical protein	AA1	-6.14	0.00	Down
POX08695	Hypothetical protein	NA	-1.49	0.02	Down
POX08696	Hypothetical protein	NA	-8.68	0.00	Down
POX08698	Hypothetical protein	NA	-7.02	0.00	Down
POX08699	Hypothetical protein	NA	-4.71	0.00	Down
POX08700	Hypothetical protein	NA	-6.34	0.00	Down
POX08702	Winged helix repressor DNA-binding	NA	-4.80	0.00	Down
POX08703	Hypothetical protein	NA	-6.29	0.00	Down
POX08705	Hypothetical protein	NA	5.04	0.00	Up
POX08706	Hypothetical protein	NA	2.63	0.00	Up
POX08729	Hypothetical protein	NA	2.78	0.00	Up
POX08734	Hypothetical protein	NA	-1.10	0.00	Down
POX08735	Hypothetical protein	NA	-5.89	0.00	Down
POX08741	Hypothetical protein	NA	-2.95	0.01	Down
POX08742	Hypothetical protein	NA	-4.00	0.00	Down
POX08743	Hypothetical protein	NA	4.64	0.00	Up
POX08745	Hypothetical protein	NA	2.30	0.00	Up
POX08746	Hypothetical protein	NA	2.58	0.00	Up
POX08752	Zn2Cys6; Fungal transcriptional regulatory protein, N-terminal	NA	1.21	0.00	Up
POX08753	Hypothetical protein	NA	-5.89	0.00	Down
POX08757	Hypothetical protein	NA	7.44	0.00	Up
POX08758	Hypothetical protein	AA3;AA8	-3.94	0.00	Down
POX08766	Hypothetical protein	NA	-3.79	0.00	Down
POX08767	Hypothetical protein	NA	-1.94	0.00	Down
POX08769	Hypothetical protein	NA	2.72	0.02	Up
POX08777	Hypothetical protein	NA	2.46	0.04	Up
POX08779	Hypothetical protein	NA	1.87	0.00	Up
POX08782	Hypothetical protein	NA	-1.20	0.00	Down
POX08783	Sugar/inositol transporter	NA	1.63	0.00	Up
POX08784	Hypothetical protein	NA	1.67	0.05	Up
POX08788	Hypothetical protein	NA	-1.76	0.00	Down
POX08801	Hypothetical protein	NA	-1.10	0.00	Down
POX08805	Zinc finger, C2H2-type	NA	1.41	0.00	Up
POX08806	Hypothetical protein	NA	-2.49	0.00	Down
POX08807	Hypothetical protein	NA	-2.72	0.00	Down

POX08810	Hypothetical protein	NA	-1.47	0.00	Down
POX08813	Starch binding domain- and chitin binding domain-containing protein	CBM20;AA13	1.44	0.00	Up
POX08814	Hypothetical protein	NA	-2.34	0.00	Down
POX08824	Hypothetical protein	NA	2.60	0.00	Up
POX08826	Hypothetical protein	NA	2.89	0.00	Up
POX08827	Putative endo-beta-1, 3-glucanase	GH55	-3.71	0.00	Down
POX08828	Hypothetical protein	NA	-5.85	0.00	Down
POX08829	Hypothetical protein	NA	-3.12	0.00	Down
POX08830	Hypothetical protein	NA	1.08	0.00	Up
POX08831	Hypothetical protein	NA	2.03	0.00	Up
POX08833	Hypothetical protein	NA	1.15	0.00	Up
POX08836	Hypothetical protein	NA	-1.58	0.00	Down
POX08849	Hypothetical protein	NA	2.38	0.00	Up
POX08852	Hypothetical protein	NA	2.05	0.00	Up
POX08853	Hypothetical protein	NA	4.09	0.00	Up
POX08854	Hypothetical protein	NA	1.22	0.00	Up
POX08861	Putative acetyl xylan esterase	CBM1;CE2	-1.07	0.00	Down
POX08873	Hypothetical protein	NA	2.29	0.00	Up
POX08876	Hypothetical protein	CE9	-2.05	0.00	Down
POX08881	Hypothetical protein	GH105;GH76	-1.65	0.00	Down
POX08882	Putative beta-glucosidase	GH1	-1.74	0.00	Down
POX08885	Hypothetical protein	NA	-1.23	0.00	Down
POX08889	Hypothetical protein	NA	2.50	0.00	Up
POX08890	Hypothetical protein	NA	6.22	0.00	Up
POX08895	Hypothetical protein	GT90	-3.79	0.00	Down
POX08897	Putative cellulose monooxygenase	CBM1;AA9	-2.30	0.00	Down
POX08899	Hypothetical protein	NA	2.14	0.00	Up
POX08900	Zn2Cys6; Fungal transcriptional regulatory protein, N-terminal	NA	1.05	0.00	Up
POX08902	Putative alpha-1, 6-mannanase	GH76	6.81	0.00	Up
POX08903	Putative chitosanase	GH75	1.54	0.00	Up
POX08906	Hypothetical protein	NA	2.63	0.00	Up
POX08910	Homeodomain-like	NA	1.13	0.00	Up
POX08912	Hypothetical protein	NA	-4.50	0.00	Down
POX08936	Hypothetical protein	NA	2.80	0.01	Up
POX08948	Hypothetical protein	NA	1.31	0.01	Up
POX08951	Hypothetical protein	NA	-1.91	0.00	Down
POX08963	Hypothetical protein	NA	1.13	0.00	Up
POX08967	Putative alpha-L-rhamnosidase	GH78	4.40	0.00	Up
POX08970	Hypothetical protein	NA	1.38	0.00	Up

POX08971	Hypothetical protein	NA	1.30	0.00	Up
POX08972	Hypothetical protein	NA	-1.18	0.00	Down
POX08987	Hypothetical protein	NA	3.01	0.00	Up
POX08990	Putative endo-beta-1,4-xylanase	GH10	-1.08	0.02	Down
POX08997	Hypothetical protein	NA	-1.24	0.05	Down
POX08998	Hypothetical protein	NA	-1.43	0.00	Down
POX09003	Hypothetical protein	NA	-1.01	0.00	Down
POX09005	Hypothetical protein	NA	1.10	0.00	Up
POX09019	Hypothetical protein	NA	-1.01	0.00	Down
POX09026	Hypothetical protein	NA	1.19	0.00	Up
POX09030	Hypothetical protein	NA	1.17	0.00	Up
POX09033	Hypothetical protein	NA	-4.53	0.00	Down
POX09035	Hypothetical protein	NA	1.25	0.00	Up
POX09039	Hypothetical protein	NA	1.18	0.00	Up
POX09085	Putative alpha-galactosidase	CBM1;GH27;GH36	-4.25	0.00	Down
POX09088	Zn2Cys6; Fungal transcriptional regulatory protein, N-terminal	NA	-2.03	0.00	Down
POX09096	Hypothetical protein	NA	-1.08	0.00	Down
POX09101	Hypothetical protein	NA	-2.01	0.00	Down
POX09104	Hypothetical protein	NA	-1.46	0.00	Down
POX09105	Hypothetical protein	NA	-3.03	0.00	Down
POX09106	Hypothetical protein	NA	-2.92	0.01	Down
POX09109	Hypothetical protein	NA	-2.92	0.00	Down
POX09110	Putative alpha-1, 3-glucanase	CBM24;GH71	-3.67	0.00	Down
POX09111	Hypothetical protein	NA	-1.17	0.01	Down
POX09114	Hypothetical protein	NA	-2.18	0.00	Down
POX09116	Winged helix repressor DNA-binding	NA	-1.37	0.01	Down
POX09117	Hypothetical protein	NA	-2.25	0.00	Down
POX09118	Hypothetical protein	NA	2.22	0.04	Up
POX09119	Hypothetical protein	NA	1.63	0.00	Up
POX09123	Hypothetical protein	NA	2.29	0.05	Up
POX09136	Hypothetical protein	NA	-1.01	0.00	Down
POX09137	Putative feruloyl esterase	CBM1;CE1	-3.68	0.00	Down
POX09138	Hypothetical protein	NA	-1.11	0.00	Down
POX09141	Hypothetical protein	NA	1.19	0.00	Up
POX09149	Zn2Cys6; Fungal transcriptional regulatory protein, N-terminal	NA	1.09	0.00	Up
POX09152	Hypothetical protein	NA	4.17	0.00	Up
POX09164	Hypothetical protein	NA	-1.37	0.01	Down
POX09166	Hypothetical protein	NA	2.15	0.00	Up
POX09169	Hypothetical protein	NA	-2.72	0.00	Down
POX09171	Hypothetical protein	NA	-1.19	0.05	Down

POX09172	Hypothetical protein	NA	1.70	0.00	Up
POX09181	Hypothetical protein	NA	-1.29	0.00	Down
POX09182	Hypothetical protein	NA	1.11	0.01	Up
POX09183	Hypothetical protein	NA	1.56	0.00	Up
POX09186	Hypothetical protein	NA	2.16	0.00	Up
POX09187	High mobility group box	NA	-1.14	0.00	Down
POX09195	Hypothetical protein	NA	1.75	0.00	Up
POX09210	Hypothetical protein	NA	3.22	0.00	Up
POX09213	Hypothetical protein	NA	3.04	0.00	Up
POX09218	Hypothetical protein	NA	-1.24	0.00	Down
POX09219	Hypothetical protein	NA	-1.26	0.00	Down
POX09222	Hypothetical protein	NA	-2.61	0.00	Down
POX09228	Hypothetical protein	NA	2.03	0.03	Up
POX09229	Hypothetical protein	NA	1.59	0.00	Up
POX09230	Hypothetical protein	NA	3.02	0.01	Up
POX09234	Hypothetical protein	NA	2.67	0.00	Up
POX09239	Hypothetical protein	NA	1.04	0.00	Up
POX09240	Hypothetical protein	NA	-3.08	0.00	Down
POX09258	Hypothetical protein	GH127	-2.65	0.00	Down
POX09260	Hypothetical protein	AA7	4.79	0.00	Up
POX09265	Hypothetical protein	NA	2.91	0.00	Up
POX09266	Hypothetical protein	NA	1.11	0.00	Up
POX09267	Hypothetical protein	NA	1.35	0.00	Up
POX09277	Hypothetical protein	NA	-1.06	0.00	Down
POX09289	Hypothetical protein	NA	-1.08	0.00	Down
POX09297	Hypothetical protein	NA	-1.18	0.00	Down
POX09299	Hypothetical protein	NA	1.57	0.00	Up
POX09313	Hypothetical protein	NA	1.23	0.01	Up
POX09318	Zn2Cys6; Fungal specific transcription factor	NA	3.02	0.00	Up
POX09319	Hypothetical protein	NA	3.68	0.00	Up
POX09320	Hypothetical protein	NA	2.50	0.00	Up
POX09323	Hypothetical protein	NA	4.89	0.00	Up
POX09324	Hypothetical protein	NA	1.06	0.00	Up
POX09325	Hypothetical protein	NA	3.60	0.00	Up
POX09326	Hypothetical protein	NA	1.85	0.00	Up
POX09327	Hypothetical protein	NA	4.20	0.00	Up
POX09331	Hypothetical protein	NA	1.47	0.00	Up
POX09332	Hypothetical protein	NA	1.79	0.00	Up
POX09334	Hypothetical protein	NA	1.31	0.00	Up
POX09335	Hypothetical protein	NA	2.15	0.00	Up

POX09336	Hypothetical protein	NA	1.94	0.00	Up
POX09337	Hypothetical protein	NA	1.44	0.00	Up
POX09338	Hypothetical protein	NA	1.60	0.00	Up
POX09339	Hypothetical protein	NA	1.04	0.00	Up
POX09340	Hypothetical protein	NA	1.32	0.00	Up
POX09345	Hypothetical protein	NA	1.18	0.00	Up
POX09346	Hypothetical protein	NA	2.71	0.00	Up
POX09348	Hypothetical protein	NA	1.51	0.03	Up
POX09350	Hypothetical protein	NA	2.47	0.04	Up
POX09352	alpha-amylase Amy13A	CBM20;GH13	1.00	0.00	Up
POX09354	Hypothetical protein	NA	1.12	0.00	Up
POX09357	Hypothetical protein	NA	1.54	0.00	Up
POX09358	Hypothetical protein	NA	3.20	0.00	Up
POX09360	Hypothetical protein	NA	1.80	0.00	Up
POX09361	Hypothetical protein	NA	2.08	0.00	Up
POX09363	Hypothetical protein	NA	2.16	0.00	Up
POX09364	Hypothetical protein	NA	1.28	0.00	Up
POX09365	Hypothetical protein	NA	2.00	0.00	Up
POX09368	Hypothetical protein	NA	-5.18	0.00	Down
POX09375	Hypothetical protein	NA	-2.03	0.00	Down
POX09381	Hypothetical protein	NA	-1.22	0.00	Down
POX09387	Putative chitinase	GH18	-2.32	0.00	Down
POX09389	Hypothetical protein	NA	1.47	0.00	Up
POX09397	Hypothetical protein	NA	-3.65	0.00	Down
POX09401	Hypothetical protein	NA	1.01	0.00	Up
POX09402	Hypothetical protein	NA	-1.12	0.00	Down
POX09405	Hypothetical protein	NA	-3.94	0.00	Down
POX09407	Hypothetical protein	NA	3.15	0.00	Up
POX09413	Hypothetical protein	NA	-1.45	0.00	Down
POX09418	Putative alpha-1,6-mannosyltransferase	GT32	-1.70	0.00	Down
POX09419	Hypothetical protein	NA	-4.30	0.00	Down
POX09422	Hypothetical protein	NA	-1.22	0.00	Down
POX09424	Hypothetical protein	NA	2.44	0.00	Up
POX09425	Hypothetical protein	NA	2.73	0.00	Up
POX09428	Hypothetical protein	NA	2.07	0.00	Up
POX09440	Hypothetical protein	NA	1.58	0.00	Up
POX09450	Hypothetical protein	NA	-2.35	0.00	Down
POX09454	Hypothetical protein	NA	2.64	0.00	Up
POX09463	Hypothetical protein	NA	-7.52	0.00	Down
POX09464	Hypothetical protein	NA	-7.05	0.00	Down

POX09465	Hypothetical protein	NA	-8.21	0.00	Down
POX09467	Hypothetical protein	NA	-6.85	0.00	Down
POX09468	Hypothetical protein	NA	-6.58	0.00	Down
POX09469	Winged helix repressor DNA-binding	NA	-8.82	0.00	Down
POX09470	Hypothetical protein	NA	-8.57	0.00	Down
POX09471	Sugar transporter, conserved site	NA	-5.39	0.00	Down
POX09472	Hypothetical protein	NA	-7.13	0.00	Down
POX09473	Hypothetical protein	NA	-8.38	0.00	Down
POX09474	Hypothetical protein	NA	-6.10	0.00	Down
POX09482	Hypothetical protein	NA	-5.80	0.00	Down
POX09486	Hypothetical protein	NA	-1.08	0.00	Down
POX09495	Hypothetical protein	NA	1.32	0.00	Up
POX09498	Major facilitator, sugar transporter-like	NA	-1.22	0.00	Down
POX09516	Zinc finger, C2H2-type	NA	-5.82	0.00	Down
POX09537	Hypothetical protein	NA	-1.33	0.00	Down
POX09541	Hypothetical protein	NA	1.02	0.00	Up
POX09543	Hypothetical protein	NA	1.21	0.00	Up
POX09554	Hypothetical protein	NA	2.33	0.00	Up
POX09575	Hypothetical protein	NA	-1.38	0.00	Down
POX09580	Hypothetical protein	NA	-2.72	0.00	Down
POX09598	Hypothetical protein	NA	2.90	0.01	Up
POX09605	Hypothetical protein	NA	2.62	0.00	Up
POX09615	Hypothetical protein	NA	-2.30	0.00	Down
POX09616	Hypothetical protein	NA	-1.75	0.00	Down
POX09626	DNA breaking-rejoining enzyme	NA	-1.81	0.00	Down
POX09627	Hypothetical protein	NA	-1.49	0.00	Down
POX09638	Hypothetical protein	NA	1.15	0.00	Up
POX09680	Hypothetical protein	NA	4.44	0.00	Up
POX09681	Hypothetical protein	NA	2.19	0.05	Up
POX09685	Aminoglycoside phosphotransferase	NA	-5.76	0.00	Down
POX09687	Hypothetical protein	NA	-4.71	0.00	Down
POX09691	Centromere protein B, DNA-binding region	NA	-1.04	0.00	Down
POX09695	Hypothetical protein	NA	-1.40	0.01	Down
POX09701	Hypothetical protein	NA	1.16	0.00	Up
POX09712	Reverse transcriptase	NA	-1.80	0.00	Down
POX09716	Hypothetical protein	NA	-1.54	0.03	Down
POX09728	Hypothetical protein	NA	-4.22	0.00	Down
POX09729	Zinc finger, CCHC-type	NA	-1.07	0.03	Down
POX09735	Hypothetical protein	NA	-1.25	0.00	Down
POX09743	Hypothetical protein	NA	-2.22	0.00	Down

POX09758	Hypothetical protein	NA	1.58	0.03	Up
POX09759	Hypothetical protein	NA	-1.10	0.00	Down
POX09766	Hypothetical protein	NA	-1.20	0.00	Down
POX09773	Hypothetical protein	NA	1.16	0.01	Up
POX09782	Hypothetical protein	NA	1.63	0.02	Up
POX09784	Conserved hypothetical protein	NA	-1.07	0.00	Down
POX09786	unnamed protein product	NA	-4.96	0.00	Down
POX09790	Hypothetical protein	NA	-3.84	0.00	Down
POX09795	Hypothetical protein	NA	-1.55	0.00	Down
POX09798	Hypothetical protein	NA	-3.04	0.00	Down
POX09800	Centromere protein B, DNA-binding region	NA	-1.05	0.01	Down
POX09801	Putative exo-beta-1,3-galactanase	GH43	-5.11	0.00	Down
POX09802	Putative pectin lyase	PL1	-2.42	0.00	Down
POX09805	Hypothetical protein	NA	3.53	0.00	Up
POX09806	Hypothetical protein	NA	1.33	0.03	Up
POX09812	Hypothetical protein	NA	-1.36	0.00	Down
POX09821	Hypothetical protein	NA	1.26	0.00	Up
POX09823	Hypothetical protein	NA	1.36	0.00	Up
POX09829	Hypothetical protein	NA	-5.43	0.00	Down
