# Supplementary material for: How an essential Zn2Cys6 transcription factor PoxCxrA regulates cellulase gene expression in ascomycete fungi?
Source: Biotechnol Biofuels. 2019 May 3;12:105. doi: 10.1186/s13068-019-1444-5 (PMC6498484; doi:10.1186/s13068-019-1444-5)
Supplement: Supplementary file 4 — Additional file 4: Table S3. Primers used in this study. [file 13068_2019_1444_MOESM4_ESM.pdf]

Additional file 4: Table S3. Primers used in this study.

**Primer name**	**Sequence（5'-3'）**
**Primers used for amplification of DNA binding domain-encoding DNA fragments**	
PoxCxrA$_{17-150}$-F	GGAATTCCATATGGCATGCGTGCTGTGTCA
PoxCxrA$_{17-150}$-R	ATAAGAATGCGGCCGCATGGCGCAGATCATCGTT
PoxClrB$_{1-120}$-F	AAGGCCATGGCTGATATCGGATCCATGTTCCACACCTTTGAAGGGT
PoxClrB$_{1-120}$-R	TCGACGGAGCTCGAATTCGGATCCTTACGTCGCCAACTCCAGCAG
PoxCxrA$_{11-114}$-F	AAGGCCATGGCTGATATCGGATCCATGACCGCCACCACCGTCAA
PoxCxrA$_{11-114}$-R	TCGACGGAGCTCGAATTCGGATCCTTACTCTTTCGACGGAAGCGCA
PoxCxrA$_{11-89}$-R	TCGACGGAGCTCGAATTCGGATCCTTAATCGTCACTGGGAGATCGATG
PoxCxrA$_{11-58}$-R	~~TCGACGGAGCTCGAATTCGGATCCTTAACGGGCCAATAATTCTCTCTCG~~
PoxCxrA$_{11-50}$-R	TCGACGGAGCTCGAATTCGGATCCCTCACACTGTACGTTGGCTCG
PoxCxrA$_{11-31}$-R	TCGACGGAGCTCGAATTCGGATCCTTACGTAAATTTCCCTGCGCAAAC
PoxCxrA$_{17-58}$-F	AAGGCCATGGCTGATATCGGATCCATGCCCCGACGCGTGCTGGC
PoxCxrA$_{17-58}$-R	~~TCGACGGAGCTCGAATTCGGATCCTTAACGGGCCAATAATTCTCTCTCG~~
PoxCxrA$_{11-150}$-F	AAGGCCATGGCTGATATCGGATCCATGACCGCCACCACCGTCAA
PoxCxrA$_{11-150}$-R	TCGACGGAGCTCGAATTCGGATCCTTAGCCATCGTCTCCAGAATTGACAG
PoxCxrA$_{17-58}$-M-F	AAGGCCATGGCTGATATCGGATCCATGCCCGCCGCCGTGCTGGC
PoxCxrA$_{17-58}$-M-R	TCGACGGAGCTCGAATTCGGATCCTTAACGGGCCAATAATTCTCTCTCG
**Primers used for amplification of the probes in electrophoretic mobility shift assay**	
POX05587-P-100bp-F	TCCTTCCTCATCTCCTCCACC
POX05587-P-R	FAM-TGTGATGGATTGGATCAAAGATC
POX05587-P-500bp-F	ATCACTCAGCCGCCATCTC
POX05587-P-300bp-F	FAM-GAGAGATCCACTACCCGCAA
POX05587-P-100bp-R	GAATGGATGATGAACACCAGAGT
PoxCxrA-P-300bp-F	CACTGTGTAGCAGCATATCGAAG
PoxCxrA-P-R	FAM-GCCGACGACTTGAGAAACTATT
PoxClrB-P-300bp-F	CGTTCGGTGCTCTTCTGCT
PoxClrB-P-R	FAM-GCTGCCGTTGGGTTTTCA
PBM1-F	CCCCTTTCATCAGATCCTCAA
PBM1-W-F	ACAAAATTGATCATCATCTCTTTTG
POX05587-P-R	FAM-TGTGATGGATTGGATCAAAGATC
PBM2-F	FAM-GAGAGGCGAATTGCTTCAGG
PBM2-R	GGGGTGGTGGAGGAGATGA
PBM2-W-R	TGATGAACACCAGAGTCGAGGT
PBM1-0-F	ATCAGATCCTCAAAGAACAAAATT

PBM1-3-F	AGATCCTCAAAGAACAAAATTGAT
PBM1-6-F	TCCTCAAAGAACAAAATTGATCAT
PBM1-9-F	TCAAAGAACAAAATTGATC
PBM1-12-F	AAGAACAAAATTGATCATCATCTCT
PBM1-3’	TTGAGGATCTGATGAAAGGG
PBM1-6’	AGGATCTGATGAAAGGGGGGT
PBM1-9’	ATCTGATGAAAGGGGGGTGG
PBM1-12’	TGATGAAAGGGGGGTGGTG
POX6051-1000-F	GTCGTGAAATGGCTGGAGTG
POX6051-1000-R	FAM-CCCAAGCAGAACTGACCAA
POX5915-1000-F	CCGAAAAGGTCGTTCTAGTCA
POX5915-1000-R	FAM-TCGTGTCTGTCTTGGGGAAC
POX4369-1000-F	CAAAGCGTCCAAGATAGCG
POX4369-1000-R	FAM-AGAGAATGGTTGAGCTGGAGG
POX7209-1000-F	AAGCAGTGTAAGTGAAGCAAAGG
POX7209-1000-R	FAM-AGGCCGTAGCTCAGAGTCTGT
POX7576-1000-F	TGCTTGCGGAATGTTGACC
POX7576-1000-R	FAM-AAGATGTGAGAAGGACCGTGAT
POX8783-1000-F	GAGGGAGTCATTGTAGCCGA
POX8783-1000-R	FAM-GAAAGAGGATCTGATCAACAGATGT
POX05587-P-F	GAGAGATCCACTACCCGCAA
POX05587-P-R	FAM-TGTGATGGATTGGATCAAAGATC
PoxClrB-P-300bp-F	CGTTCGGTGCTCTTCTGCT
PoxClrB-P-R	FAM-GCTGCCGTTGGGTTTTCA
PoxCxrA-P-300bp-F	CACTGTGTAGCAGCATATCGAAG
PoxCxrA-P-R	FAM-GCCGACGACTTGAGAAACTATT
PoxClrB-F	FAM-AGGAGACGAACAAATGAAA
PoxClrB-375-R	GAGGGTTGGACTCGGCAC
PoxClrB-355-R	TGGATGGTGGAGAAGAGGAC
PoxClrB-330-R	CTCCACCAGGGTTGCTTGATG
PoxClrB-310-R	GTGGATGAGCTCGACAAGGAC
PoxClrB-295-R	AAGGACTCAGCTGGGGGGA
PoxClrB-284-R	TGGGGGGAGGAGGAGGTG
PoxClrB-270-R	GGTGAGCAGCAAAAGAAGCA
PBM_PoxClrB-F	AGCTGAGTCCTTGTCGAGCT
PoxClrB-3’-R	GACTCAGCTGGGGGGAGG
PoxClrB-6’-R	TCAGCTGGGGGGAGG

PoxClrB-9'-R	GCTGGGGGGAGGAGGAG
PoxClrB-3-F	GAGTCCTTGTCGAGCTCAT
PoxClrB-R	FAM-GTACGGTAGCACCCAAAT
